# Cagrilintide lowers bodyweight through brain amylin receptors 1 and 3

**DOI:** 10.1016/j.ebiom.2025.105836

**Published:** 2025-07-03

**Authors:** Alexandra Oliveira Carvas, Andrea Leuthardt, Patricia Kulka, Greta Lommi, Shad Hassan, Bernd Coester, Sofia Lundh, Tune Pers, Anna Secher, Kirsten Raun, Thomas A. Lutz, Christelle Le Foll

**Affiliations:** a Institute of Veterinary Physiology, University of Zurich, Switzerland; b Novo Nordisk Foundation Center for Basic Metabolic Research, University of Copenhagen, Copenhagen, Denmark; c Global Drug Discovery, Novo Nordisk A/S, Måløv, Denmark

**Keywords:** Amylin, Hindbrain, Mouse, Weight, Food intake

## Abstract

**Background:**

Amylin (AmyR) and calcitonin (CTR) receptor co-agonists are currently in Phase II/III clinical trials for obesity treatment. Amylin binds to a heterodimeric receptor composed of CTR and the receptor activity modifying proteins 1, 2 or 3 (RAMP1-3).

**Methods:**

We investigated the role of amylin 1 and 3 (AMY_1_R, AMY_3_R) receptors in modulating the pharmacological effects of the dual amylin–calcitonin receptor agonists, cagrilintide and salmon calcitonin (sCT), in RAMP1/3 knockout (KO) mice. Male wild-type (WT) and KO littermate mice were fed high-fat diet for 23 weeks prior to the 3-week treatment period with vehicle, 150 nmol/kg sCT or 3 nmol/kg cagrilintide (subcutaneously, SID).

**Findings:**

Body weight loss was observed in WT cagrilintide-treated mice (−3.4 ± 0.51 g, *P <* 0.005; *n* = 8/group), whereas sCT rather increased it (0.60 ± 0.38 g, *P <* 0.01; *n* = 8/group). The absence of RAMP1 and RAMP3 impeded cagrilintide's potency but improved sCT's efficacy on weight loss. Cagrilintide and sCT both decreased food intake during the first few days of treatment in WT mice only (Day 1: vehicle 2.7 ± 0.2 g; cagrilintide 1.2 ± 0.1 g, *P <* 0.0001; sCT 1.5 ± 0.2 g, *P <* 0.0021; *n* = 7–8/group). Both peptides activated cFos signal in neurons of the dorsal vagal complex (DVC) and lateral parabrachial nucleus (LPBN) of WT mice while AP cFos signal was decreased in cagrilintide-treated RAMP1/3 KO mice by 57% compared to WT cagrilintide-injected mice (*P <* 0.001, *n* = 5–6/group). Differential gene expression was analysed in the DVC, LPBN and mediobasal hypothalamic area of WT and RAMP1/3 KO mice. After 3 weeks of treatment, neither sCT nor cagrilintide significantly altered gene expression in the DVC or LPBN in WT mice. However, mRNA bulk sequencing points to a role of RAMP1/3 in synaptic function and receptor trafficking.

**Interpretation:**

Altogether, these results demonstrate the dependency of cagrilintide on AMY_1_R and AMY_3_R to lower body weight.

**Funding:**

This work was supported by an investigator led Novo Nordisk Consortium grant, Swiss National Foundation and the 10.13039/501100006447University of Zurich.

**Research in context**

**Evidence before this study**

Cagrilintide, a long-acting and stable analogue of amylin, is in investigation for treatment of obesity and type 2 diabetes in combination with the long acting glucagon like peptide −1 (GLP-1) receptor agonist semaglutide. In a dose-finding phase 2 trial, cagrilintide demonstrated a dose-dependent reduction in body weight and waist circumference in people with obesity. While several *in vitro* studies have investigated the specificity and receptor dynamics of cagrilintide, there remains a significant gap in understanding the individual receptor contribution to the *in vivo* effects. The use of an amylin receptor knockout mouse model provides a valuable approach for assessing the *in vivo* specificity of amylin receptor agonists. This effect will be compared to sCT, a dual AMYR- CTR agonist, also commonly used as a structural backbone for the development of amylin receptor agonists.

**Added value of this study**

In this series of *in vivo* and *ex vivo* studies, we demonstrate that subchronic cagrilintide application reduces food intake and body weight by reducing relative fat mass while maintaining relative lean mass. These effects are accompanied by activation of neurons of the area postrema of the hindbrain via the amylin receptor subtypes 1 and 3. In contrast, subchronic sCT administration produces the opposite effect, increasing body weight despite also eliciting hindbrain neuronal activation when applied acutely. Bulk mRNA sequencing of the key brain areas targeted by these peptides showed that the RAMP 1 and 3 subunits of the amylin receptor, play a crucial role in neurotransmitter and synaptic activity. The opposing effects of cagrilintide and sCT along with previously published *in vitro* data suggest differences in receptor dynamics that lead to distinct control of energy homoeostasis.

**Implications of all the available evidence**

This pre-clinical study is highly relevant as cagrilintide is the most advanced amylin receptor agonist that is undergoing clinical trials with great chance to be marketed alone or in combination with the GLP-1 receptor agonist semaglutide to elicit a greater weight loss. While *in vitro* studies have assessed cagrilintide action at the receptor level, this study is the first one evaluating in mice its metabolic and signalling effects.

## Introduction

The obesity rate has been dramatically increasing for decades, reaching pandemic proportions. In 2025 approximately 1 billion people live with obesity worldwide.^1^ In 2017–2018, more than one in five children or adolescents in the United States fell into the category of obesity implying that this issue is not just limited to adults anymore, as it is also becoming increasingly prevalent among younger generations.^1^ Obesity presents a significant health challenge due to its strong association with various diseases, including cardiovascular, metabolic diseases, osteoarthritis, depression, sleep apnoea, and some types of cancers.^2^^,^^3^ The increasing prevalence of obesity and its status as a chronic disease^4^ underscores the importance of developing new therapeutic approaches, such as the long-acting human amylin analogue cagrilintide, which is currently under clinical development in combination with semaglutide for obesity and T2D.^5^^,^^6^ But first, it is necessary to gain a better understanding of the pharmacological mechanisms involved.

Endogenous amylin is a centrally acting peptide hormone that was discovered more than three decades ago in patients with T2D and diabetic cats.^7^ It is co-secreted with insulin by pancreatic beta-cells in response to a meal and increasing glucose levels, and it is released into the blood circulation. Amylin's primary property is to reduce eating^8^ and to inhibit postprandial glucagon secretion.^9^^,^^10^ For its physiological actions, amylin targets various regions of the central nervous system; its primary site of action is the area postrema (AP), a circumventricular organ located in the caudal hindbrain, which is crucial in promoting amylin's satiating effect.^11^, ^12^, ^13^, ^14^ From the AP, the signal propagates to the nucleus of the solitary tract (NTS) and lateral parabrachial nucleus (LPBN). Amylin also targets agouti-related peptide (AgRP) and pro-opiomelanocortin (POMC) neurons located in the arcuate nucleus of the hypothalamus (ARC) independently of the AP where it may contribute to the control of energy balance by altering energy expenditure.^10^^,^^15^, ^16^, ^17^, ^18^, ^19^

To exert its effects, amylin binds to heteromeric receptor complexes composed of the CTR and specific RAMPs 1–3, which form three distinct amylin receptor subtypes AMY_1_R, AMY_2_R and AMY_3_R. While CTR has a higher affinity for calcitonin, it exhibits a higher affinity for amylin when combined with RAMP.^20^ The density and prevalence of CTR and the RAMP subtypes differ among various brain areas, with the highest prevalence and demonstrated co-localisation in individual neurons in the AP.^21^^,^^22^

Cagrilintide was developed based on structure–activity relationship with the aim of generating a long-acting non-fibrillating amylin analogue with retained affinity for AMY_3_R.^23^ Cagrilintide along with the other amylin analogue currently in clinical use, pramlintide,^24^^,^^25^ represent significant advancement in the field of amylin analogues. Pramlintide is a human amylin analogue approved as an add-on to insulin therapy for treatment of diabetes mellitus. It promotes better glycaemic control and small but significant weight loss.^26^^,^^27^ The objective with cagrilintide was to create a long-acting amylin analogue that effectively reduces body weight, while minimising adverse effects such as nausea and vomiting.^23^ Cagrilintide and sCT have a similar pharmacological profile *in vitro*, but cagrilintide displays a shorter residence time at the CTR suggesting a different pharmacodynamic profile.^28^^,^^29^ To date, both clinical and preclinical studies have shown outcomes regarding the impact of cagrilintide on weight loss, whether administered alone or in combination with a GLP-1 receptor agonist such as semaglutide.^5^^,^^30^, ^31^, ^32^, ^33^, ^34^

This study seeks to enhance our comprehension of the distinct effects, at the *in vivo* and *ex vivo* levels, produced by the two receptor systems, amylin (AMYR) and calcitonin (CTR), and consequently, to unravel the role of RAMPs in modulating the subchronic pharmacological effects and potential disparities between cagrilintide and sCT.

## Methods

### Animals and husbandry

RAMP 1/3 double knockout (KO) (129S6/SvEv Ramp1/3<tm1>), RAMP1 KO (129S6/SvEv Ramp1<tm1>), RAMP3 KO (129S6/SvEv Ramp3<tm1>) founder mice on a 129S2/SvEv background.^35^, ^36^, ^37^ RAMP1 KO and RAMP3 KO are maintained on a heterozygous breeding scheme while RAMP1/3 KO are maintained on a KO breeding scheme. RAMP1/3 KO were regenerated using 129S2 mice and bred at Janvier Elevage (France). WT mice were age matched and issued from the single KO colonies or from animal provider (129S2, Janvier Elevage, France).

#### Study 1: Acute study in WT chow fed mice

Thirty-two 12 week-old male WT 129S2/SvEv mice (Janvier Elevage, France) were individually housed in food monitoring cages (BioDAQ, New Brunswick, NJ, USA). Mice were housed in a controlled environment maintained at 21 ± 2 °C, under a 12/12 h light–dark cycle (lights off at 10.00 h). They were adapted to the cages and hoppers for 2 weeks before being tested. Mice were fed water and chow diet ad libitum (3.14 kcal/g of food; Kliba, Switzerland, cat# 3436).

#### Study 2: Subchronic study in WT high fat diet fed mice

Male mice were housed in a controlled environment maintained at 21 ± 2 °C, under a 12/12 h light–dark cycle (lights off at 10.00 h). Mice had ad libitum access to standard chow (3.14 kcal/g of food; Kliba, Switzerland, cat# 3436) and water, until 3 weeks old. Thirty-two 3 week-old male WT 129S2/SvEv mice (Janvier Elevage, France) were fed 60% high fat diet (HFD, 5.24 kcal/g of food; SSNIFF Spezialdiäten GmbH, Germany, cat# EF D12452) under group housing conditions for 19 weeks. They were then fed 45% HFD for 6 weeks (4.73 kcal/g of food; SSNIFF Spezialdiäten GmbH, Germany, cat# EF D12451) until the end of the study. The mice were group housed (3–4 mice per cage) until 1 week before the 21-day treatment period with cagrilintide.

#### Study 3: Subchronic study in WT and RAMP1/3KO mice

Male mice were housed in a controlled environment maintained at 21 ± 2 °C, under a 12/12 h light–dark cycle (lights off at 11.00 h). Mice had ad libitum access to standard chow (3.14 kcal/g of food; Kliba, Switzerland, cat# 3436) and water, until 3–4 weeks old. Twenty-four WT and twenty-four RAMP 1/3 KO male mice were fed a 60% fat HFD (5.24 kcal/g of food; SSNIFF Spezialdiäten GmbH, Germany, cat# EF D12452) provided ad libitum for 18 weeks. Then mice were fed to a 45% fat HFD (4.73 kcal/g of food; SSNIFF Spezialdiäten GmbH, Germany, cat# EF D12451) provided ad libitum for 6 weeks until the end of the study. The mice were group housed (3–4 mice per cage) until 1 week before the 21-day treatment period. Each cage was equipped with a red plastic house, tissue paper, nest-building material and wood shavings. Prior to the 21-day treatment, mice were injected with vehicle for three days to acclimate to the daily subcutaneous injections and reduce stress associated with the injections.

#### Study 4: Acute study in WT, RAMP1KO and RAMP3KO and RAMP1/3KO male mice

Mice were group housed in a controlled environment maintained at 21 ± 2 °C, under a 12/12 h light–dark cycle (lights off at 11.00 h). In the first cohort, 25 male WT and 25 male RAMP1/3 KO mice aged between 12 and 20 week-old were fed chow diet and maintained under group housing condition. In the second cohort, 18 male WT, 18 male RAMP1 KO, 18 male RAMP3 KO and 18 male RAMP1/3 KO mice aged between 10 and 15 week-old were fed chow diet and maintained under group housing condition.

### Genotyping

As previously published,^37^ DNA was extracted from toe biopsies after addition of 200 μl of 50 mM NaOH, heated at 95 °C and shaken at 800 rpm for 35 min, samples were then neutralised with 200 μl of 500 mM Tris–HCl, pH 5.5. One μl of DNA was then combined with the respective primers and GoTaq polymerase (Promega AG, Switzerland, cat# A6001). Animals were genotyped using the following primers: RAMP1 forward TCATGGGGACCTTTAGGTAAGC, RAMP1 reverse ACAGCAATCCTTCT ACCTCAACAC, RAMP3 WT band is detected using R3-1: GTGCTCAAGGGTTCTGTCTG and R3-10: GACCTGGTTCATCTCTGGCTCC and RAMP3 null band is detected using R3-10: GACCTGGTTCATCTCTGGCTCC and neo-60: GCTTCCTCTTGCAAAACCACA.

### Peptides and pharmacokinetics

All experimental drugs were provided by Novo Nordisk Pharma A/S. Vehicle: 5 mM NaAcetate, 0.007% Tween-20, 240 mM propylene glycol pH4. Vehicle, sCT and cagrilintide were injected at a volume of 5 mL/kg and at doses specified for each study. Functional potency of cagrilintide and sCT in humans,^23^ rats^23^ and mice CTR(a), AMY_1_R and AMY_3_R are indicated in Table 1.

**Table 1:** Functional potency of the cagrilintide and sCT in humans, rats and mice AMYR3 and CTR(a) receptors.

Functional assay pEC50 [M] [95% CI]						
	Luciferase^23^		cAMP^23^		cAMP	
	hAMYR3	hCTR(a)	rAMYR3	rCTR(a)	mAMYR3	mCTR(a)
Cagrilintide	10.31 [0.05]	10.21 [0.07]	9.46 [0.03]	9.54 [n.d]	10.03 [0.31]	10.01 [0.3]
sCT	11.79 [0.14]	11.54 [0.19]	12.28 [0.34]	12.85 [0.21]	10.98 [0.36]	11.11 [0.36]

EC50 values were calculated in GraphPad Prism using a nonlinear regression and pEC50 values calculated as -LogEC50. Cagrilintide and sCT activates CTRA and AMY3R with equal potency for each species. Cagrilintide's potency is reduced in human, mouse and rat compared to sCT.

#### Pharmacokinetics assessment in cells lines transfected with mouse receptors components

The mouse receptor assay was performed using a cAMP-based BacMam screen. BacMam (mCTR(a) NM_001042725; mRAMP3 NM_019511.3, Life Technologies, USA, Cat# M4395) transfected HELA cells (ATTC, USA Cat# CCL-2™) were stimulated with cagrilintide and sCT. Increasing levels of endogenous cAMP were measured as a reduction of fluorescence resonance energy transfer (FRET) between Europium-cryptate-conjugated anti-cAMP antibody and d2-conjugated cAMP (CisBio Bioassays, France, cat# 62AM4PEC). The fluorescence ratio was plotted as a function of the concentration of compound. Data were analysed of a four-parameter logistic function with shared top and bottom using GraphPad Prism software (Version 8.0). Please refer to^23^ or^24^ for the human and rat assays respectively.

### Peptide injection protocol

#### Acute response to 3 doses of cagrilintide in 129S2 WT chow fed mice (study 1)

Mice were injected subcutaneously (s.c.) at dark onset after a 4 h fast in the light phase with vehicle, 3 nmol/kg, 30 nmol/kg or 300 nmol/kg of cagrilintide.

#### Subchronic injection of 3 doses of cagrilintide in 129S2 WT high fat diet fed mice (study 2)

Mice were s.c. injected 1 h before dark onset for 21 days with vehicle, 0.3 nmol/kg, 3 nmol/kg or 30 nmol/kg of cagrilintide. Body weight and food intake were obtained daily just before the injection. Mice were sacrificed 24 h after the last dose under pentobarbital anaesthesia (100 mg/kg, i.p., Cantonal Pharmacy of Zurich, Switzerland).

#### Subchronic treatment in 129S2 WT and RAMP1/3 KO high fat diet fed mice (study 3)

At the age of 25 weeks, male RAMP 1/3 KO and WT mice were semi-randomised by body weight and assigned to their respective treatment group (*n* = 8/group): vehicle; sCT (150 nmol/kg/d); cagrilintide (3 nmol/kg/d). Mice were s.c. injected once a day for 3 weeks 1 h before dark onset between 9.00 and 10.00 h. Prior to injection, daily body weight and food intake were measured manually. On the day of sacrifice, mice were anesthetised with pentobarbital (100 mg/kg, i.p., Cantonal Pharmacy of Zurich, Switzerland) 24 h after receiving the last dose of peptide.

Brains were harvested, snap frozen on dry ice and kept at −80 °C until mRNA extraction for next-generation sequencing (NGS) analysis. Blood samples were collected from the right ventricle of the heart during the sacrifice in 1 mL EDTA-coated tubes containing dipeptidyl peptidase-4 inhibitor (10 μL/mL blood; Sigma Aldrich, Switzerland, cat# DPP4-010) and protease inhibitor (30 μL/mL blood; Sigma Aldrich, Switzerland, cat# P2714). After centrifugation, plasma was kept at −80 °C. The mice were stored at −20 °C until body composition analysis was performed.

### Measurement of body composition using magnetic resonance imaging (MRI)

Lean and fat mass were measured post-mortem using EchoMRI (EchoMRI LLC, USA) at the Zurich Integrative Rodent Physiology (University of Zurich). Calibrations and measurements were performed according to manufacturer's instructions. Two measurements were performed and averaged per mouse. Fat mass and lean mass values are expressed as percent of body weight.

### Biochemical analysis

Plasma glucose was measured at sacrifice using a glucometer. Plasma insulin and leptin were measured in duplicates using the U-PLEX Custom Metabolic Group 1 mouse metabolic assay (MesoScale Diagnostics MD, USA, cat# K152ACM) following the manufacturer's instructions.

### Next**-**generation sequencing of the AP/NTS, LPBN and VMH brain nuclei in WT and RAMP1/3 KO mice treated for 21 days with vehicle, sCT or cagrilintide (study 3)

#### mRNA extraction

Brains were mounted on a cryostat (Leica Biosystems, Germany, cat# CM3050S) and trimmed from caudal to rostral until the area of interest was reached. 1 mm^3^ brain punches were performed (Zivic instrument, USA, cat# PUN1000). Consequently, the first region to be punched was DVM (AP/NTS), followed by LPBN and MBH (ARC/VMN), with both LPBN and MBH being punched bilaterally. Brain tissue was promptly frozen using liquid nitrogen and subsequently stored in a freezer set to −80 °C.

mRNA was isolated using Promega RNA extraction kit (Promega AG, Switzerland, cat# Z6211) according to the manufacturer's protocol. Briefly, brain tissue samples were kept on ice to prevent RNA degradation at room temperature. Samples were dissolved in a mixture of lysis Buffer (LBA), 1-Thioglycerol (TG) and after adding 100% isopropanol, they were removed from ice for further processing. The extraction process included washing steps with pre-mixed RNA Wash Solution and Column Wash Solution. Additionally, the DNAse master mix was added to degrade DNA contaminants. Short centrifugation steps were performed between each step. mRNA were eluted from the mini-column by adding 15 μL of nuclease-free water. Proper temperature control and adherence to protocol guidelines were crucial for obtaining high-quality mRNA throughout the process.

#### Quality control and sequencing

After extracting all the samples, mRNA levels and sample purity were measured using the Nanodrop (Agilent, Switzerland). mRNA quality control was evaluated through electrophoresis measurements (UZH Functional Genomic Center, Agilent, Switzerland, TapeStation 4200). A minimum RNA concentration of 30 μg/μL and a RIN >8 were necessary for mRNA next-generation sequencing (NGS). Out of 138 brain samples, 117 met these criteria for NGS analysis, which was also conducted by the UZH Functional Genomic Center. Briefly, mRNA libraries were produced using the Illumina NovaSeq platform where single-end reads were sequenced to a depth of approximately 20–25 × 10^6^ reads per sample. 6 replicates per conditions were performed.

#### Data analysis

Datasets were analysed by the UZH Functional Genomic Center and the University of Copenhagen. The first analysis was performed using the supporting user for shell script integration-SUSHI pipeline (pipeline produced by the UZH Functional Genomic Center^38^). A quality control of the raw sequences produced by the instrument (technical quality control) was performed. A quality control step followed by an alignment to a reference transcriptome was subsequently performed on the counted reads using CountQCApp from SUSHI; 3 AP samples, 1 LPBN sample and 2 ARC samples were removed from the comparative analysis. Abundance of transcript for each brain area was determined with KallistoApp from SUSHI. The differentially expressed genes (DEG) were then analysed using the DESeq2App. All samples were compared to the WT vehicle-treated group. Intergroup comparison was performed using the MulitDEG app.

The second analysis was conducted on the entire dataset for each brain region as previously published.^39^ Weighted gene co-expression network analysis: WGCNA was run using the R implementation (Version 4.4.2). Only genes with counts ≥5 in at least 70% of the samples were included in the analysis. Variance stabilising transformation normalisation was then applied using DESeq2. Using the biweight midcorrelation, a similarity matrix was computed from which a signed network was constructed using a soft-thresholding power of 14 (MBH, DVC) or 16 (LPBN), maximising the scale-free topology R2 fit while keeping mean connectivity to a value ∼100. Genes were clustered hierarchically based on the average topological overlap measure, and modules of coexpressed genes were identified using the ‘cutreeDynamic’ function with the parameters ‘minClusterSize’ set to 30, ‘deepSplit’ set to three and ‘pamStage’ set to false. Finally, the Pearson correlation between the module eigengenes was computed, and modules with a correlation above 0.70 were merged.

Module and treatment associations: The association between module eigengenes and knockout/treatment conditions was tested using a linear model via the limma R package (Version 4.4.2). For each module, a linear model was fitted with the module eigengene as the dependent variable and a design matrix incorporating knockout and treatment conditions as independent variables. Model contrasts were applied to compare knockout vs non-knockout conditions. Significance was assessed using empirical Bayes moderation and *P*-values were adjusted for multiple testing using the Benjamini-Hochberg (BH) method.

Cell population enrichment for module genes: Cell population marker genes (ESμ >0) were tested for enrichment of module genes using a one-tailed Fisher's exact test. *P* values were adjusted for multiple testing using the Bonferroni method (adjusting for the number of cell populations Å∼ the number of modules).

Gene Ontology analysis: Modules were tested for enrichment of GO terms (categories: cellular component, biological process, molecular function) using the clusterProfiler R implementation. For each brain region, GO enrichment analysis was carried out on all significant modules. Thresholds were set at *P* = 0.01 and q = 0.05.

### Assessment of neuronal activation in the AP/NTS and LPBN following vehicle, sCT or cagrilintide treatments in WT and RAMPs KO mice (study 4)

*Cohort 1*: WT and RAMP1/3 KO were injected i.p. at dark onset after a 12 h fast in the light phase with vehicle, sCT (3 nmol/kg or 150 nmol/kg) or cagrilintide (3 nmol/kg) (*n* = 5–6/group).

*Cohort 2*: WT, RAMP1 KO, RAMP3 KO and RAMP1/3 KO were injected i.p. at dark onset after a 12 h fast in the light phase with vehicle or cagrilintide (3 nmol/kg) (*n* = 6/group).

For both cohorts, animals were deeply anesthetised 90 min later (pentobarbital 100 mg/kg, i.p) and transcardially perfused with cold 0.1 M of phosphate buffer (PB) for 1.5 min followed by 4% of paraformaldehyde (PFA) in PB for 2.5 min. Brains were removed and postfixed overnight in 4% of PFA and cryoprotected in 20% of sucrose-PB for 24 h. Brains were frozen in hexane on dry ice and stored at −80 °C until sectioning. Stereological brain sampling of the areas of interest was performed and brain were serially cut on a cryostat (Leica Biosystems, Germany, cat# CM3050S), mounted on Superfrost Plus slides (Thermo Fisher Scientific, Reinach, Switzerland), and cryoprotected in 50% of PB, 30% of ethylene glycol, 20% of glycerol, and subsequently stored at −20 °C.

### cFos immunostaining

Blocking of the mouse brain sections was performed with 0.4% Triton–4% normal goat serum in PBS for 2 h at room temperature. AP/NTS and LPBN sections were incubated at 4 °C for 48 h with rabbit anti-Fos antibody (1:500, Cell signalling technology, Switzerland, Cat# 2250, RRID: AB_2247211), and then after PBS washes, incubated with goat anti-rabbit Alexa Fluor CY3 (1:200, LuBio Science GmbH, Switzerland for Jackson Immunoresearch, Cat# 111-165-144) or alexa fluor 488 for 2 h at room temperature. After thorough washing in PBS, the sections were counterstained with DAPI, mounted immediately and coverslipped with Vectashield antifade Mounting Medium (Vectorlabs, Burlingame, USA, cat# H-1400).^40^

### Quantification and analysis of immunostaining

Photomicrographs of all sections containing AP, NTS and LPBN were taken at 20×  magnification using Zeiss Axio Scan.Z1 slidescanner (Center for Microscopy and Image Analysis UZH, Zeiss, Germany). All images for each study were acquired using the same exposure time. Scanned images were imported into QuPath (QuPath 0.5.0) for quantification. Three to four sections were counted and averaged per area of interest. A polygon tool was used manually to determine the boundary of the region of interest of the brain section. A cell detection tool was used to detect every cell in the section by using a built-in cell segmentation algorithm. The cell detection tool was used on the DAPI channel to make sure that only a cell was recognised and not the background or artefact by system. A detection classifier was created by using the create detection classifier function. The numbers of positive counted DAPI cells containing a cFos signal were automatically counted within the region. The experimental group was blinded to the experimenter during the analysis.

### Ethics

All animal experimental protocols in this study were approved by the University of Zurich Animal Protection Office and Ethics committee, the Cantonal Veterinary Office of the Canton of Zurich (licences n° 102/2018 and 136/2021), and conform to Swiss Animal Protection guidelines and regulations (Swiss Animal Protection and Swiss Animal Act and Ordinance) and in accordance with the EU Directive on the protection of animals used for scientific purposes. The reporting of these studies adheres to the ARRIVE guidelines.

### Statistics

Sample size was estimated using G-power.^41^ All analyses were performed using GraphPad Prism software (GraphPad Prism Version 10.3, San Diego, CA) and R studio (Version 2024.12.0 + 467). Data were checked for normality using the normality QQ plot. In experiments comparing independent treatment groups, significance was tested using one-way ANOVA. When more than one factor was compared, two-factor or three-factor ANOVA with or without repeated measure (RM) was performed, followed by Tukey's multiple comparisons test as recommended. A *P*-value <0.05 was considered statistically significant. All data are presented as mean ± SEM. Information on replicates and significance are reported in the figure legends.

### Role of funders

Novo Nordisk employees (K.R., A.S., S. L.) had a consulting role in the study design, interpretation of the data and approved the final version of the manuscript. Novo Nordisk had no role in the data collection or data analyses.

## Results

All statistical analysis for the *in vivo* studies are reported in Supplementary Materials.

### Determination of the optimal dose of cagrilintide dose in chow-fed and HFD-fed WT mice

In the first study, the acute response to three doses of cagrilintide were compared to the vehicle in WT 129S2 chow-fed mice. All doses of cagrilintide significantly reduced eating; the doses of 30 and 300 nmol/kg produced the highest anorectic response and lowered 24 h food intake by 51% and 57% respectively, as compared to vehicle (*P* < 0.001. two-way RM ANOVA; Supplementary Fig. S1a). Consequently, the body weight loss in mice treated with cagrilintide at a dose of 30 and 300 nmol/kg groups was significantly higher as compared to the vehicle group (*P* < 0.05. one-way ANOVA; Supplementary Fig. S1b).

**Fig. 1:**
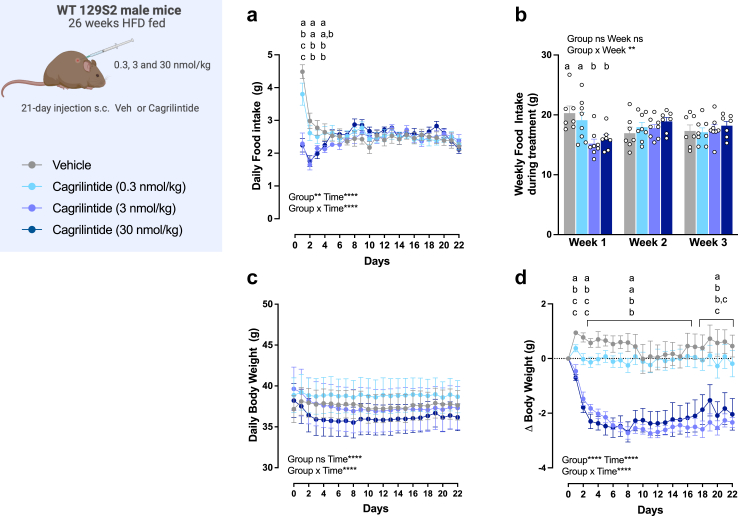
**Determination of the appropriate cagrilintide dose in HFD-fed WT mice in a subchronic application. a**: daily food intake (g) of WT high fat diet male mice treated for 21 days with vehicle or cagrilintide (0.3, 3 and 30 nmol/kg); **b**: weekly food intake (g); **c**: daily body weight (g) of WT high fat diet fed male mice treated for 21 days with vehicle and cagrilintide (0.3, 3 and 30 nmol/kg); **d**: cumulative daily body weight change (g). Data are represented as mean ± SEM, *n* = 8/group. Statistics: data were analysed by two-way ANOVA with or without repeated measured (factors: time and treatment group) followed by Tukey post-hoc comparison; ∗*P* < 0.05, ∗∗*P* < 0.01, ∗∗∗*P* < 0.001, ∗∗∗∗*P* < 0.0001. ^a, b, c^ groups with different superscripts are different from each other at each time point at *P* < 0.05 or more.

In the second study, 3-week-old WT 129S2 high-fat diet fed mice were tested for their food intake (Fig. 1a and b) and body weight response (Fig. 1c and d) to subchronic injections of increasing doses of cagrilintide compared to vehicle. Cagrilintide at a dose of 3 and 30 nmol/kg significantly decreased food intake on day 1, 2 and 3 of treatment as compared to vehicle-treated mice and mice receiving the 0.3 nmol/kg dose (*P* < 0.05. two-way ANOVA; Fig. 1a). After the first three days of treatment, cagrilintide was not able to elicit anorexia whatever the dose (Fig. 1a and b). Cagrilintide doses of 3 and 30 nmol/kg actively decreased body weight from day 1 to day 8 as compared to vehicle-treated mice (*P* < 0.05. two-way RM ANOVA; Fig. 1c and d) and the lower body weight was maintained until the end of the experiment. Mice treated with the 0.3 nmol/kg dose showed a significant body weight loss only for the first two days (*P* < 0.05. two-way RM ANOVA; Fig. 1d). As a similar effect on eating and body weight was observed with the 3 and 30 nmol/kg dose, the lowest dose producing a significant effect (3 nmol/kg) was selected for further investigations.

### Cagrilintide sustainably lowers body weight in WT mice and is dependent on RAMPs

**Fig. 2:**
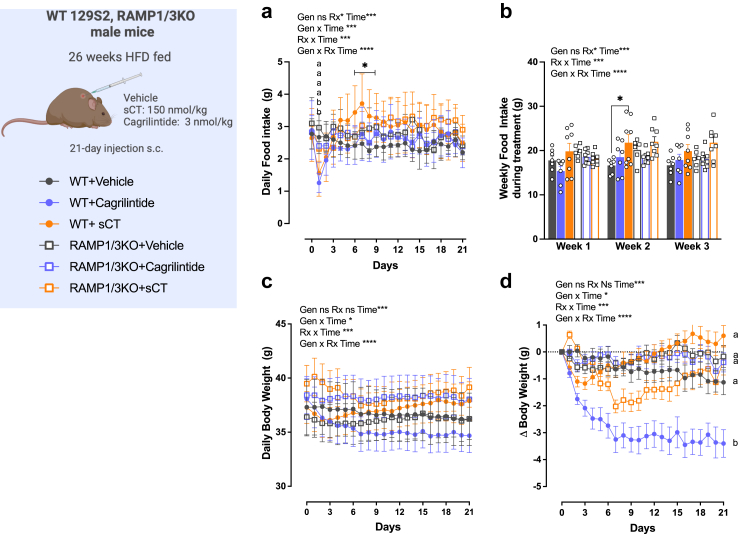
**Effect of a 3-week cagrilintide treatment in HFD-fed RAMP1/3 KO and WT male mice on food intake and body weight. a**: daily food intake treated of WT and RAMP1/3 KO HFD-fed male mice treated for 21 days with vehicle, cagrilintide (3 nmol/kg) or salmon calcitonin (sCT, 150 nmol/kg); **b**: weekly food intake (g); **c**: daily body weight (g); **d**: cumulative daily body weight change (g). Data are represented as mean ± SEM, *n* = 7–8 per group. Statistics: data were analysed by three-way ANOVA (factors: Genotype (Gen), Treatment (Rx) and time) with repeated measured followed by Tukey's post-hoc comparison; ∗*P* < 0.05, ∗∗*P* < 0.01, ∗∗∗*P* < 0.001, ∗∗∗∗*P* < 0.0001. ^a, b, c^ groups with different superscripts are different from each other at each time point at *P* < 0.05 or more.

To assess whether the effects of cagrilintide on eating and body weight are mediated through the receptor AMY_1_R or AMY_3_R, WT and RAMP 1/3 KO high-fat diet fed mice (Supplementary Fig. S2) were allocated to vehicle, sCT, and cagrilintide treatment groups. During the treatment period of three weeks, mice were monitored daily for food intake and body weight (Fig. 2). The initial body weight was comparable among all groups, with no significant differences at the beginning of the experiment (Supplementary Fig. S2).

Cagrilintide and sCT exerted an acute anorectic effect only on day 1 of treatment in WT mice and decreased food intake by 53% and 42% respectively (*P* < 0.01. three-way ANOVA; Fig. 2a) while no effect was observed in RAMP1/3 KO mice. For the rest of the study, cagrilintide demonstrated no significant impact on cumulative or weekly food intake in WT or RAMP 1/3 KO mice (Fig. 2b). Despite the lack of a lasting effect on food intake, cagrilintide elicited a sustained and noteworthy reduction in body weight in WT mice (day 21 body weight loss: 3.4 g or −66% vs WT Veh; *P* < 0.001. three-way ANOVA; Fig. 2d) but had no effect in KO mice (Fig. 2c and d) suggesting that AMY_1_R and AMY_3_R are necessary to mediate the body weight loss in response to cagrilintide.

sCT administration triggered distinct responses on body weight in both WT and KO mice. During the initial three days, sCT lead to body weight loss in WT mice, while promoting weight gain in KO mice (Fig. 2d). Consistent with our prior publication,^42^ subchronic sCT treatment then caused a reduction in body weight in RAMP 1/3 KO mice but resulted in a modest weight gain of approx. 2 g in WT mice (Fig. 2d). Notably, subchronic sCT treatment in WT mice also lead to an increase in food intake, which was significant on day 8–10 of treatment (+50%, *P* < 0.05 vs WT + veh. three-way ANOVA; Fig. 2a and b), suggesting an influence on feeding circuits. These findings underscore the differential impact of sCT on body weight and food intake between these two mouse groups.

### Cagrilintide decreases fat mass without affecting lean mass in WT mice

**Fig. 3:**
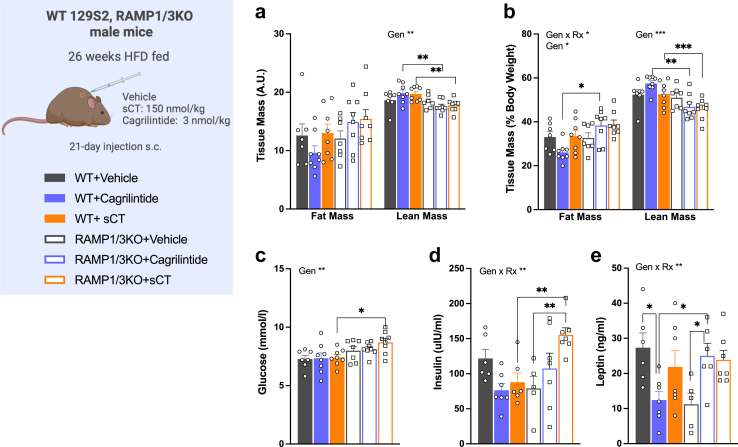
**Effect of a 3-week cagrilintide treatment HFD-fed RAMP1/3 KO and WT male mice on body composition and plasma parameters. a**: fat mass and muscle mass (g) of WT and RAMP1/3 KO mice treated for 21 days with vehicle, cagrilintide (3 nmol/kg) or sCT (150 nmol/kg); **b**: fat mass and muscle mass expressed as percent of body weight; **c**: plasma glucose levels (mmol/L) at sacrifice; **d**: plasma insulin levels (uIU/mL) at sacrifice; **e**: plasma leptin levels (ng/mL) at sacrifice. Data are represented as mean ± SEM, *n* = 7–8 per group. Statistics: data were analysed by three-way ANOVA (factors: Genotype (Gen), Treatment (Rx) and time) followed by Tukey's post-hoc comparison; ∗*P* < 0.05, ∗∗*P* < 0.01, ∗∗∗*P* < 0.001.

The comparison of body composition of WT and RAMP 1/3 KO mice following a three-week subcutaneous treatment with vehicle, cagrilintide, or sCT under HFD feeding revealed distinct patterns. In WT mice, although cagrilintide treatment led to significant weight loss, the changes in body composition compared to vehicle-treated mice did not reach statistical significance, with fat mass decreasing by 7% (*P* = 0.2) and lean mass increasing by 5% (*P* = 0.14. two-way ANOVA; Fig. 3a and b). Alongside these changes, plasma leptin levels decreased by 2-fold (*P* < 0.05. two-way ANOVA; Fig. 3e). In WT mice, sCT treatment had no effect on body composition despite the final weight gain.

In contrast, RAMP 1/3 KO mice exhibited less alterations in body composition in response to both sCT and cagrilintide. While cagrilintide did not significantly alter fat or lean mass in RAMP 1/3 KO mice compared to vehicle-treated KO mice (Fig. 3b), RAMP1/3 KO mice receiving cagrilintide showed increased relative fat mass (+32%, *P* < 0.05; Fig. 3b) and decreased relative lean mass (−22%, *P* < 0.01. two-way ANOVA; Fig. 3b) along with elevated leptin levels (*P* < 0.05; Fig. 3d) compared to their WT-treated counterparts. sCT treatment reduced relative lean mass by 15% in KO mice compared to WT (*P* < 0.05. two-way ANOVA; Fig. 3b), despite no significant differences in body weight at the end of the study (Fig. 2c and d). Additionally, RAMP 1/3 KO mice treated with sCT displayed higher insulin plasma levels at sacrifice (*P* < 0.01. two-way ANOVA; Fig. 3d). Altogether, cagrilintide treatment seems to lower body weight by acting on fat depots while maintaining lean mass while sCT does not induce such effects. These results support the findings that subchronic cagrilintide application influences nutrient utilisation and partitioning in an opposite manner in WT vs KO mice.

### Importance of AMY_1_R and AMY_3_R for cagrilintide-induced cFos activation in the AP, NTS, and LPBN

**Fig. 4:**
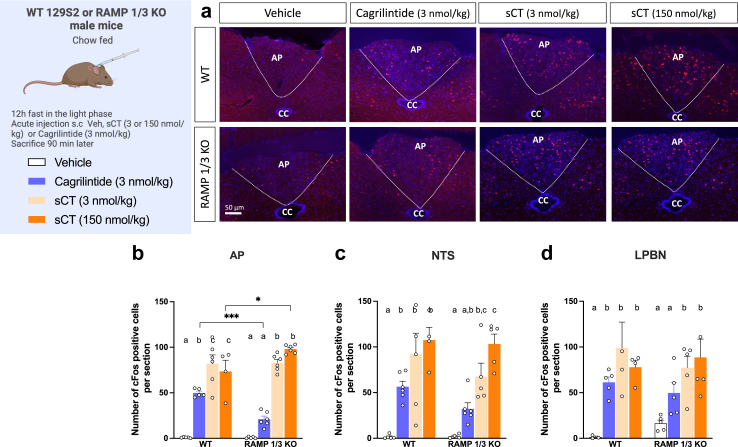
Activation of neurons of the area postrema (AP), nucleus of the solitarii tract (NTS) and lateral parabrachial nucleus (LPBN) in chow diet fed RAMP1/3 KO and WT male mice acutely treated with vehicle, sCT or cagrilintide. **a:** representative immunostainings (20× magnification) of the AP region showing cFos (red) response following vehicle and cagrilintide systemic treatment in RAMP1/3 KO mice; **b:** number of cFos positive neurons following the injection of vehicle, sCT (3 or 150 nmol/kg) or cagrilintide (3 nmol/kg, IP) in the AP; **c:** number of cFos positive neurons in the NTS; **d:** number of cFos positive neurons in the LPBN. Data are represented as mean ± SEM, *n* = 5 per group. Statistics: data were analysed by two-way ANOVA (factors: treatment and genotype) followed by Tukey's post-hoc comparison; ∗*P* < 0.05, ∗∗*P* < 0.01, ∗∗∗*P* < 0.001, ∗∗∗∗*P* < 0.0001. Whitin each genotype, ^a, b, c^ groups with different superscripts are different from each other at *P* < 0.05 or more.

In an acute study involving WT and RAMP1/3 KO chow-fed male mice, neuronal activity was examined following the administration of vehicle, cagrilintide (3 nmol/kg), and two different doses of sCT (3 and 150 nmol/kg) (Fig. 4a). The areas of interest included the AP, NTS and LPBN. Compared to their respective vehicle-treated group, cagrilintide and sCT-injected WT mice exhibited higher cFos signals in the AP, NTS, and LPBN (*P* < 0.05. two-way ANOVA**;** Fig. 4b, c, d). In RAMP 1/3 KO mice, cagrilintide did not significantly activate AP, NTS and LPBN neurons as compared to vehicle-injected mice while sCT was able to do so (Fig. 4b, c, d). When comparing cagrilintide and sCT at the doses of 3 nmol/kg in WT and KO mice, cagrilintide significantly activated fewer neurons in the AP (−75%; *P* < 0.001. two-way ANOVA; Fig. 4b) and in the LPBN (−36%; *P* < 0.001. two-way ANOVA; Fig. 4d) suggesting potential differences in their mechanisms of pathway activation. In the AP, cagrilintide activated 57% less neurons in KO vs WT mice (*P* < 0.001. two-way ANOVA; Fig. 4b) while in the NTS and LPBN, no difference between the WT and KO mice was found upon cagrilintide administration (Fig. 4c and d). The lowest sCT dose was however not tested *in vivo* precluding us to perform any correlation between hindbrain cFos activation and food intake response. Furthermore, no major difference in the activation pattern of sCT was observed between WT and RAMP 1/3 KO mice. The two doses of sCT induced similar numbers of cFos positive neurons in the two genotypes and across all brain areas, suggesting that 3 nmol/kg is sufficient to reach the maximum activation, even in the absence of RAMP 1 and 3. This activation may thus be mediated by the remaining AMY_2_R in the KO mice.

### AMY_1_R and AMY_3_R are both essential for eliciting a full cFos response in the AP following cagrilintide administration

**Fig. 5:**
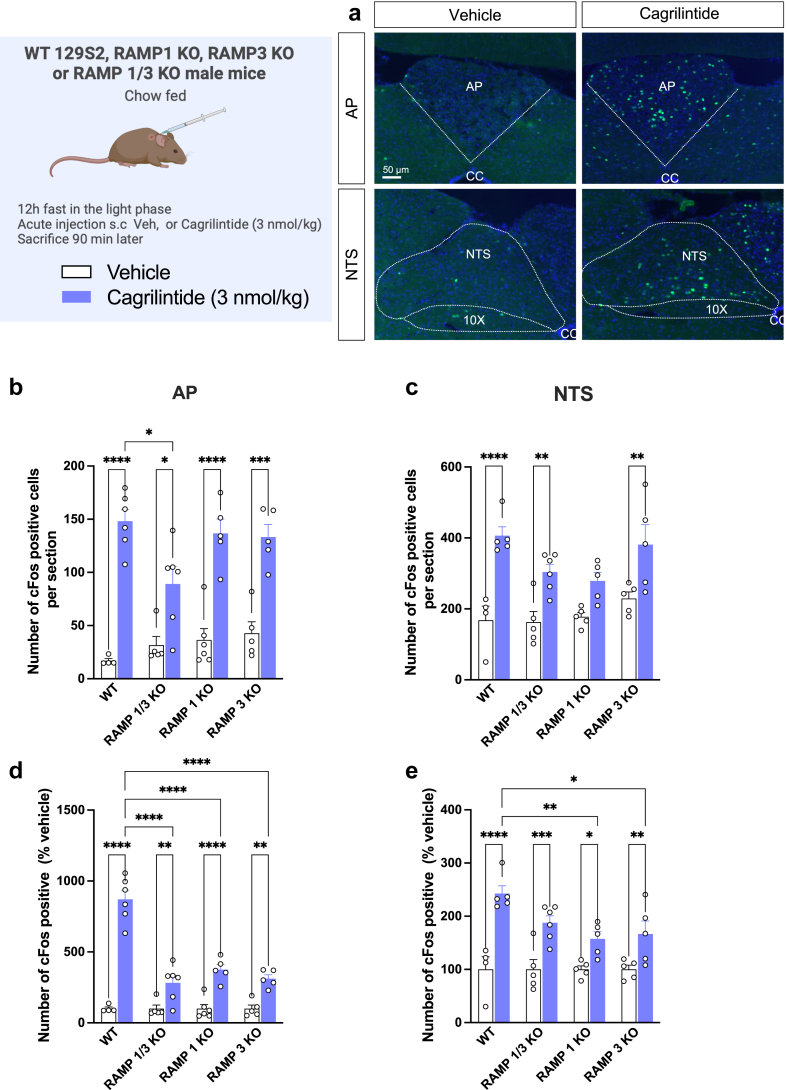
**Neuronal activation in the area postrema (AP) and nucleus of the solitarii tract (NTS) after acute injection of cagrilintide or sCT in chow fed WT, RAMP1 KO, RAMP3 KO and RAMP1/3 KO mice. a**: representative immunostainings (20× magnification) of the AP/NTS region showing cFos response (green) following vehicle and cagrilintide systemic treatment in RAMP1/3 KO mice; **b**: number of cFos positive neurons following the injection of cagrilintide (3 nmol/kg, IP) in the AP as compared to vehicle-treated mice; **c**: number of cFos positive neurons in the NTS. **d**: number of cFos positive AP neurons expressed as percent vehicle for each genotype. **e**: number of cFos positive NTS neurons expressed as percent vehicle for each genotype. Data are represented as mean ± SEM, *n* = 5/6 per group. Statistics: data were analysed by two-way ANOVA (factors: treatment and genotype) followed by Tukey's post-hoc comparison; ∗*P* < 0.05, ∗∗*P* < 0.01, ∗∗∗*P* < 0.001, ∗∗∗∗*P* < 0.0001.

In the second cohort of male mice fed a chow diet, neuronal activation within the AP and NTS was examined in WT, RAMP1/3 KO, RAMP1 KO and RAMP3 KO mice to assess the role of individual RAMP on cagrilintide-induced cFos response. Remarkably, when compared to the vehicle groups, cagrilintide demonstrated a notable and statistically significant activation of neurons in the hindbrain (Fig. 5) but the number of cagrilintide-activated neurons was decreased by 40% in the RAMP1/3 KO compared to WT-injected mice in the AP (*P* < 0.05. two-way ANOVA; Fig. 5b), similar to what we observed the first cohort (Fig. 4b). As the number of cFos positive cells in the vehicle groups varied across genotype, data were normalised to the vehicle group. In all KO mice, cagrilintide-induced cFos was significantly decreased compared to WT mice in AP and NTS regions (*P* < 0.05. two-way ANOVA; Fig. 5d and e) confirming that AMY_1_R and AMY_3_R are necessary for the neuronal activation upon cagrilintide administration.

### Gene expression in AP/NTS and ARC after 3 weeks of treatment

**Fig. 6:**
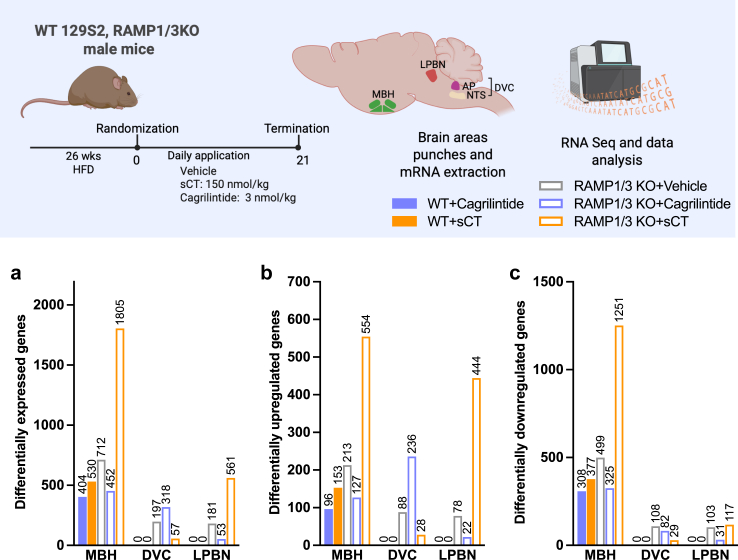
**Number of differentially expressed gene in the DVC, LPBN and MBH nuclei in WT and RAMP1/3 KO mice treated for 21 days with vehicle, sCT and cagrilintide. a:** Total number of differentially expressed genes (DEGs); **b**: Upregulated DEGs; **c**: Downregulated DEGs. Data are expressed as compared to WT vehicle-treated mice. The number of DEG is indicated above each bar. *n* = 6 per group.

Following the 21 days of treatment, the DVC, LPBN and MBH were punched, mRNA extracted to identify global patterns of differential gene expression using bulk RNA-seq. First, datasets were normalised, filtered and compared to WT vehicle-treated mice for each area of interest. The gene expression profiles of WT and RAMP1/3 KO mice across the different treatment groups in the MBH, DVC, and LPBN in comparison with the vehicle-treated WT group revealed distinct patterns. Firstly, it became evident that gene expression changes were more pronounced in the MBH, especially in contrast to the other brain regions (Fig. 6a–c). Focussing specifically on the MBH, RAMP 1/3 KO mice treated with sCT demonstrated the most significant genetic alterations involving over 1700 genes. Among these genes, more than 500 were upregulated (Fig. 6b), and over 1200 were downregulated (Fig. 6c). In contrast, the WT groups exhibited a milder alteration in gene expression, involving fewer than 500 genes, with a tendency towards downregulation. Upon comparing the different treatment across the genotype, we observed that WT mice exhibit no expression changes upon cagrilintide or sCT treatment in the DVC and LPBN (Fig. 6b and c). This absence of DEG in cagrilintide and sCT-treated WT mice prevented us to perform a complete analysis of the datasets and compare differences in DEGs between treatment and genotype in the DVC and LPBN.

**Fig. 7:**
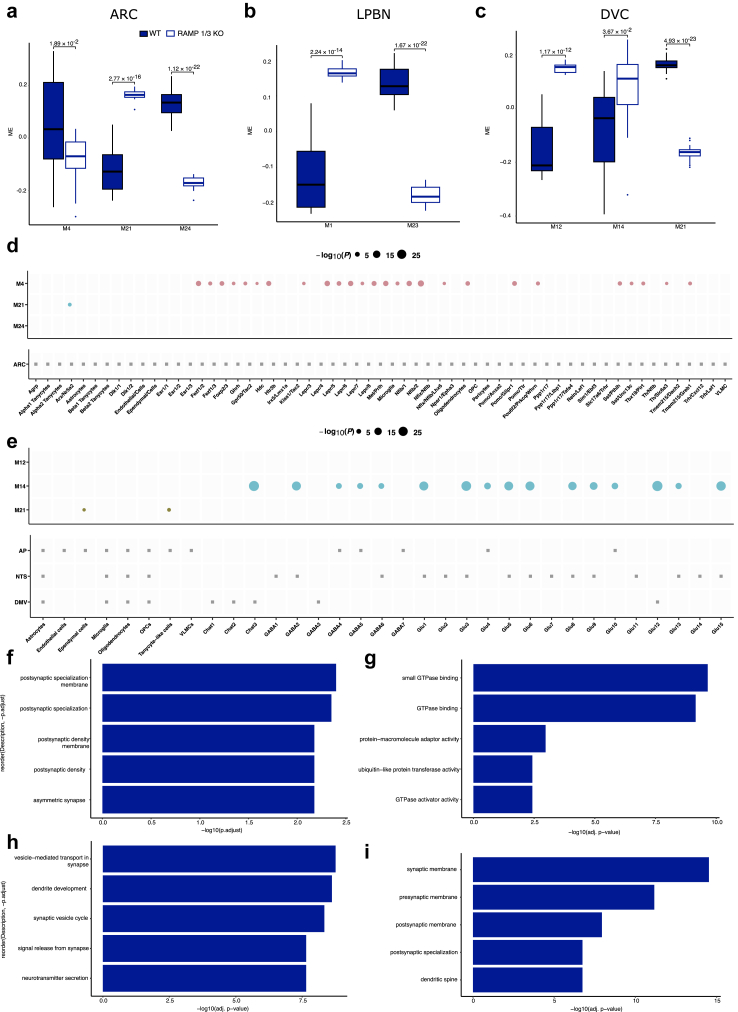
**Transcriptional changes in WT and RAMP 1/3 KO mice. (a**–**c)** Bulk RNA-seq data were clustered into modules of co-regulated genes. **a:** Mediobasal hypothalamus, MBH; **b:** Lateral parabrachial nucleus, LPBN; **c:** Dorsal vagal complex, DVC. Data represent the median, and first and third quartiles and whiskers with minimum and maximum values represent 1.5 times the interquartile ranges. *P* < 0.05 are specified; WT mice (*n* = 24 per group) vs RAMP 1/3 KO mice (*n* = 24 per group) mice; logistic regression with Bonferroni-adjusted likelihood-ratio test. (**d-e**): Cell population enrichment of module genes in the mediobasal hypothalamus (**d**-MBH) and the dorsal vagal complex (**e**-DVC). Dot size indicates the significance level of overlap between module genes and cell population marker genes (*ES*_μ_ > 0). Bonferroni-adjusted one-tailed Fisher's exact test. (**f-i**): Top five most enriched GO terms in the MBH (**f**) and top five most enriched GO terms in each module in the DVC for molecular function (**g**), biological process (**h**), and cellular component (**i**). Bonferroni-adjusted g: Profiler *P* value.

To better determine the transcriptional changes in WT vs KO mice (the treatments were not taken into account), a weighted gene co-expression network analysis (WGCNA; a computational approach to identify sets of co-regulated genes associated with treatment outcomes) on bulk RNA-seq data was applied where groups of genes that move in the same direction between conditions are packed into modules. This analysis revealed a genotype effect rather than a treatment effect (Fig. 7). The analysis attempts to find patterns in gene expression that change in unison and possibly reveal some underlying biology. In the MBH, 3 gene modules (M4, M21 and M24) were identified with M21 being positively associated with the genotype while M4 and M24 were negatively associated (Fig. 7a). In the DVC, modules M12 and M14 contained genes that were positively associated with the genotype while M21 was negatively associated (Fig. 7b). In the LPBN, two gene modules were identified, M1 being positively associated while M23 was negatively associated with the genotype (Fig. 7c). To assess whether these genotype-associated modules represented cell population-specific processes, we next determined whether module genes selectively overlapped with cell population marker genes (Fig. 7d and e). Each dot represents a significant overlap between the module genes with genes that those specific cell types are expressing. In the MBH and DVC, modules M4 and M14 respectively, expressed genes that are broadly impacting the neurons. GO terms analysis suggested that the MBH and DVC expressed genes that are broadly impacting neuronal gene expression pointing towards synaptic functionality (synaptic membrane, vesicle-mediated transport in synapse, neurotransmitter secretion) (Fig. 7f, h, i) as well as molecular function (GTPase activity) (Fig. 7g).

## Discussion

The primary objectives of this study were to investigate the role of AMY_1_R and AMY_3_R in mediating the metabolic effects of amylin and calcitonin receptor agonists, specifically cagrilintide and sCT, and to explore potential differences in their mechanisms of action at the *in vivo* and *ex vivo* levels.

The subchronic treatment of WT and RAMP1/3 KO mice with cagrilintide and sCT revealed distinct effects. While cagrilintide elicited an anorectic response in WT mice that persisted only for the first day of treatment, no such effect was observed in RAMP1/3 KO mice. Despite both cagrilintide groups not showing a significant reduction in food intake over the three-week period, WT mice exhibited a sustained reduction in body weight until the end of the three-week treatment period. The weight loss induced by cagrilintide in WT mice resulted from a proportional reduction in fat mass while maintaining lean mass, a response not observed in RAMP1/3 KO mice treated with cagrilintide, indicating a RAMP1/3-dependent mechanism. A previous metabolic assessment of RAMP KO mice indicated a specific contribution of RAMPs to eating and metabolic behaviours, with AMY_1_R identified as a key factor in regulating fat metabolism.^37^ Notably, RAMP1 KO mice exhibited greater fat accumulation compared to WT and RAMP3 KO mice when fed a high-fat diet (HFD).^37^ Thus, we may here hypothesise that at least in mice, the predominant impact of cagrilintide through AMY_1_R contributed to the observed reduction in fat mass in WT mice compared to the KO mice.

An additional factor contributing to the persistent weight loss triggered by cagrilintide, without a concomitant anorectic response, could be attributed to its impact on energy expenditure. Recent investigations involving diet-induced obese (DIO) rats treated with CagriSema, a combination of cagrilintide and semaglutide, did not elicit a reduction in energy expenditure despite a strong body weight loss. Besides the importance of preserving energy expenditure levels, the reduction of fat mass by simultaneously maintaining the lean mass when treated with cagrilintide is crucial for effective obesity management. The extent to which cagrilintide-induced fat mass reduction impacts body composition and metabolic profiles, along with its implications for long-term weight maintenance, warrants further investigation.^43^

In stark contrast to cagrilintide, sCT exhibited divergent effects on body weight in WT and KO mice. Specifically, sCT caused weight gain in WT mice while inducing initial weight loss followed by partial weight regain in HFD-fed RAMP1/3 KO mice. Similarly, in a previous study with HFD-fed WT and RAMP1/3 KO mice, we also observed that sCT-treated WT mice gained weight, whereas RAMP1/3 KO mice experienced weight loss, followed by minimal weight regain by the end of the treatment period.^42^ In the current study, sCT initially promoted weight loss in RAMP1/3 KO mice, but by the end of the study, these mice exhibited higher fat mass and lower lean mass compared to their WT counterparts. These changes in body composition were accompanied by elevated plasma insulin levels in sCT-treated KO mice, an effect not observed with cagrilintide possibly indicating a prediabetic state. These findings suggest that the calcitonin receptor agonist sCT may engage distinct metabolic pathways in mice by independently utilising either AMYRs or CTR. Specifically, the presence of AMY_1_R and AMY_3_R hinders the effect of sCT on body weight loss but conversely appears to be crucial for food intake control, glucose homoeostasis and nutrient partitioning. Consistent with this, the ablation of CTR in AP neurons of mice blunted the anorectic effects of sCT (10 μg/kg, equivalent to 3 nmol/kg)^44^ and the ablation of CTR in NTS neurons in mice abrogated the long-term suppression of food intake upon sCT administration.^45^

Concurrently, there is an ongoing exploration of another class of amylin analogues with affinity for both the AMYRs and the CTR for both preclinical and clinical applications, KBP-336. This analogue is based on sCT, thereby exhibiting a distinct receptor activation profile compared to cagrilintide. Cagrilintide and KBP-336 were tested in HFD-fed WT and Zucker diabetic fatty rats. However, although KBP-336 is a sCT-based analogue, the treated rats did not exhibit weight gain in contrast to sCT in our studies with mice; instead, they lost weight, with the highest dose of KBP-336 being the most effective.^46^^,^^47^ Indeed, it seems that chronically administered analogues such as sCT produced differential effects depending on the species. In rats, sCT induces weight loss as in humans or other animal models while in mice, weight gain is observed.^42^^,^^48^ To further explore interspecies differences, cagrilintide was administered acutely and subchronically in mice and rats, followed by single-nucleus RNA sequencing of the DVC to generate a cross-species and spatial atlas of this region. Following subchronic cagrilintide treatment, rats, but not mice, show alterations in *Calcr/Prlh*-expressing cells suggesting a role for this cell population in the mechanism of weight loss (PREPRINT^49^). Altogether this emphasises the need for further research to clarify the receptor-specific mechanisms of sCT and to explore potential species-specific differences in its interactions with AMYRs, particularly among rats, mice, and humans.

The assessment of neuronal activation using cFos as a surrogate marker in response to an acute administration of cagrilintide and sCT in chow-fed mice revealed that both cagrilintide and sCT produced a significantly higher cFos signal compared to vehicle-treated mice, showing similar activation patterns in WT mice AP, NTS and LPBN brain areas. However, in the AP, cagrilintide activated fewer neurons compared to sCT, suggesting potential differences in the activation mechanisms. RAMP1/3 KO mice exhibited a decreased cFos signal in response to cagrilintide compared to WT cagrilintide-treated mice. This was also true for RAMP1 KO and RAMP3 KO mice who exhibited a decrease in cagrilintide-induced cFos positive neurons compared to WT mice. The CTR protein is still present in the AP of RAMP1/3 KO mice but in a lesser amount than in WT mice^37^ suggesting that complete AMY_1_R and AMY_3_R are necessary for a full cFos response upon cagrilintide administration. The dose of 150 nmol/kg sCT was chosen to compare the effect of sCT and cagrilintide *in vivo* as this dose of sCT shows the same pharmacokinetic response as the 3 nmol/kg of cagrilintide. Notably, the two doses of sCT activated a similar number of neurons in the three brain areas suggesting that a dose of 3 nmol/kg is sufficient to maximise the neuronal response. Due to the stronger sCT induced signal in the AP of WT mice, at a dose that caused the same anorectic and body weight effects as cagrilintide, the greater neuronal activation may result from a higher residence time at the CTR.^20^^,^^28^ Even though cagrilintide activated a lesser number of neurons than sCT in the AP, NTS and LPBN, it resulted in similar metabolic effects as sCT on day 1 of injection suggesting that the metabolic effects cannot be predicted by quantitative activation. On the other hand, despite sCT-induced neuronal activation in the AP of RAMP 1/3 KO HFD-fed mice, it did not result in an anorectic effect on day 1 of treatment suggesting the necessity of AMY_1_R and AMY_3_R to induce anorexia and that this circuitry is independent of signalling at the core CTR receptor. We also hypothesise that this may result from activation of the hypothalamic circuitry. As the effect of cagrilintide on neuronal activation of ARC neurons was not assessed in this study, we cannot speculate on the implication of AgRP or POMC neurons in mediating anorexia or body weight loss.^16^, ^17^, ^18^ Nevertheless, we have previously shown that CTR signalling in POMC neurons is necessary for the maintenance of energy balance though the control of energy expenditure.^16^ Finally, as neuronal activation and food intake were not performed under the same diet condition or in the same animal, it is difficult to correlate the effect of sCT on eating and the neuronal activation.

Cagrilintide exhibits activity on both AMYRs and the CTR, similar to sCT. However, its role as a long-acting amylin analogue demonstrates distinct actions at AMY_1_R and AMY_3_R, unlike sCT, which may cause receptor downregulation and counteradaptation. Pharmacodynamics differences and distinct activation patterns between sCT and multiple amylin receptors agonists were highlighted by Fletcher et al.^28^ and our own functional cellular assay using mice CTR and AMY_3_R. In a cellular assay, cagrilintide was confirmed to be a non-selective agonist of the CTR and AMYRs across multiple assays of proximal and distal receptor signalling and receptor trafficking but only showed subtle difference in the kinetic response for activation of select G proteins as compared to sCT. Among all agonists, sCT was the least selective peptide towards the CTR, AMY_1_R or AMY_3_R.^28^ Cryogenic electron microscopy showed that sCT activated AMYRs via distinct mechanism compared to the AMYR agonist pramlintide.^50^ In addition, the binding of amylin to AMYRs resulted in a different conformation allowing the stabilisation of the receptor differently to that of sCT^29^; this difference in receptor binding may lead to a differential recruitment of downstream neuronal pathway in rodent models and *in vivo* body weight lowering effect.^40^^,^^42^ Cagrilintide also exhibited a rapid dissociation with a residence time of 3–6 min^28^ that is comparable for all AMYRs whereas sCT showed slow rates of dissociation from all receptors (45–60 min) confirming previous studies performed *in vivo* and *in vitro*.^51^, ^52^, ^53^ This effect was associated with a longer duration of action of the peptide in cAMP production with a sustained response for sCT while cagrilintide’ cAMP activation returned to baseline within a few hours. Our studies in mice indicate that the longer residency time of sCT may rather be detrimental and may lead to weight gain rather than weight loss, potentially via counter regulatory responses. Davalintide, a sCT based compound, binds irreversibly to AMYRs but does not show greater benefit compared to pramlintide.^28^^,^^54^ Nevertheless, our studies and others clearly indicate the clinical potential in the activation of CTR and AMYRs which are supported by a greater body weight loss in rodent models.^42^^,^^46^ The pharmacological profile of cagrilintide support this fact of improved obesity therapeutic peptide, attributed to its long availability due to a prolonged half-life while benefiting from a shorter residency time. We acknowledge that although cagrilintide and sCT differ significantly in both structure and kinetics, and that the primary goal was to use the sCT molecule as a control. Despite having a shorter half-life compared to cagrilintide, the slower off-rate of sCT makes it a suitable one to function as its control.

To potentially explain the difference of action between cagrilintide and sCT, we compared the differentially gene expression levels following the three-week treatment in various brain regions, including MBH, DVC, and LPBN. Notably, no treatment effect was detected at the end of the three-week study preventing us to fully assess the treatments on gene expression. We suggest that the timing of the assessment, i.e. at the end of the three-week treatment may have resulted in this absence of signal. Indeed, at the end of the study, the sCT or cagrilintide-treated mice were not in active weight loss anymore. Nevertheless, bulk-RNAseq allowed us to look deeper into the function of RAMP1 and RAMP3. Module gene analysis and GO term mainly pointed to a role of RAMP1 and RAMP3 in impacting neuronal and synaptic function but as these receptors are widely expressed, their role is not limited to a specific type of neurons. The DVC contains a wide variety of neuronal populations^39^^,^^55^ all playing a crucial role in the control of eating. The AMYRs are located in the AP on DBH positive neurons^15^ and *Calcr* neuronal populations do express other receptors such as the GLP-1R or GFRAL.^39^ However, *RAMPs* can be expressed on other cell types than *Calcr* positive neurons and can be associated to other receptor subunits.^22^^,^^56^^,^^57^ The GO term analysis suggests that *RAMPs* may be important for synaptic function, neurotransmitter secretion, vesicle trafficking and membrane composition. Notably, RAMPs have been described to participate in receptor trafficking, i.e. translocation from the endoplasmic reticulum to the Golgi, internalisation and recycling of the receptor.^58^, ^59^, ^60^, ^61^ The impact on many neuronal subtypes that are not CTR-expressing supports the notion that RAMPs interact with more GPCRs than just CTR-related family.^13^^,^^56^ A limitation of our gene analysis study is the lack of weight-matched groups, making it difficult to dissociate the effects of the drugs from those of weight loss. More significant results might have been obtained if bulk NGS analysis had been performed on brain samples collected during the active phase of weight loss, i.e. in the first days of treatment. In our study, brain samples were collected at the end of the 21-day treatment, by which point gene expression and metabolic pathways may have already adapted.

In conclusion, this series of studies demonstrates that cagrilintide lowers body weight through a mechanism that depends on the presence of AMY_1_R and AMY_3_R in WT mice. Despite similarities in target receptor populations, treatment with sCT or cagrilintide demonstrated opposing effects on body weight and body composition which may be due to *in vitro* receptor dynamics, such as residence time or signalling properties of the two molecules at AMY_1_R and AMY_3_R.^28^ However, these two aspects need further investigation as we could not identify expressional changes in specific genes of the MBH and/or DVC following treatment. More research is needed at the receptor and genomic level to gain a deeper understanding of the mechanisms underlying the effects of amylin and amylin analogues on energy metabolism, particularly in relation to other pathways that may be affected by the compounds, including those influenced by RAMP1 and RAMP3 knockout. Nevertheless, these studies confirm the *in vivo* specificity of cagrilintide to the amylin receptor, and that cagrilintide requires the AMY_1_R and AMY_3_R to induce weight loss.

## Contributors

**Alexandra Oliveira Carvas:** Investigation, data analysis and interpretation, writing–original draft. **Andrea S. Leuthard:** Investigation, statistical analysis. **Patricia Kulka:** Investigation. **Greta Lommi**: Investigation. **Shad Hassan:** Data analysis and interpretation, writing–original draft. **Bernd Coester:** Data analysis and interpretation, writing–original draft. **Sofia Lundh:** Conceptualisation, editing. **Tune Pers**: Ressources. **Anna Secher:** Editing. **Kirsten Raun:** Conceptualisation, Editing. **Thomas A. Lutz:** Conceptualisation, editing, resources, funding acquisition. **Christelle Le Foll:** Conceptualisation, methodology, investigation, statistical analysis, writing–original and final draft, editing, guarantor of this work and, as such, had full access to all the data in the study and take responsibility for the integrity of the data and the accuracy of the data analysis. All authors have read and approved the final version of the manuscript.

## Data sharing statement

All raw and processed RNA-seq data is available through the NCBI GEO database. The data is available at https://www.ncbi.nlm.nih.gov/geo/query/acc.cgi?acc=GSE296488.

Full datasets are available upon request.

## Declaration of interests

K.R., A.S., S.L. are full-time employees of Novo Nordisk and hold minor share portions as part of their employment. C.L.F declare no financial and no non-financial conflict of interest.
