# Supplementary material for: Cagrilintide lowers bodyweight through brain amylin receptors 1 and 3
Source: eBioMedicine. 2025 Jul 3;118:105836. doi: 10.1016/j.ebiom.2025.105836 (PMC12270663; doi:10.1016/j.ebiom.2025.105836)
Supplement: Supplementary Figures [file mmc1.docx]

**Cagrilintide lowers bodyweight through brain amylin receptors 1 and 3**

**Supplementary data**

**Suppl. Figure 1: WT male were fed chow diet and acutely injected with increasing doses of cagrilintide (3, 30, 300 nmol/kg) or vehicle**. **a**: 24h hourly cumulative food intake (g); **b**: 24h body weight change (g). Data are represented as mean ± SEM, *n*= 7/8 per group. Statistics: data were analyzed by two-way ANOVA (factors: treatment and time) followed by Tukey’s post-hoc comparison or by one-way ANOVA; * *P*<0.05, ** *P*<0.01, *** *P*<0.001, **** *P*<0.0001.

**Suppl. Figure 2: WT and RAMP KO male mice were fed 60% high fat diet (HFD) from 3 week-old until 22 week-old, they were then switched to 45% HFD for the following 2 weeks and during the 3 week treatment period**. **a**: Body weight; **b**: cumulative body weight gain; **c**: blood glucose measured at the end of the 60% HFD period and before the 3 week treatment. Data are represented as mean ± SEM, *n*= 24 per group. Statistics: data were analyzed by two-way ANOVA (factors: Time and genotype (Gen)) followed by Tukey’s post-hoc comparison; * *P*<0.05, **** *P*<0.0001.

**Detailled statistical analysis of all graphs**

**Figure 1a: daily food intake**

Statistical test: Two-way Anova. Factors: group and time.

Daily food intake is independent from the previous day so no repeated measure was performed.

| **Two-way ANOVA** | Ordinary |  |  |  |  |
| --- | --- | --- | --- | --- | --- |
| Alpha | 0.05 |  |  |  |  |
|  |  |  |  |  |  |
| Source of Variation | % of total variation | P value | P value summary | Significant? |  |
| Interaction | 24.51 | <0.0001 | **** | Yes |  |
| Time | 12.02 | <0.0001 | **** | Yes |  |
| Group | 1.700 | 0.0014 | ** | Yes |  |
|  |  |  |  |  |  |
| ANOVA table | SS (Type III) | DF | MS | F (DFn, DFd) | P value |
| Interaction | 45.02 | 63 | 0.7147 | F (63, 572) = 3.593 | P<0.0001 |
| Time | 22.08 | 21 | 1.051 | F (21, 572) = 5.287 | P<0.0001 |
| Group | 3.123 | 3 | 1.041 | F (3, 572) = 5.234 | P=0.0014 |
| Residual | 113.8 | 572 | 0.1989 |  |  |

Post Hoc multiple comparison

| **Tukey's multiple comparisons test** | **Predicted (LS) mean dif.** | **95.00% CI of diff.** | **Below threshold?** | **Summary** | **Adjusted P Value** |
| --- | --- | --- | --- | --- | --- |
|  |  |  |  |  |  |
| 1 |  |  |  |  |  |
| Vehicle vs. Cagrilintide (0.3 nmol/kg) | 0.6857 | 0.09102 to 1.280 | Yes | * | 0.0163 |
| Vehicle vs. Cagrilintide (3 nmol/kg) | 2.198 | 1.604 to 2.793 | Yes | **** | <0.0001 |
| Vehicle vs. Cagrilintide (30 nmol/kg) | 2.243 | 1.629 to 2.857 | Yes | **** | <0.0001 |
| Cagrilintide (0.3 nmol/kg) vs. Cagrilintide (3 nmol/kg) | 1.513 | 0.9380 to 2.087 | Yes | **** | <0.0001 |
| Cagrilintide (0.3 nmol/kg) vs. Cagrilintide (30 nmol/kg) | 1.557 | 0.9625 to 2.152 | Yes | **** | <0.0001 |
| Cagrilintide (3 nmol/kg) vs. Cagrilintide (30 nmol/kg) | 0.04464 | -0.5500 to 0.6393 | No | ns | 0.9974 |
|  |  |  |  |  |  |
| 2 |  |  |  |  |  |
| Vehicle vs. Cagrilintide (0.3 nmol/kg) | 0.3732 | -0.2215 to 0.9679 | No | ns | 0.3698 |
| Vehicle vs. Cagrilintide (3 nmol/kg) | 1.336 | 0.7410 to 1.930 | Yes | **** | <0.0001 |
| Vehicle vs. Cagrilintide (30 nmol/kg) | 1.229 | 0.6144 to 1.843 | Yes | **** | <0.0001 |
| Cagrilintide (0.3 nmol/kg) vs. Cagrilintide (3 nmol/kg) | 0.9625 | 0.3880 to 1.537 | Yes | *** | 0.0001 |
| Cagrilintide (0.3 nmol/kg) vs. Cagrilintide (30 nmol/kg) | 0.8554 | 0.2607 to 1.450 | Yes | ** | 0.0013 |
| Cagrilintide (3 nmol/kg) vs. Cagrilintide (30 nmol/kg) | -0.1071 | -0.7018 to 0.4875 | No | ns | 0.9668 |
|  |  |  |  |  |  |
| 3 |  |  |  |  |  |
| Vehicle vs. Cagrilintide (0.3 nmol/kg) | 0.2714 | -0.3233 to 0.8661 | No | ns | 0.6423 |
| Vehicle vs. Cagrilintide (3 nmol/kg) | 0.6214 | 0.02674 to 1.216 | Yes | * | 0.0366 |
| Vehicle vs. Cagrilintide (30 nmol/kg) | 0.7857 | 0.1715 to 1.400 | Yes | ** | 0.0057 |
| Cagrilintide (0.3 nmol/kg) vs. Cagrilintide (3 nmol/kg) | 0.3500 | -0.2245 to 0.9245 | No | ns | 0.3969 |
| Cagrilintide (0.3 nmol/kg) vs. Cagrilintide (30 nmol/kg) | 0.5143 | -0.08041 to 1.109 | No | ns | 0.1169 |
| Cagrilintide (3 nmol/kg) vs. Cagrilintide (30 nmol/kg) | 0.1643 | -0.4304 to 0.7590 | No | ns | 0.8924 |
|  |  |  |  |  |  |
| 4 |  |  |  |  |  |
| Vehicle vs. Cagrilintide (0.3 nmol/kg) | 0.01786 | -0.5768 to 0.6125 | No | ns | 0.9998 |
| Vehicle vs. Cagrilintide (3 nmol/kg) | 0.5179 | -0.07683 to 1.113 | No | ns | 0.1129 |
| Vehicle vs. Cagrilintide (30 nmol/kg) | 0.4000 | -0.2142 to 1.014 | No | ns | 0.3363 |
| Cagrilintide (0.3 nmol/kg) vs. Cagrilintide (3 nmol/kg) | 0.5000 | -0.07453 to 1.075 | No | ns | 0.1132 |
| Cagrilintide (0.3 nmol/kg) vs. Cagrilintide (30 nmol/kg) | 0.3821 | -0.2125 to 0.9768 | No | ns | 0.3483 |
| Cagrilintide (3 nmol/kg) vs. Cagrilintide (30 nmol/kg) | -0.1179 | -0.7125 to 0.4768 | No | ns | 0.9566 |
|  |  |  |  |  |  |
| 5 |  |  |  |  |  |
| Vehicle vs. Cagrilintide (0.3 nmol/kg) | -0.08393 | -0.6786 to 0.5108 | No | ns | 0.9835 |
| Vehicle vs. Cagrilintide (3 nmol/kg) | 0.2661 | -0.3286 to 0.8608 | No | ns | 0.6569 |
| Vehicle vs. Cagrilintide (30 nmol/kg) | -0.08571 | -0.6999 to 0.5285 | No | ns | 0.9841 |
| Cagrilintide (0.3 nmol/kg) vs. Cagrilintide (3 nmol/kg) | 0.3500 | -0.2245 to 0.9245 | No | ns | 0.3969 |
| Cagrilintide (0.3 nmol/kg) vs. Cagrilintide (30 nmol/kg) | -0.001786 | -0.5965 to 0.5929 | No | ns | >0.9999 |
| Cagrilintide (3 nmol/kg) vs. Cagrilintide (30 nmol/kg) | -0.3518 | -0.9465 to 0.2429 | No | ns | 0.4236 |
|  |  |  |  |  |  |
| 6 |  |  |  |  |  |
| Vehicle vs. Cagrilintide (0.3 nmol/kg) | 0.06429 | -0.5304 to 0.6590 | No | ns | 0.9925 |
| Vehicle vs. Cagrilintide (3 nmol/kg) | 0.2518 | -0.3429 to 0.8465 | No | ns | 0.6953 |
| Vehicle vs. Cagrilintide (30 nmol/kg) | -0.05714 | -0.6713 to 0.5571 | No | ns | 0.9952 |
| Cagrilintide (0.3 nmol/kg) vs. Cagrilintide (3 nmol/kg) | 0.1875 | -0.3870 to 0.7620 | No | ns | 0.8349 |
| Cagrilintide (0.3 nmol/kg) vs. Cagrilintide (30 nmol/kg) | -0.1214 | -0.7161 to 0.4733 | No | ns | 0.9528 |
| Cagrilintide (3 nmol/kg) vs. Cagrilintide (30 nmol/kg) | -0.3089 | -0.9036 to 0.2858 | No | ns | 0.5387 |
|  |  |  |  |  |  |
| 7 |  |  |  |  |  |
| Vehicle vs. Cagrilintide (0.3 nmol/kg) | -0.1554 | -0.7500 to 0.4393 | No | ns | 0.9072 |
| Vehicle vs. Cagrilintide (3 nmol/kg) | -0.09286 | -0.6875 to 0.5018 | No | ns | 0.9780 |
| Vehicle vs. Cagrilintide (30 nmol/kg) | -0.2000 | -0.8142 to 0.4142 | No | ns | 0.8358 |
| Cagrilintide (0.3 nmol/kg) vs. Cagrilintide (3 nmol/kg) | 0.06250 | -0.5120 to 0.6370 | No | ns | 0.9923 |
| Cagrilintide (0.3 nmol/kg) vs. Cagrilintide (30 nmol/kg) | -0.04464 | -0.6393 to 0.5500 | No | ns | 0.9974 |
| Cagrilintide (3 nmol/kg) vs. Cagrilintide (30 nmol/kg) | -0.1071 | -0.7018 to 0.4875 | No | ns | 0.9668 |
|  |  |  |  |  |  |
| 8 |  |  |  |  |  |
| Vehicle vs. Cagrilintide (0.3 nmol/kg) | -0.2196 | -0.8143 to 0.3750 | No | ns | 0.7769 |
| Vehicle vs. Cagrilintide (3 nmol/kg) | -0.007143 | -0.6018 to 0.5875 | No | ns | >0.9999 |
| Vehicle vs. Cagrilintide (30 nmol/kg) | -0.4286 | -1.043 to 0.1856 | No | ns | 0.2754 |
| Cagrilintide (0.3 nmol/kg) vs. Cagrilintide (3 nmol/kg) | 0.2125 | -0.3620 to 0.7870 | No | ns | 0.7761 |
| Cagrilintide (0.3 nmol/kg) vs. Cagrilintide (30 nmol/kg) | -0.2089 | -0.8036 to 0.3858 | No | ns | 0.8021 |
| Cagrilintide (3 nmol/kg) vs. Cagrilintide (30 nmol/kg) | -0.4214 | -1.016 to 0.1733 | No | ns | 0.2622 |
|  |  |  |  |  |  |
| 9 |  |  |  |  |  |
| Vehicle vs. Cagrilintide (0.3 nmol/kg) | -0.3464 | -0.9411 to 0.2483 | No | ns | 0.4376 |
| Vehicle vs. Cagrilintide (3 nmol/kg) | -0.1839 | -0.7786 to 0.4108 | No | ns | 0.8558 |
| Vehicle vs. Cagrilintide (30 nmol/kg) | -0.4286 | -1.043 to 0.1856 | No | ns | 0.2754 |
| Cagrilintide (0.3 nmol/kg) vs. Cagrilintide (3 nmol/kg) | 0.1625 | -0.4120 to 0.7370 | No | ns | 0.8856 |
| Cagrilintide (0.3 nmol/kg) vs. Cagrilintide (30 nmol/kg) | -0.08214 | -0.6768 to 0.5125 | No | ns | 0.9845 |
| Cagrilintide (3 nmol/kg) vs. Cagrilintide (30 nmol/kg) | -0.2446 | -0.8393 to 0.3500 | No | ns | 0.7140 |
|  |  |  |  |  |  |
| 10 |  |  |  |  |  |
| Vehicle vs. Cagrilintide (0.3 nmol/kg) | -0.2536 | -0.8483 to 0.3411 | No | ns | 0.6905 |
| Vehicle vs. Cagrilintide (3 nmol/kg) | -0.3161 | -0.9108 to 0.2786 | No | ns | 0.5191 |
| Vehicle vs. Cagrilintide (30 nmol/kg) | -0.5000 | -1.114 to 0.1142 | No | ns | 0.1551 |
| Cagrilintide (0.3 nmol/kg) vs. Cagrilintide (3 nmol/kg) | -0.06250 | -0.6370 to 0.5120 | No | ns | 0.9923 |
| Cagrilintide (0.3 nmol/kg) vs. Cagrilintide (30 nmol/kg) | -0.2464 | -0.8411 to 0.3483 | No | ns | 0.7094 |
| Cagrilintide (3 nmol/kg) vs. Cagrilintide (30 nmol/kg) | -0.1839 | -0.7786 to 0.4108 | No | ns | 0.8558 |
|  |  |  |  |  |  |
| 11 |  |  |  |  |  |
| Vehicle vs. Cagrilintide (0.3 nmol/kg) | 0.06786 | -0.5268 to 0.6625 | No | ns | 0.9912 |
| Vehicle vs. Cagrilintide (3 nmol/kg) | 0.1179 | -0.4768 to 0.7125 | No | ns | 0.9566 |
| Vehicle vs. Cagrilintide (30 nmol/kg) | -0.04286 | -0.6571 to 0.5713 | No | ns | 0.9979 |
| Cagrilintide (0.3 nmol/kg) vs. Cagrilintide (3 nmol/kg) | 0.05000 | -0.5245 to 0.6245 | No | ns | 0.9960 |
| Cagrilintide (0.3 nmol/kg) vs. Cagrilintide (30 nmol/kg) | -0.1107 | -0.7054 to 0.4840 | No | ns | 0.9636 |
| Cagrilintide (3 nmol/kg) vs. Cagrilintide (30 nmol/kg) | -0.1607 | -0.7554 to 0.4340 | No | ns | 0.8985 |
|  |  |  |  |  |  |
| 12 |  |  |  |  |  |
| Vehicle vs. Cagrilintide (0.3 nmol/kg) | -0.1375 | -0.7322 to 0.4572 | No | ns | 0.9334 |
| Vehicle vs. Cagrilintide (3 nmol/kg) | -0.1125 | -0.7072 to 0.4822 | No | ns | 0.9619 |
| Vehicle vs. Cagrilintide (30 nmol/kg) | -0.3143 | -0.9285 to 0.2999 | No | ns | 0.5515 |
| Cagrilintide (0.3 nmol/kg) vs. Cagrilintide (3 nmol/kg) | 0.02500 | -0.5495 to 0.5995 | No | ns | 0.9995 |
| Cagrilintide (0.3 nmol/kg) vs. Cagrilintide (30 nmol/kg) | -0.1768 | -0.7715 to 0.4179 | No | ns | 0.8698 |
| Cagrilintide (3 nmol/kg) vs. Cagrilintide (30 nmol/kg) | -0.2018 | -0.7965 to 0.3929 | No | ns | 0.8182 |
|  |  |  |  |  |  |
| 13 |  |  |  |  |  |
| Vehicle vs. Cagrilintide (0.3 nmol/kg) | -0.03393 | -0.6286 to 0.5608 | No | ns | 0.9989 |
| Vehicle vs. Cagrilintide (3 nmol/kg) | -0.2214 | -0.8161 to 0.3733 | No | ns | 0.7726 |
| Vehicle vs. Cagrilintide (30 nmol/kg) | -0.2429 | -0.8571 to 0.3713 | No | ns | 0.7385 |
| Cagrilintide (0.3 nmol/kg) vs. Cagrilintide (3 nmol/kg) | -0.1875 | -0.7620 to 0.3870 | No | ns | 0.8349 |
| Cagrilintide (0.3 nmol/kg) vs. Cagrilintide (30 nmol/kg) | -0.2089 | -0.8036 to 0.3858 | No | ns | 0.8021 |
| Cagrilintide (3 nmol/kg) vs. Cagrilintide (30 nmol/kg) | -0.02143 | -0.6161 to 0.5733 | No | ns | 0.9997 |
|  |  |  |  |  |  |
| 14 |  |  |  |  |  |
| Vehicle vs. Cagrilintide (0.3 nmol/kg) | -0.08393 | -0.6786 to 0.5108 | No | ns | 0.9835 |
| Vehicle vs. Cagrilintide (3 nmol/kg) | -0.1089 | -0.7036 to 0.4858 | No | ns | 0.9652 |
| Vehicle vs. Cagrilintide (30 nmol/kg) | -0.01429 | -0.6285 to 0.5999 | No | ns | >0.9999 |
| Cagrilintide (0.3 nmol/kg) vs. Cagrilintide (3 nmol/kg) | -0.02500 | -0.5995 to 0.5495 | No | ns | 0.9995 |
| Cagrilintide (0.3 nmol/kg) vs. Cagrilintide (30 nmol/kg) | 0.06964 | -0.5250 to 0.6643 | No | ns | 0.9905 |
| Cagrilintide (3 nmol/kg) vs. Cagrilintide (30 nmol/kg) | 0.09464 | -0.5000 to 0.6893 | No | ns | 0.9767 |
|  |  |  |  |  |  |
| 15 |  |  |  |  |  |
| Vehicle vs. Cagrilintide (0.3 nmol/kg) | 0.01964 | -0.5750 to 0.6143 | No | ns | 0.9998 |
| Vehicle vs. Cagrilintide (3 nmol/kg) | -0.1554 | -0.7500 to 0.4393 | No | ns | 0.9072 |
| Vehicle vs. Cagrilintide (30 nmol/kg) | -0.05714 | -0.6713 to 0.5571 | No | ns | 0.9952 |
| Cagrilintide (0.3 nmol/kg) vs. Cagrilintide (3 nmol/kg) | -0.1750 | -0.7495 to 0.3995 | No | ns | 0.8614 |
| Cagrilintide (0.3 nmol/kg) vs. Cagrilintide (30 nmol/kg) | -0.07679 | -0.6715 to 0.5179 | No | ns | 0.9873 |
| Cagrilintide (3 nmol/kg) vs. Cagrilintide (30 nmol/kg) | 0.09821 | -0.4965 to 0.6929 | No | ns | 0.9741 |
|  |  |  |  |  |  |
| 16 |  |  |  |  |  |
| Vehicle vs. Cagrilintide (0.3 nmol/kg) | 0.04464 | -0.5500 to 0.6393 | No | ns | 0.9974 |
| Vehicle vs. Cagrilintide (3 nmol/kg) | 0.01964 | -0.5750 to 0.6143 | No | ns | 0.9998 |
| Vehicle vs. Cagrilintide (30 nmol/kg) | -0.1429 | -0.7571 to 0.4713 | No | ns | 0.9323 |
| Cagrilintide (0.3 nmol/kg) vs. Cagrilintide (3 nmol/kg) | -0.02500 | -0.5995 to 0.5495 | No | ns | 0.9995 |
| Cagrilintide (0.3 nmol/kg) vs. Cagrilintide (30 nmol/kg) | -0.1875 | -0.7822 to 0.4072 | No | ns | 0.8486 |
| Cagrilintide (3 nmol/kg) vs. Cagrilintide (30 nmol/kg) | -0.1625 | -0.7572 to 0.4322 | No | ns | 0.8955 |
|  |  |  |  |  |  |
| 17 |  |  |  |  |  |
| Vehicle vs. Cagrilintide (0.3 nmol/kg) | 0.1196 | -0.4750 to 0.7143 | No | ns | 0.9547 |
| Vehicle vs. Cagrilintide (3 nmol/kg) | 0.1946 | -0.4000 to 0.7893 | No | ns | 0.8337 |
| Vehicle vs. Cagrilintide (30 nmol/kg) | 0.02857 | -0.5856 to 0.6428 | No | ns | 0.9994 |
| Cagrilintide (0.3 nmol/kg) vs. Cagrilintide (3 nmol/kg) | 0.07500 | -0.4995 to 0.6495 | No | ns | 0.9869 |
| Cagrilintide (0.3 nmol/kg) vs. Cagrilintide (30 nmol/kg) | -0.09107 | -0.6858 to 0.5036 | No | ns | 0.9792 |
| Cagrilintide (3 nmol/kg) vs. Cagrilintide (30 nmol/kg) | -0.1661 | -0.7608 to 0.4286 | No | ns | 0.8893 |
|  |  |  |  |  |  |
| 18 |  |  |  |  |  |
| Vehicle vs. Cagrilintide (0.3 nmol/kg) | 0.08750 | -0.5072 to 0.6822 | No | ns | 0.9814 |
| Vehicle vs. Cagrilintide (3 nmol/kg) | 0.000 | -0.5947 to 0.5947 | No | ns | >0.9999 |
| Vehicle vs. Cagrilintide (30 nmol/kg) | -0.1571 | -0.7713 to 0.4571 | No | ns | 0.9123 |
| Cagrilintide (0.3 nmol/kg) vs. Cagrilintide (3 nmol/kg) | -0.08750 | -0.6620 to 0.4870 | No | ns | 0.9795 |
| Cagrilintide (0.3 nmol/kg) vs. Cagrilintide (30 nmol/kg) | -0.2446 | -0.8393 to 0.3500 | No | ns | 0.7140 |
| Cagrilintide (3 nmol/kg) vs. Cagrilintide (30 nmol/kg) | -0.1571 | -0.7518 to 0.4375 | No | ns | 0.9044 |
|  |  |  |  |  |  |
| 19 |  |  |  |  |  |
| Vehicle vs. Cagrilintide (0.3 nmol/kg) | 0.08393 | -0.5108 to 0.6786 | No | ns | 0.9835 |
| Vehicle vs. Cagrilintide (3 nmol/kg) | -0.02857 | -0.6233 to 0.5661 | No | ns | 0.9993 |
| Vehicle vs. Cagrilintide (30 nmol/kg) | -0.3571 | -0.9713 to 0.2571 | No | ns | 0.4392 |
| Cagrilintide (0.3 nmol/kg) vs. Cagrilintide (3 nmol/kg) | -0.1125 | -0.6870 to 0.4620 | No | ns | 0.9580 |
| Cagrilintide (0.3 nmol/kg) vs. Cagrilintide (30 nmol/kg) | -0.4411 | -1.036 to 0.1536 | No | ns | 0.2245 |
| Cagrilintide (3 nmol/kg) vs. Cagrilintide (30 nmol/kg) | -0.3286 | -0.9233 to 0.2661 | No | ns | 0.4851 |
|  |  |  |  |  |  |
| 20 |  |  |  |  |  |
| Vehicle vs. Cagrilintide (0.3 nmol/kg) | -0.02679 | -0.6215 to 0.5679 | No | ns | 0.9994 |
| Vehicle vs. Cagrilintide (3 nmol/kg) | -0.2018 | -0.7965 to 0.3929 | No | ns | 0.8182 |
| Vehicle vs. Cagrilintide (30 nmol/kg) | -0.2286 | -0.8428 to 0.3856 | No | ns | 0.7728 |
| Cagrilintide (0.3 nmol/kg) vs. Cagrilintide (3 nmol/kg) | -0.1750 | -0.7495 to 0.3995 | No | ns | 0.8614 |
| Cagrilintide (0.3 nmol/kg) vs. Cagrilintide (30 nmol/kg) | -0.2018 | -0.7965 to 0.3929 | No | ns | 0.8182 |
| Cagrilintide (3 nmol/kg) vs. Cagrilintide (30 nmol/kg) | -0.02679 | -0.6215 to 0.5679 | No | ns | 0.9994 |
|  |  |  |  |  |  |
| 21 |  |  |  |  |  |
| Vehicle vs. Cagrilintide (0.3 nmol/kg) | -0.1143 | -0.7090 to 0.4804 | No | ns | 0.9602 |
| Vehicle vs. Cagrilintide (3 nmol/kg) | -0.1268 | -0.7215 to 0.4679 | No | ns | 0.9467 |
| Vehicle vs. Cagrilintide (30 nmol/kg) | 0.02857 | -0.5856 to 0.6428 | No | ns | 0.9994 |
| Cagrilintide (0.3 nmol/kg) vs. Cagrilintide (3 nmol/kg) | -0.01250 | -0.5870 to 0.5620 | No | ns | >0.9999 |
| Cagrilintide (0.3 nmol/kg) vs. Cagrilintide (30 nmol/kg) | 0.1429 | -0.4518 to 0.7375 | No | ns | 0.9260 |
| Cagrilintide (3 nmol/kg) vs. Cagrilintide (30 nmol/kg) | 0.1554 | -0.4393 to 0.7500 | No | ns | 0.9072 |
|  |  |  |  |  |  |
| 22 |  |  |  |  |  |
| Vehicle vs. Cagrilintide (0.3 nmol/kg) | -0.07500 | -0.6697 to 0.5197 | No | ns | 0.9881 |
| Vehicle vs. Cagrilintide (3 nmol/kg) | -0.1875 | -0.7822 to 0.4072 | No | ns | 0.8486 |
| Vehicle vs. Cagrilintide (30 nmol/kg) | 0.08571 | -0.5285 to 0.6999 | No | ns | 0.9841 |
| Cagrilintide (0.3 nmol/kg) vs. Cagrilintide (3 nmol/kg) | -0.1125 | -0.6870 to 0.4620 | No | ns | 0.9580 |
| Cagrilintide (0.3 nmol/kg) vs. Cagrilintide (30 nmol/kg) | 0.1607 | -0.4340 to 0.7554 | No | ns | 0.8985 |
| Cagrilintide (3 nmol/kg) vs. Cagrilintide (30 nmol/kg) | 0.2732 | -0.3215 to 0.8679 | No | ns | 0.6374 |

**Figure 1b: weekly food intake**

Statistical test: Two-way Anova. Factors: group and week.

Weekly food intake is independent from the previous week so no repeated measure was performed.

| Two-way ANOVA | Ordinary |  |  |  |  |
| --- | --- | --- | --- | --- | --- |
| Alpha | 0.05 |  |  |  |  |
|  |  |  |  |  |  |
| Source of Variation | % of total variation | P value | P value summary | Significant? |  |
| Interaction | 21.31 | 0.0026 | ** | Yes |  |
| Week | 0.3109 | 0.8495 | ns | No |  |
| Group | 4.141 | 0.2344 | ns | No |  |
|  |  |  |  |  |  |
| ANOVA table | SS (Type III) | DF | MS | F (DFn, DFd) | P value |
| Interaction | 127.8 | 6 | 21.31 | F (6, 78) = 3.734 | P=0.0026 |
| Week | 1.865 | 2 | 0.9323 | F (2, 78) = 0.1634 | P=0.8495 |
| Group | 24.84 | 3 | 8.279 | F (3, 78) = 1.451 | P=0.2344 |
| Residual | 445.0 | 78 | 5.705 |  |  |

Post Hoc multiple comparison

| Tukey's multiple comparisons test | Predicted (LS) mean diff. | 95.00% CI of diff. | Below threshold? | Summary | Adjusted P Value |
| --- | --- | --- | --- | --- | --- |
|  |  |  |  |  |  |
| Week 1 |  |  |  |  |  |
| Vehicle vs. Cagrilintide (0.3 nmol/kg) | 1.173 | -2.072 to 4.419 | No | ns | 0.7785 |
| Vehicle vs. Cagrilintide (3 nmol/kg) | 5.098 | 1.853 to 8.344 | Yes | *** | 0.0005 |
| Vehicle vs. Cagrilintide (30 nmol/kg) | 4.314 | 0.9624 to 7.666 | Yes | ** | 0.0061 |
| Cagrilintide (0.3 nmol/kg) vs. Cagrilintide (3 nmol/kg) | 3.925 | 0.7896 to 7.060 | Yes | ** | 0.0081 |
| Cagrilintide (0.3 nmol/kg) vs. Cagrilintide (30 nmol/kg) | 3.141 | -0.1043 to 6.386 | No | ns | 0.0614 |
| Cagrilintide (3 nmol/kg) vs. Cagrilintide (30 nmol/kg) | -0.7839 | -4.029 to 2.461 | No | ns | 0.9207 |
|  |  |  |  |  |  |
| Week 2 |  |  |  |  |  |
| Vehicle vs. Cagrilintide (0.3 nmol/kg) | -1.007 | -4.253 to 2.238 | No | ns | 0.8474 |
| Vehicle vs. Cagrilintide (3 nmol/kg) | -0.8321 | -4.078 to 2.413 | No | ns | 0.9069 |
| Vehicle vs. Cagrilintide (30 nmol/kg) | -1.971 | -5.323 to 1.380 | No | ns | 0.4165 |
| Cagrilintide (0.3 nmol/kg) vs. Cagrilintide (3 nmol/kg) | 0.1750 | -2.960 to 3.310 | No | ns | 0.9989 |
| Cagrilintide (0.3 nmol/kg) vs. Cagrilintide (30 nmol/kg) | -0.9643 | -4.210 to 2.281 | No | ns | 0.8633 |
| Cagrilintide (3 nmol/kg) vs. Cagrilintide (30 nmol/kg) | -1.139 | -4.385 to 2.106 | No | ns | 0.7934 |
|  |  |  |  |  |  |
| Week 3 |  |  |  |  |  |
| Vehicle vs. Cagrilintide (0.3 nmol/kg) | 0.2143 | -3.031 to 3.460 | No | ns | 0.9981 |
| Vehicle vs. Cagrilintide (3 nmol/kg) | -0.2982 | -3.544 to 2.947 | No | ns | 0.9950 |
| Vehicle vs. Cagrilintide (30 nmol/kg) | -0.8857 | -4.238 to 2.466 | No | ns | 0.8992 |
| Cagrilintide (0.3 nmol/kg) vs. Cagrilintide (3 nmol/kg) | -0.5125 | -3.648 to 2.623 | No | ns | 0.9733 |
| Cagrilintide (0.3 nmol/kg) vs. Cagrilintide (30 nmol/kg) | -1.100 | -4.345 to 2.145 | No | ns | 0.8101 |
| Cagrilintide (3 nmol/kg) vs. Cagrilintide (30 nmol/kg) | -0.5875 | -3.833 to 2.658 | No | ns | 0.9643 |

**Figure 1c: daily body weight**

Statistical test: Two-way Anova with repeated measure. Factors: group and time.

As body weight values are not independent from each other, repeated analysis was performed.

| Two-way RM ANOVA | Matching: Stacked |  |  |  |  |
| --- | --- | --- | --- | --- | --- |
| Assume sphericity? | No |  |  |  |  |
| Alpha | 0.05 |  |  |  |  |
|  |  |  |  |  |  |
| Source of Variation | % of total variation | P value | P value summary | Significant? | Geisser-Greenhouse's epsilon |
| Time x Group | 0.3405 | <0.0001 | **** | Yes |  |
| Time | 0.3726 | <0.0001 | **** | Yes | 0.1264 |
| Group | 2.801 | 0.8579 | ns | No |  |
| Subject | 95.66 | <0.0001 | **** | Yes |  |
|  |  |  |  |  |  |
| ANOVA table | SS | DF | MS | F (DFn, DFd) | P value |
| Time x Group | 74.27 | 66 | 1.125 | F (66, 572) = 3.575 | P<0.0001 |
| Time | 81.27 | 22 | 3.694 | F (2.782, 72.32) = 11.74 | P<0.0001 |
| Group | 610.9 | 3 | 203.6 | F (3, 26) = 0.2538 | P=0.8579 |
| Subject | 20864 | 26 | 802.5 | F (26, 572) = 2549 | P<0.0001 |
| Residual | 180.0 | 572 | 0.3148 |  |  |

Post Hoc multiple comparison

No significant differences were observed

**Figure 1d: cumulative daily body weight gain**

Statistical test: Two-way Anova with repeated measure. Factors: group and time.

As body weight values are not independent from each other, repeated analysis was performed.

| Two-way RM ANOVA | Matching: Stacked |  |  |  |  |
| --- | --- | --- | --- | --- | --- |
| Assume sphericity? | No |  |  |  |  |
| Alpha | 0.05 |  |  |  |  |
|  |  |  |  |  |  |
| Source of Variation | % of total variation | P value | P value summary | Significant? | Geisser-Greenhouse's epsilon |
| Time x Group | 4.551 | <0.0001 | **** | Yes |  |
| Time | 4.980 | <0.0001 | **** | Yes | 0.1264 |
| Group | 58.02 | <0.0001 | **** | Yes |  |
| Subject | 21.35 | <0.0001 | **** | Yes |  |
|  |  |  |  |  |  |
| ANOVA table | SS | DF | MS | F (DFn, DFd) | P value |
| Time x Group | 74.27 | 66 | 1.125 | F (66, 572) = 3.575 | P<0.0001 |
| Time | 81.27 | 22 | 3.694 | F (2.782, 72.32) = 11.74 | P<0.0001 |
| Group | 946.8 | 3 | 315.6 | F (3, 26) = 23.56 | P<0.0001 |
| Subject | 348.3 | 26 | 13.40 | F (26, 572) = 42.56 | P<0.0001 |
| Residual | 180.0 | 572 | 0.3148 |  |  |

Post Hoc multiple comparison

| Tukey's multiple comparisons test | Mean Diff. | 95.00% CI of diff. | Below threshold? | Summary | Adjusted P Value |
| --- | --- | --- | --- | --- | --- |
|  |  |  |  |  |  |
| 0 |  |  |  |  |  |
| Vehicle vs. Cagrilintide (0.3 nmol/kg) | 0.000 |  |  |  |  |
| Vehicle vs. Cagrilintide (3 nmol/kg) | 0.000 |  |  |  |  |
| Vehicle vs. Cagrilintide (30 nmol/kg) | 0.000 |  |  |  |  |
| Cagrilintide (0.3 nmol/kg) vs. Cagrilintide (3 nmol/kg) | 0.000 |  |  |  |  |
| Cagrilintide (0.3 nmol/kg) vs. Cagrilintide (30 nmol/kg) | 0.000 |  |  |  |  |
| Cagrilintide (3 nmol/kg) vs. Cagrilintide (30 nmol/kg) | 0.000 |  |  |  |  |
|  |  |  |  |  |  |
| 1 |  |  |  |  |  |
| Vehicle vs. Cagrilintide (0.3 nmol/kg) | 0.5679 | 0.1192 to 1.017 | Yes | * | 0.0129 |
| Vehicle vs. Cagrilintide (3 nmol/kg) | 1.405 | 0.6029 to 2.208 | Yes | ** | 0.0020 |
| Vehicle vs. Cagrilintide (30 nmol/kg) | 1.643 | 1.244 to 2.042 | Yes | **** | <0.0001 |
| Cagrilintide (0.3 nmol/kg) vs. Cagrilintide (3 nmol/kg) | 0.8375 | 0.01136 to 1.664 | Yes | * | 0.0467 |
| Cagrilintide (0.3 nmol/kg) vs. Cagrilintide (30 nmol/kg) | 1.075 | 0.5852 to 1.565 | Yes | *** | 0.0001 |
| Cagrilintide (3 nmol/kg) vs. Cagrilintide (30 nmol/kg) | 0.2375 | -0.5760 to 1.051 | No | ns | 0.8052 |
|  |  |  |  |  |  |
| 2 |  |  |  |  |  |
| Vehicle vs. Cagrilintide (0.3 nmol/kg) | 0.7964 | 0.02613 to 1.567 | Yes | * | 0.0419 |
| Vehicle vs. Cagrilintide (3 nmol/kg) | 2.246 | 1.357 to 3.136 | Yes | **** | <0.0001 |
| Vehicle vs. Cagrilintide (30 nmol/kg) | 2.557 | 1.797 to 3.318 | Yes | **** | <0.0001 |
| Cagrilintide (0.3 nmol/kg) vs. Cagrilintide (3 nmol/kg) | 1.450 | 0.5184 to 2.382 | Yes | ** | 0.0025 |
| Cagrilintide (0.3 nmol/kg) vs. Cagrilintide (30 nmol/kg) | 1.761 | 0.9454 to 2.576 | Yes | *** | 0.0001 |
| Cagrilintide (3 nmol/kg) vs. Cagrilintide (30 nmol/kg) | 0.3107 | -0.6129 to 1.234 | No | ns | 0.7568 |
|  |  |  |  |  |  |
| 3 |  |  |  |  |  |
| Vehicle vs. Cagrilintide (0.3 nmol/kg) | 0.7089 | -0.01616 to 1.434 | No | ns | 0.0562 |
| Vehicle vs. Cagrilintide (3 nmol/kg) | 2.396 | 1.749 to 3.043 | Yes | **** | <0.0001 |
| Vehicle vs. Cagrilintide (30 nmol/kg) | 2.871 | 1.921 to 3.822 | Yes | **** | <0.0001 |
| Cagrilintide (0.3 nmol/kg) vs. Cagrilintide (3 nmol/kg) | 1.688 | 0.9827 to 2.392 | Yes | **** | <0.0001 |
| Cagrilintide (0.3 nmol/kg) vs. Cagrilintide (30 nmol/kg) | 2.163 | 1.185 to 3.140 | Yes | *** | 0.0002 |
| Cagrilintide (3 nmol/kg) vs. Cagrilintide (30 nmol/kg) | 0.4750 | -0.4655 to 1.416 | No | ns | 0.4447 |
|  |  |  |  |  |  |
| 4 |  |  |  |  |  |
| Vehicle vs. Cagrilintide (0.3 nmol/kg) | 0.7250 | -0.3314 to 1.781 | No | ns | 0.2244 |
| Vehicle vs. Cagrilintide (3 nmol/kg) | 2.725 | 1.701 to 3.749 | Yes | **** | <0.0001 |
| Vehicle vs. Cagrilintide (30 nmol/kg) | 3.071 | 1.953 to 4.190 | Yes | **** | <0.0001 |
| Cagrilintide (0.3 nmol/kg) vs. Cagrilintide (3 nmol/kg) | 2.000 | 1.214 to 2.786 | Yes | **** | <0.0001 |
| Cagrilintide (0.3 nmol/kg) vs. Cagrilintide (30 nmol/kg) | 2.346 | 1.406 to 3.287 | Yes | **** | <0.0001 |
| Cagrilintide (3 nmol/kg) vs. Cagrilintide (30 nmol/kg) | 0.3464 | -0.5508 to 1.244 | No | ns | 0.6635 |
|  |  |  |  |  |  |
| 5 |  |  |  |  |  |
| Vehicle vs. Cagrilintide (0.3 nmol/kg) | 0.5821 | -0.2824 to 1.447 | No | ns | 0.2459 |
| Vehicle vs. Cagrilintide (3 nmol/kg) | 2.807 | 1.973 to 3.641 | Yes | **** | <0.0001 |
| Vehicle vs. Cagrilintide (30 nmol/kg) | 3.129 | 1.948 to 4.309 | Yes | **** | <0.0001 |
| Cagrilintide (0.3 nmol/kg) vs. Cagrilintide (3 nmol/kg) | 2.225 | 1.355 to 3.095 | Yes | **** | <0.0001 |
| Cagrilintide (0.3 nmol/kg) vs. Cagrilintide (30 nmol/kg) | 2.546 | 1.349 to 3.744 | Yes | *** | 0.0003 |
| Cagrilintide (3 nmol/kg) vs. Cagrilintide (30 nmol/kg) | 0.3214 | -0.8620 to 1.505 | No | ns | 0.8401 |
|  |  |  |  |  |  |
| 6 |  |  |  |  |  |
| Vehicle vs. Cagrilintide (0.3 nmol/kg) | 0.6411 | -0.4651 to 1.747 | No | ns | 0.3486 |
| Vehicle vs. Cagrilintide (3 nmol/kg) | 2.979 | 1.925 to 4.032 | Yes | **** | <0.0001 |
| Vehicle vs. Cagrilintide (30 nmol/kg) | 3.057 | 1.667 to 4.447 | Yes | *** | 0.0002 |
| Cagrilintide (0.3 nmol/kg) vs. Cagrilintide (3 nmol/kg) | 2.338 | 1.577 to 3.098 | Yes | **** | <0.0001 |
| Cagrilintide (0.3 nmol/kg) vs. Cagrilintide (30 nmol/kg) | 2.416 | 1.149 to 3.683 | Yes | *** | 0.0008 |
| Cagrilintide (3 nmol/kg) vs. Cagrilintide (30 nmol/kg) | 0.07857 | -1.154 to 1.311 | No | ns | 0.9968 |
|  |  |  |  |  |  |
| 7 |  |  |  |  |  |
| Vehicle vs. Cagrilintide (0.3 nmol/kg) | 0.6625 | -0.3753 to 1.700 | No | ns | 0.2854 |
| Vehicle vs. Cagrilintide (3 nmol/kg) | 3.113 | 2.237 to 3.988 | Yes | **** | <0.0001 |
| Vehicle vs. Cagrilintide (30 nmol/kg) | 3.071 | 1.710 to 4.433 | Yes | *** | 0.0002 |
| Cagrilintide (0.3 nmol/kg) vs. Cagrilintide (3 nmol/kg) | 2.450 | 1.517 to 3.383 | Yes | **** | <0.0001 |
| Cagrilintide (0.3 nmol/kg) vs. Cagrilintide (30 nmol/kg) | 2.409 | 1.024 to 3.794 | Yes | ** | 0.0014 |
| Cagrilintide (3 nmol/kg) vs. Cagrilintide (30 nmol/kg) | -0.04107 | -1.355 to 1.273 | No | ns | 0.9996 |
|  |  |  |  |  |  |
| 8 |  |  |  |  |  |
| Vehicle vs. Cagrilintide (0.3 nmol/kg) | 0.8357 | -0.4189 to 2.090 | No | ns | 0.2482 |
| Vehicle vs. Cagrilintide (3 nmol/kg) | 3.311 | 2.096 to 4.525 | Yes | **** | <0.0001 |
| Vehicle vs. Cagrilintide (30 nmol/kg) | 3.271 | 1.792 to 4.751 | Yes | *** | 0.0001 |
| Cagrilintide (0.3 nmol/kg) vs. Cagrilintide (3 nmol/kg) | 2.475 | 1.466 to 3.484 | Yes | **** | <0.0001 |
| Cagrilintide (0.3 nmol/kg) vs. Cagrilintide (30 nmol/kg) | 2.436 | 1.075 to 3.797 | Yes | ** | 0.0011 |
| Cagrilintide (3 nmol/kg) vs. Cagrilintide (30 nmol/kg) | -0.03929 | -1.368 to 1.289 | No | ns | 0.9997 |
|  |  |  |  |  |  |
| 9 |  |  |  |  |  |
| Vehicle vs. Cagrilintide (0.3 nmol/kg) | 0.3679 | -1.250 to 1.986 | No | ns | 0.9001 |
| Vehicle vs. Cagrilintide (3 nmol/kg) | 2.993 | 1.452 to 4.534 | Yes | ** | 0.0011 |
| Vehicle vs. Cagrilintide (30 nmol/kg) | 2.714 | 0.9833 to 4.445 | Yes | ** | 0.0028 |
| Cagrilintide (0.3 nmol/kg) vs. Cagrilintide (3 nmol/kg) | 2.625 | 1.571 to 3.679 | Yes | **** | <0.0001 |
| Cagrilintide (0.3 nmol/kg) vs. Cagrilintide (30 nmol/kg) | 2.346 | 0.9251 to 3.768 | Yes | ** | 0.0018 |
| Cagrilintide (3 nmol/kg) vs. Cagrilintide (30 nmol/kg) | -0.2786 | -1.586 to 1.029 | No | ns | 0.9076 |
|  |  |  |  |  |  |
| 10 |  |  |  |  |  |
| Vehicle vs. Cagrilintide (0.3 nmol/kg) | 0.09821 | -1.814 to 2.010 | No | ns | 0.9986 |
| Vehicle vs. Cagrilintide (3 nmol/kg) | 2.573 | 0.7526 to 4.394 | Yes | ** | 0.0090 |
| Vehicle vs. Cagrilintide (30 nmol/kg) | 2.243 | 0.2056 to 4.280 | Yes | * | 0.0299 |
| Cagrilintide (0.3 nmol/kg) vs. Cagrilintide (3 nmol/kg) | 2.475 | 1.293 to 3.657 | Yes | *** | 0.0003 |
| Cagrilintide (0.3 nmol/kg) vs. Cagrilintide (30 nmol/kg) | 2.145 | 0.5003 to 3.789 | Yes | * | 0.0103 |
| Cagrilintide (3 nmol/kg) vs. Cagrilintide (30 nmol/kg) | -0.3304 | -1.826 to 1.165 | No | ns | 0.8917 |
|  |  |  |  |  |  |
| 11 |  |  |  |  |  |
| Vehicle vs. Cagrilintide (0.3 nmol/kg) | 0.3089 | -1.679 to 2.297 | No | ns | 0.9602 |
| Vehicle vs. Cagrilintide (3 nmol/kg) | 2.809 | 0.8503 to 4.768 | Yes | ** | 0.0087 |
| Vehicle vs. Cagrilintide (30 nmol/kg) | 2.443 | 0.3019 to 4.584 | Yes | * | 0.0245 |
| Cagrilintide (0.3 nmol/kg) vs. Cagrilintide (3 nmol/kg) | 2.500 | 1.518 to 3.482 | Yes | **** | <0.0001 |
| Cagrilintide (0.3 nmol/kg) vs. Cagrilintide (30 nmol/kg) | 2.134 | 0.5727 to 3.695 | Yes | ** | 0.0082 |
| Cagrilintide (3 nmol/kg) vs. Cagrilintide (30 nmol/kg) | -0.3661 | -1.854 to 1.122 | No | ns | 0.8551 |
|  |  |  |  |  |  |
| 12 |  |  |  |  |  |
| Vehicle vs. Cagrilintide (0.3 nmol/kg) | 0.03036 | -1.665 to 1.726 | No | ns | >0.9999 |
| Vehicle vs. Cagrilintide (3 nmol/kg) | 2.730 | 1.135 to 4.326 | Yes | ** | 0.0024 |
| Vehicle vs. Cagrilintide (30 nmol/kg) | 2.414 | 0.5265 to 4.302 | Yes | * | 0.0118 |
| Cagrilintide (0.3 nmol/kg) vs. Cagrilintide (3 nmol/kg) | 2.700 | 1.534 to 3.866 | Yes | **** | <0.0001 |
| Cagrilintide (0.3 nmol/kg) vs. Cagrilintide (30 nmol/kg) | 2.384 | 0.7396 to 4.028 | Yes | ** | 0.0051 |
| Cagrilintide (3 nmol/kg) vs. Cagrilintide (30 nmol/kg) | -0.3161 | -1.851 to 1.219 | No | ns | 0.9136 |
|  |  |  |  |  |  |
| 13 |  |  |  |  |  |
| Vehicle vs. Cagrilintide (0.3 nmol/kg) | 0.2304 | -1.526 to 1.986 | No | ns | 0.9783 |
| Vehicle vs. Cagrilintide (3 nmol/kg) | 2.755 | 1.116 to 4.395 | Yes | ** | 0.0033 |
| Vehicle vs. Cagrilintide (30 nmol/kg) | 2.486 | 0.5436 to 4.428 | Yes | * | 0.0117 |
| Cagrilintide (0.3 nmol/kg) vs. Cagrilintide (3 nmol/kg) | 2.525 | 1.369 to 3.681 | Yes | *** | 0.0003 |
| Cagrilintide (0.3 nmol/kg) vs. Cagrilintide (30 nmol/kg) | 2.255 | 0.5731 to 3.938 | Yes | ** | 0.0087 |
| Cagrilintide (3 nmol/kg) vs. Cagrilintide (30 nmol/kg) | -0.2696 | -1.818 to 1.279 | No | ns | 0.9394 |
|  |  |  |  |  |  |
| 14 |  |  |  |  |  |
| Vehicle vs. Cagrilintide (0.3 nmol/kg) | 0.2089 | -1.396 to 1.814 | No | ns | 0.9794 |
| Vehicle vs. Cagrilintide (3 nmol/kg) | 2.671 | 1.201 to 4.142 | Yes | ** | 0.0016 |
| Vehicle vs. Cagrilintide (30 nmol/kg) | 2.400 | 0.1336 to 4.666 | Yes | * | 0.0373 |
| Cagrilintide (0.3 nmol/kg) vs. Cagrilintide (3 nmol/kg) | 2.463 | 1.296 to 3.629 | Yes | *** | 0.0003 |
| Cagrilintide (0.3 nmol/kg) vs. Cagrilintide (30 nmol/kg) | 2.191 | 0.01266 to 4.369 | Yes | * | 0.0486 |
| Cagrilintide (3 nmol/kg) vs. Cagrilintide (30 nmol/kg) | -0.2714 | -2.401 to 1.858 | No | ns | 0.9735 |
|  |  |  |  |  |  |
| 15 |  |  |  |  |  |
| Vehicle vs. Cagrilintide (0.3 nmol/kg) | 0.07143 | -1.536 to 1.679 | No | ns | 0.9991 |
| Vehicle vs. Cagrilintide (3 nmol/kg) | 2.509 | 1.045 to 3.973 | Yes | ** | 0.0021 |
| Vehicle vs. Cagrilintide (30 nmol/kg) | 2.314 | 0.3153 to 4.313 | Yes | * | 0.0224 |
| Cagrilintide (0.3 nmol/kg) vs. Cagrilintide (3 nmol/kg) | 2.438 | 1.226 to 3.649 | Yes | *** | 0.0004 |
| Cagrilintide (0.3 nmol/kg) vs. Cagrilintide (30 nmol/kg) | 2.243 | 0.3490 to 4.137 | Yes | * | 0.0200 |
| Cagrilintide (3 nmol/kg) vs. Cagrilintide (30 nmol/kg) | -0.1946 | -2.001 to 1.612 | No | ns | 0.9848 |
|  |  |  |  |  |  |
| 16 |  |  |  |  |  |
| Vehicle vs. Cagrilintide (0.3 nmol/kg) | 0.3696 | -1.432 to 2.171 | No | ns | 0.9273 |
| Vehicle vs. Cagrilintide (3 nmol/kg) | 2.982 | 1.232 to 4.732 | Yes | ** | 0.0015 |
| Vehicle vs. Cagrilintide (30 nmol/kg) | 2.629 | 0.4941 to 4.763 | Yes | * | 0.0152 |
| Cagrilintide (0.3 nmol/kg) vs. Cagrilintide (3 nmol/kg) | 2.613 | 1.113 to 4.112 | Yes | *** | 0.0009 |
| Cagrilintide (0.3 nmol/kg) vs. Cagrilintide (30 nmol/kg) | 2.259 | 0.2709 to 4.247 | Yes | * | 0.0251 |
| Cagrilintide (3 nmol/kg) vs. Cagrilintide (30 nmol/kg) | -0.3536 | -2.301 to 1.594 | No | ns | 0.9441 |
|  |  |  |  |  |  |
| 17 |  |  |  |  |  |
| Vehicle vs. Cagrilintide (0.3 nmol/kg) | 0.3786 | -1.441 to 2.198 | No | ns | 0.9249 |
| Vehicle vs. Cagrilintide (3 nmol/kg) | 2.929 | 1.213 to 4.644 | Yes | ** | 0.0016 |
| Vehicle vs. Cagrilintide (30 nmol/kg) | 2.529 | 0.2734 to 4.784 | Yes | * | 0.0270 |
| Cagrilintide (0.3 nmol/kg) vs. Cagrilintide (3 nmol/kg) | 2.550 | 1.076 to 4.024 | Yes | ** | 0.0010 |
| Cagrilintide (0.3 nmol/kg) vs. Cagrilintide (30 nmol/kg) | 2.150 | 0.01114 to 4.289 | Yes | * | 0.0487 |
| Cagrilintide (3 nmol/kg) vs. Cagrilintide (30 nmol/kg) | -0.4000 | -2.471 to 1.671 | No | ns | 0.9298 |
|  |  |  |  |  |  |
| 18 |  |  |  |  |  |
| Vehicle vs. Cagrilintide (0.3 nmol/kg) | 0.4482 | -1.350 to 2.247 | No | ns | 0.8781 |
| Vehicle vs. Cagrilintide (3 nmol/kg) | 2.973 | 1.254 to 4.692 | Yes | ** | 0.0016 |
| Vehicle vs. Cagrilintide (30 nmol/kg) | 2.257 | 0.09342 to 4.421 | Yes | * | 0.0401 |
| Cagrilintide (0.3 nmol/kg) vs. Cagrilintide (3 nmol/kg) | 2.525 | 1.128 to 3.922 | Yes | *** | 0.0007 |
| Cagrilintide (0.3 nmol/kg) vs. Cagrilintide (30 nmol/kg) | 1.809 | -0.1871 to 3.805 | No | ns | 0.0794 |
| Cagrilintide (3 nmol/kg) vs. Cagrilintide (30 nmol/kg) | -0.7161 | -2.652 to 1.219 | No | ns | 0.6735 |
|  |  |  |  |  |  |
| 19 |  |  |  |  |  |
| Vehicle vs. Cagrilintide (0.3 nmol/kg) | 0.6161 | -1.256 to 2.488 | No | ns | 0.7620 |
| Vehicle vs. Cagrilintide (3 nmol/kg) | 3.066 | 1.268 to 4.864 | Yes | ** | 0.0018 |
| Vehicle vs. Cagrilintide (30 nmol/kg) | 2.257 | -0.003676 to 4.518 | No | ns | 0.0504 |
| Cagrilintide (0.3 nmol/kg) vs. Cagrilintide (3 nmol/kg) | 2.450 | 1.015 to 3.885 | Yes | ** | 0.0011 |
| Cagrilintide (0.3 nmol/kg) vs. Cagrilintide (30 nmol/kg) | 1.641 | -0.4321 to 3.714 | No | ns | 0.1374 |
| Cagrilintide (3 nmol/kg) vs. Cagrilintide (30 nmol/kg) | -0.8089 | -2.826 to 1.208 | No | ns | 0.6180 |
|  |  |  |  |  |  |
| 20 |  |  |  |  |  |
| Vehicle vs. Cagrilintide (0.3 nmol/kg) | 0.8464 | -1.078 to 2.771 | No | ns | 0.5814 |
| Vehicle vs. Cagrilintide (3 nmol/kg) | 3.109 | 1.402 to 4.816 | Yes | ** | 0.0013 |
| Vehicle vs. Cagrilintide (30 nmol/kg) | 2.657 | 0.5751 to 4.739 | Yes | * | 0.0119 |
| Cagrilintide (0.3 nmol/kg) vs. Cagrilintide (3 nmol/kg) | 2.263 | 0.7361 to 3.789 | Yes | ** | 0.0043 |
| Cagrilintide (0.3 nmol/kg) vs. Cagrilintide (30 nmol/kg) | 1.811 | -0.1685 to 3.790 | No | ns | 0.0773 |
| Cagrilintide (3 nmol/kg) vs. Cagrilintide (30 nmol/kg) | -0.4518 | -2.230 to 1.326 | No | ns | 0.8566 |
|  |  |  |  |  |  |
| 21 |  |  |  |  |  |
| Vehicle vs. Cagrilintide (0.3 nmol/kg) | 0.5393 | -1.285 to 2.364 | No | ns | 0.8210 |
| Vehicle vs. Cagrilintide (3 nmol/kg) | 2.877 | 1.330 to 4.423 | Yes | ** | 0.0011 |
| Vehicle vs. Cagrilintide (30 nmol/kg) | 2.443 | 0.2655 to 4.620 | Yes | * | 0.0270 |
| Cagrilintide (0.3 nmol/kg) vs. Cagrilintide (3 nmol/kg) | 2.338 | 0.8239 to 3.851 | Yes | ** | 0.0034 |
| Cagrilintide (0.3 nmol/kg) vs. Cagrilintide (30 nmol/kg) | 1.904 | -0.2641 to 4.071 | No | ns | 0.0921 |
| Cagrilintide (3 nmol/kg) vs. Cagrilintide (30 nmol/kg) | -0.4339 | -2.436 to 1.568 | No | ns | 0.8969 |
|  |  |  |  |  |  |
| 22 |  |  |  |  |  |
| Vehicle vs. Cagrilintide (0.3 nmol/kg) | 0.6446 | -1.199 to 2.489 | No | ns | 0.7369 |
| Vehicle vs. Cagrilintide (3 nmol/kg) | 2.795 | 1.392 to 4.198 | Yes | *** | 0.0009 |
| Vehicle vs. Cagrilintide (30 nmol/kg) | 2.500 | 0.3636 to 4.636 | Yes | * | 0.0214 |
| Cagrilintide (0.3 nmol/kg) vs. Cagrilintide (3 nmol/kg) | 2.150 | 0.5441 to 3.756 | Yes | * | 0.0105 |
| Cagrilintide (0.3 nmol/kg) vs. Cagrilintide (30 nmol/kg) | 1.855 | -0.3769 to 4.088 | No | ns | 0.1167 |
| Cagrilintide (3 nmol/kg) vs. Cagrilintide (30 nmol/kg) | -0.2946 | -2.294 to 1.705 | No | ns | 0.9603 |

**Figure 2a: daily food intake**

Statistical test: Three-way Anova. Factors: genotype, treatment and days

*# running the classical 3-way ANOVA with interaction between all terms. (Do not use aov() here as it would ignore the (1|Mouse_ID random effect))*

anova(out[[2]]) *# choosing second model (full interaction model) from the lmer_time_result function.*

## Type III Analysis of Variance Table with Satterthwaite's method

## Sum Sq Mean Sq NumDF DenDF F value Pr(>F)

## Genotype 0.2800 0.28004 1 38 1.9984 0.165608

## Treatement 1.0738 0.53691 2 38 3.8315 0.030492 *

## time 28.7277 1.36799 21 798 9.7621 < 2.2e-16 ***

## Genotype:Treatement 0.1312 0.06559 2 38 0.4681 0.629781

## Genotype:time 6.6358 0.31599 21 798 2.2550 0.001081 **

## Treatement:time 28.4028 0.67626 42 798 4.8259 < 2.2e-16 ***

## Genotype:Treatement:time 18.5263 0.44110 42 798 3.1478 3.159e-10 ***

## ---

## Signif. codes: 0 '***' 0.001 '**' 0.01 '*' 0.05 '.' 0.1 ' ' 1

*# runnning pairwise comparisons on interaction model, full 15 comparisons per timepoint*

*# model: m1 <- lmer(bw ~ Genotype * Treatement * Week + (1|Mouse_ID), data = weekly_fi_long)*

emm_results <- emmeans(out[[2]], pairwise ~ Genotype * Treatement | time, adjust = "tukey")

summary(emm_results)

## $contrasts

## time = D0:

## contrast estimate SE df t.ratio p.value

## WT Veh - RAMP Veh -0.32857 0.305 120 -1.079 0.8889

## WT Veh - WT Cagri -0.11429 0.293 120 -0.391 0.9988

## WT Veh - RAMP Cagri -0.05357 0.283 120 -0.189 1.0000

## WT Veh - WT sCT 0.12143 0.283 120 0.429 0.9981

## WT Veh - RAMP sCT -0.31607 0.283 120 -1.116 0.8741

## RAMP Veh - WT Cagri 0.21429 0.305 120 0.704 0.9812

## RAMP Veh - RAMP Cagri 0.27500 0.296 120 0.930 0.9380

## RAMP Veh - WT sCT 0.45000 0.296 120 1.522 0.6508

## RAMP Veh - RAMP sCT 0.01250 0.296 120 0.042 1.0000

## WT Cagri - RAMP Cagri 0.06071 0.283 120 0.214 0.9999

## WT Cagri - WT sCT 0.23571 0.283 120 0.832 0.9610

## WT Cagri - RAMP sCT -0.20179 0.283 120 -0.712 0.9801

## RAMP Cagri - WT sCT 0.17500 0.274 120 0.639 0.9878

## RAMP Cagri - RAMP sCT -0.26250 0.274 120 -0.959 0.9298

## WT sCT - RAMP sCT -0.43750 0.274 120 -1.598 0.6012

##

## time = D1:

## contrast estimate SE df t.ratio p.value

## WT Veh - RAMP Veh -0.29524 0.305 120 -0.969 0.9267

**## WT Veh - WT Cagri 1.41429 0.293 120 4.833 0.0001**

## WT Veh - RAMP Cagri 0.27143 0.283 120 0.958 0.9301

**## WT Veh - WT sCT 1.10893 0.283 120 3.914 0.0021**

## WT Veh - RAMP sCT 0.35893 0.283 120 1.267 0.8023

## RAMP Veh - WT Cagri 1.70952 0.305 120 5.613 <.0001

## RAMP Veh - RAMP Cagri 0.56667 0.296 120 1.917 0.3971

## RAMP Veh - WT sCT 1.40417 0.296 120 4.750 0.0001

## RAMP Veh - RAMP sCT 0.65417 0.296 120 2.213 0.2396

**## WT Cagri - RAMP Cagri -1.14286 0.283 120 -4.034 0.0013**

## WT Cagri - WT sCT -0.30536 0.283 120 -1.078 0.8893

## WT Cagri - RAMP sCT -1.05536 0.283 120 -3.725 0.0040

## RAMP Cagri - WT sCT 0.83750 0.274 120 3.060 0.0319

## RAMP Cagri - RAMP sCT 0.08750 0.274 120 0.320 0.9995

## WT sCT - RAMP sCT -0.75000 0.274 120 -2.740 0.0749

##

## time = D2:

## contrast estimate SE df t.ratio p.value

## WT Veh - RAMP Veh 0.06667 0.305 120 0.219 0.9999

## WT Veh - WT Cagri 0.74286 0.293 120 2.539 0.1212

## WT Veh - RAMP Cagri 0.30000 0.283 120 1.059 0.8965

## WT Veh - WT sCT 0.65000 0.283 120 2.294 0.2045

## WT Veh - RAMP sCT 0.38750 0.283 120 1.368 0.7461

## RAMP Veh - WT Cagri 0.67619 0.305 120 2.220 0.2362

## RAMP Veh - RAMP Cagri 0.23333 0.296 120 0.789 0.9689

## RAMP Veh - WT sCT 0.58333 0.296 120 1.973 0.3638

## RAMP Veh - RAMP sCT 0.32083 0.296 120 1.085 0.8864

## WT Cagri - RAMP Cagri -0.44286 0.283 120 -1.563 0.6242

## WT Cagri - WT sCT -0.09286 0.283 120 -0.328 0.9995

## WT Cagri - RAMP sCT -0.35536 0.283 120 -1.254 0.8089

## RAMP Cagri - WT sCT 0.35000 0.274 120 1.279 0.7960

## RAMP Cagri - RAMP sCT 0.08750 0.274 120 0.320 0.9995

## WT sCT - RAMP sCT -0.26250 0.274 120 -0.959 0.9298

##

## time = D3:

## contrast estimate SE df t.ratio p.value

## WT Veh - RAMP Veh -0.40000 0.305 120 -1.313 0.7771

## WT Veh - WT Cagri 0.25714 0.293 120 0.879 0.9509

## WT Veh - RAMP Cagri -0.22500 0.283 120 -0.794 0.9680

## WT Veh - WT sCT -0.07500 0.283 120 -0.265 0.9998

## WT Veh - RAMP sCT 0.26250 0.283 120 0.927 0.9389

## RAMP Veh - WT Cagri 0.65714 0.305 120 2.158 0.2655

## RAMP Veh - RAMP Cagri 0.17500 0.296 120 0.592 0.9914

## RAMP Veh - WT sCT 0.32500 0.296 120 1.099 0.8808

## RAMP Veh - RAMP sCT 0.66250 0.296 120 2.241 0.2271

## WT Cagri - RAMP Cagri -0.48214 0.283 120 -1.702 0.5333

## WT Cagri - WT sCT -0.33214 0.283 120 -1.172 0.8491

## WT Cagri - RAMP sCT 0.00536 0.283 120 0.019 1.0000

## RAMP Cagri - WT sCT 0.15000 0.274 120 0.548 0.9940

## RAMP Cagri - RAMP sCT 0.48750 0.274 120 1.781 0.4818

## WT sCT - RAMP sCT 0.33750 0.274 120 1.233 0.8198

##

## time = D4:

## contrast estimate SE df t.ratio p.value

## WT Veh - RAMP Veh -0.45000 0.305 120 -1.478 0.6791

## WT Veh - WT Cagri 0.17143 0.293 120 0.586 0.9918

## WT Veh - RAMP Cagri -0.22500 0.283 120 -0.794 0.9680

## WT Veh - WT sCT -0.70000 0.283 120 -2.471 0.1412

## WT Veh - RAMP sCT -0.28750 0.283 120 -1.015 0.9122

## RAMP Veh - WT Cagri 0.62143 0.305 120 2.040 0.3260

## RAMP Veh - RAMP Cagri 0.22500 0.296 120 0.761 0.9734

## RAMP Veh - WT sCT -0.25000 0.296 120 -0.846 0.9582

## RAMP Veh - RAMP sCT 0.16250 0.296 120 0.550 0.9939

## WT Cagri - RAMP Cagri -0.39643 0.283 120 -1.399 0.7274

## WT Cagri - WT sCT -0.87143 0.283 120 -3.076 0.0305

## WT Cagri - RAMP sCT -0.45893 0.283 120 -1.620 0.5871

## RAMP Cagri - WT sCT -0.47500 0.274 120 -1.735 0.5113

## RAMP Cagri - RAMP sCT -0.06250 0.274 120 -0.228 0.9999

## WT sCT - RAMP sCT 0.41250 0.274 120 1.507 0.6604

##

## time = D5:

## contrast estimate SE df t.ratio p.value

## WT Veh - RAMP Veh -0.30238 0.305 120 -0.993 0.9194

## WT Veh - WT Cagri 0.11429 0.293 120 0.391 0.9988

## WT Veh - RAMP Cagri -0.39821 0.283 120 -1.406 0.7236

## WT Veh - WT sCT -0.77321 0.283 120 -2.729 0.0769

## WT Veh - RAMP sCT -0.22321 0.283 120 -0.788 0.9691

## RAMP Veh - WT Cagri 0.41667 0.305 120 1.368 0.7459

## RAMP Veh - RAMP Cagri -0.09583 0.296 120 -0.324 0.9995

## RAMP Veh - WT sCT -0.47083 0.296 120 -1.593 0.6050

## RAMP Veh - RAMP sCT 0.07917 0.296 120 0.268 0.9998

## WT Cagri - RAMP Cagri -0.51250 0.283 120 -1.809 0.4640

## WT Cagri - WT sCT -0.88750 0.283 120 -3.133 0.0259

## WT Cagri - RAMP sCT -0.33750 0.283 120 -1.191 0.8403

## RAMP Cagri - WT sCT -0.37500 0.274 120 -1.370 0.7447

## RAMP Cagri - RAMP sCT 0.17500 0.274 120 0.639 0.9878

## WT sCT - RAMP sCT 0.55000 0.274 120 2.009 0.3431

##

## time = D6:

## contrast estimate SE df t.ratio p.value

## WT Veh - RAMP Veh -0.26905 0.305 120 -0.883 0.9498

## WT Veh - WT Cagri 0.00000 0.293 120 0.000 1.0000

## WT Veh - RAMP Cagri -0.28571 0.283 120 -1.008 0.9143

**## WT Veh - WT sCT -1.02321 0.283 120 -3.612 0.0058**

## WT Veh - RAMP sCT -0.29821 0.283 120 -1.053 0.8988

## RAMP Veh - WT Cagri 0.26905 0.305 120 0.883 0.9498

## RAMP Veh - RAMP Cagri -0.01667 0.296 120 -0.056 1.0000

## RAMP Veh - WT sCT -0.75417 0.296 120 -2.551 0.1179

## RAMP Veh - RAMP sCT -0.02917 0.296 120 -0.099 1.0000

## WT Cagri - RAMP Cagri -0.28571 0.283 120 -1.008 0.9143

## WT Cagri - WT sCT -1.02321 0.283 120 -3.612 0.0058

## WT Cagri - RAMP sCT -0.29821 0.283 120 -1.053 0.8988

## RAMP Cagri - WT sCT -0.73750 0.274 120 -2.695 0.0838

## RAMP Cagri - RAMP sCT -0.01250 0.274 120 -0.046 1.0000

## WT sCT - RAMP sCT 0.72500 0.274 120 2.649 0.0937

##

## time = D7:

## contrast estimate SE df t.ratio p.value

## WT Veh - RAMP Veh -0.34524 0.305 120 -1.134 0.8664

## WT Veh - WT Cagri -0.30000 0.293 120 -1.025 0.9086

## WT Veh - RAMP Cagri -0.22857 0.283 120 -0.807 0.9658

**## WT Veh - WT sCT -1.24107 0.283 120 -4.381 0.0004**

## WT Veh - RAMP sCT -0.55357 0.283 120 -1.954 0.3750

## RAMP Veh - WT Cagri 0.04524 0.305 120 0.149 1.0000

## RAMP Veh - RAMP Cagri 0.11667 0.296 120 0.395 0.9987

## RAMP Veh - WT sCT -0.89583 0.296 120 -3.030 0.0347

## RAMP Veh - RAMP sCT -0.20833 0.296 120 -0.705 0.9811

## WT Cagri - RAMP Cagri 0.07143 0.283 120 0.252 0.9999

## WT Cagri - WT sCT -0.94107 0.283 120 -3.322 0.0147

## WT Cagri - RAMP sCT -0.25357 0.283 120 -0.895 0.9470

## RAMP Cagri - WT sCT -1.01250 0.274 120 -3.699 0.0043

## RAMP Cagri - RAMP sCT -0.32500 0.274 120 -1.187 0.8421

## WT sCT - RAMP sCT 0.68750 0.274 120 2.512 0.1289

##

## time = D8:

## contrast estimate SE df t.ratio p.value

## WT Veh - RAMP Veh -0.70714 0.305 120 -2.322 0.1935

## WT Veh - WT Cagri -0.27143 0.293 120 -0.928 0.9387

## WT Veh - RAMP Cagri -0.25714 0.283 120 -0.908 0.9439

**## WT Veh - WT sCT -1.08214 0.283 120 -3.820 0.0029**

## WT Veh - RAMP sCT -0.66964 0.283 120 -2.364 0.1776

## RAMP Veh - WT Cagri 0.43571 0.305 120 1.431 0.7083

## RAMP Veh - RAMP Cagri 0.45000 0.296 120 1.522 0.6508

## RAMP Veh - WT sCT -0.37500 0.296 120 -1.268 0.8015

## RAMP Veh - RAMP sCT 0.03750 0.296 120 0.127 1.0000

## WT Cagri - RAMP Cagri 0.01429 0.283 120 0.050 1.0000

## WT Cagri - WT sCT -0.81071 0.283 120 -2.862 0.0548

## WT Cagri - RAMP sCT -0.39821 0.283 120 -1.406 0.7236

## RAMP Cagri - WT sCT -0.82500 0.274 120 -3.014 0.0362

## RAMP Cagri - RAMP sCT -0.41250 0.274 120 -1.507 0.6604

## WT sCT - RAMP sCT 0.41250 0.274 120 1.507 0.6604

##

## time = D9:

## contrast estimate SE df t.ratio p.value

## WT Veh - RAMP Veh -0.61190 0.305 120 -2.009 0.3433

## WT Veh - WT Cagri -0.40000 0.293 120 -1.367 0.7465

## WT Veh - RAMP Cagri -0.42857 0.283 120 -1.513 0.6568

**## WT Veh - WT sCT -0.90357 0.283 120 -3.189 0.0219**

## WT Veh - RAMP sCT -0.65357 0.283 120 -2.307 0.1994

## RAMP Veh - WT Cagri 0.21190 0.305 120 0.696 0.9821

## RAMP Veh - RAMP Cagri 0.18333 0.296 120 0.620 0.9894

## RAMP Veh - WT sCT -0.29167 0.296 120 -0.987 0.9214

## RAMP Veh - RAMP sCT -0.04167 0.296 120 -0.141 1.0000

## WT Cagri - RAMP Cagri -0.02857 0.283 120 -0.101 1.0000

## WT Cagri - WT sCT -0.50357 0.283 120 -1.777 0.4841

## WT Cagri - RAMP sCT -0.25357 0.283 120 -0.895 0.9470

## RAMP Cagri - WT sCT -0.47500 0.274 120 -1.735 0.5113

## RAMP Cagri - RAMP sCT -0.22500 0.274 120 -0.822 0.9629

## WT sCT - RAMP sCT 0.25000 0.274 120 0.913 0.9424

##

## time = D10:

## contrast estimate SE df t.ratio p.value

## WT Veh - RAMP Veh -0.29524 0.305 120 -0.969 0.9267

## WT Veh - WT Cagri -0.22857 0.293 120 -0.781 0.9702

## WT Veh - RAMP Cagri -0.31607 0.283 120 -1.116 0.8741

## WT Veh - WT sCT -0.77857 0.283 120 -2.748 0.0734

## WT Veh - RAMP sCT -0.70357 0.283 120 -2.483 0.1373

## RAMP Veh - WT Cagri 0.06667 0.305 120 0.219 0.9999

## RAMP Veh - RAMP Cagri -0.02083 0.296 120 -0.070 1.0000

## RAMP Veh - WT sCT -0.48333 0.296 120 -1.635 0.5773

## RAMP Veh - RAMP sCT -0.40833 0.296 120 -1.381 0.7382

## WT Cagri - RAMP Cagri -0.08750 0.283 120 -0.309 0.9996

## WT Cagri - WT sCT -0.55000 0.283 120 -1.941 0.3824

## WT Cagri - RAMP sCT -0.47500 0.283 120 -1.677 0.5499

## RAMP Cagri - WT sCT -0.46250 0.274 120 -1.690 0.5412

## RAMP Cagri - RAMP sCT -0.38750 0.274 120 -1.416 0.7174

## WT sCT - RAMP sCT 0.07500 0.274 120 0.274 0.9998

##

## time = D11:

## contrast estimate SE df t.ratio p.value

## WT Veh - RAMP Veh -0.33810 0.305 120 -1.110 0.8763

## WT Veh - WT Cagri -0.41429 0.293 120 -1.416 0.7174

## WT Veh - RAMP Cagri -0.25893 0.283 120 -0.914 0.9423

## WT Veh - WT sCT -0.57143 0.283 120 -2.017 0.3389

## WT Veh - RAMP sCT -0.85893 0.283 120 -3.032 0.0345

## RAMP Veh - WT Cagri -0.07619 0.305 120 -0.250 0.9999

## RAMP Veh - RAMP Cagri 0.07917 0.296 120 0.268 0.9998

## RAMP Veh - WT sCT -0.23333 0.296 120 -0.789 0.9689

## RAMP Veh - RAMP sCT -0.52083 0.296 120 -1.762 0.4943

## WT Cagri - RAMP Cagri 0.15536 0.283 120 0.548 0.9940

## WT Cagri - WT sCT -0.15714 0.283 120 -0.555 0.9936

## WT Cagri - RAMP sCT -0.44464 0.283 120 -1.569 0.6201

## RAMP Cagri - WT sCT -0.31250 0.274 120 -1.142 0.8629

## RAMP Cagri - RAMP sCT -0.60000 0.274 120 -2.192 0.2491

## WT sCT - RAMP sCT -0.28750 0.274 120 -1.050 0.8997

##

## time = D12:

## contrast estimate SE df t.ratio p.value

## WT Veh - RAMP Veh -0.32143 0.305 120 -1.055 0.8978

## WT Veh - WT Cagri -0.22857 0.293 120 -0.781 0.9702

## WT Veh - RAMP Cagri -0.25893 0.283 120 -0.914 0.9423

## WT Veh - WT sCT -0.60893 0.283 120 -2.149 0.2695

## WT Veh - RAMP sCT -0.73393 0.283 120 -2.591 0.1076

## RAMP Veh - WT Cagri 0.09286 0.305 120 0.305 0.9996

## RAMP Veh - RAMP Cagri 0.06250 0.296 120 0.211 0.9999

## RAMP Veh - WT sCT -0.28750 0.296 120 -0.972 0.9258

## RAMP Veh - RAMP sCT -0.41250 0.296 120 -1.395 0.7298

## WT Cagri - RAMP Cagri -0.03036 0.283 120 -0.107 1.0000

## WT Cagri - WT sCT -0.38036 0.283 120 -1.343 0.7607

## WT Cagri - RAMP sCT -0.50536 0.283 120 -1.784 0.4801

## RAMP Cagri - WT sCT -0.35000 0.274 120 -1.279 0.7960

## RAMP Cagri - RAMP sCT -0.47500 0.274 120 -1.735 0.5113

## WT sCT - RAMP sCT -0.12500 0.274 120 -0.457 0.9975

##

## time = D13:

## contrast estimate SE df t.ratio p.value

## WT Veh - RAMP Veh -0.33095 0.305 120 -1.087 0.8858

## WT Veh - WT Cagri -0.11429 0.293 120 -0.391 0.9988

## WT Veh - RAMP Cagri -0.26429 0.283 120 -0.933 0.9372

## WT Veh - WT sCT -0.63929 0.283 120 -2.256 0.2203

## WT Veh - RAMP sCT -0.73929 0.283 120 -2.609 0.1029

## RAMP Veh - WT Cagri 0.21667 0.305 120 0.711 0.9802

## RAMP Veh - RAMP Cagri 0.06667 0.296 120 0.226 0.9999

## RAMP Veh - WT sCT -0.30833 0.296 120 -1.043 0.9024

## RAMP Veh - RAMP sCT -0.40833 0.296 120 -1.381 0.7382

## WT Cagri - RAMP Cagri -0.15000 0.283 120 -0.529 0.9949

## WT Cagri - WT sCT -0.52500 0.283 120 -1.853 0.4361

## WT Cagri - RAMP sCT -0.62500 0.283 120 -2.206 0.2427

## RAMP Cagri - WT sCT -0.37500 0.274 120 -1.370 0.7447

## RAMP Cagri - RAMP sCT -0.47500 0.274 120 -1.735 0.5113

## WT sCT - RAMP sCT -0.10000 0.274 120 -0.365 0.9991

##

## time = D14:

## contrast estimate SE df t.ratio p.value

## WT Veh - RAMP Veh -0.93095 0.305 120 -3.057 0.0322

## WT Veh - WT Cagri -0.24286 0.293 120 -0.830 0.9614

## WT Veh - RAMP Cagri -0.08929 0.283 120 -0.315 0.9996

## WT Veh - WT sCT -0.57679 0.283 120 -2.036 0.3285

## WT Veh - RAMP sCT -1.02679 0.283 120 -3.624 0.0056

## RAMP Veh - WT Cagri 0.68810 0.305 120 2.259 0.2191

## RAMP Veh - RAMP Cagri 0.84167 0.296 120 2.847 0.0569

## RAMP Veh - WT sCT 0.35417 0.296 120 1.198 0.8371

## RAMP Veh - RAMP sCT -0.09583 0.296 120 -0.324 0.9995

## WT Cagri - RAMP Cagri 0.15357 0.283 120 0.542 0.9943

## WT Cagri - WT sCT -0.33393 0.283 120 -1.179 0.8462

## WT Cagri - RAMP sCT -0.78393 0.283 120 -2.767 0.0700

## RAMP Cagri - WT sCT -0.48750 0.274 120 -1.781 0.4818

## RAMP Cagri - RAMP sCT -0.93750 0.274 120 -3.425 0.0106

## WT sCT - RAMP sCT -0.45000 0.274 120 -1.644 0.5712

##

## time = D15:

## contrast estimate SE df t.ratio p.value

## WT Veh - RAMP Veh -0.65714 0.305 120 -2.158 0.2655

## WT Veh - WT Cagri -0.51429 0.293 120 -1.758 0.4969

## WT Veh - RAMP Cagri -0.46964 0.283 120 -1.658 0.5623

## WT Veh - WT sCT -0.80714 0.283 120 -2.849 0.0566

## WT Veh - RAMP sCT -0.78214 0.283 120 -2.761 0.0711

## RAMP Veh - WT Cagri 0.14286 0.305 120 0.469 0.9971

## RAMP Veh - RAMP Cagri 0.18750 0.296 120 0.634 0.9882

## RAMP Veh - WT sCT -0.15000 0.296 120 -0.507 0.9958

## RAMP Veh - RAMP sCT -0.12500 0.296 120 -0.423 0.9982

## WT Cagri - RAMP Cagri 0.04464 0.283 120 0.158 1.0000

## WT Cagri - WT sCT -0.29286 0.283 120 -1.034 0.9057

## WT Cagri - RAMP sCT -0.26786 0.283 120 -0.945 0.9337

## RAMP Cagri - WT sCT -0.33750 0.274 120 -1.233 0.8198

## RAMP Cagri - RAMP sCT -0.31250 0.274 120 -1.142 0.8629

## WT sCT - RAMP sCT 0.02500 0.274 120 0.091 1.0000

##

## time = D16:

## contrast estimate SE df t.ratio p.value

## WT Veh - RAMP Veh -0.04524 0.305 120 -0.149 1.0000

## WT Veh - WT Cagri -0.15714 0.293 120 -0.537 0.9945

## WT Veh - RAMP Cagri -0.20357 0.283 120 -0.719 0.9794

## WT Veh - WT sCT -0.62857 0.283 120 -2.219 0.2370

## WT Veh - RAMP sCT -0.75357 0.283 120 -2.660 0.0912

## RAMP Veh - WT Cagri -0.11190 0.305 120 -0.367 0.9991

## RAMP Veh - RAMP Cagri -0.15833 0.296 120 -0.536 0.9946

## RAMP Veh - WT sCT -0.58333 0.296 120 -1.973 0.3638

## RAMP Veh - RAMP sCT -0.70833 0.296 120 -2.396 0.1659

## WT Cagri - RAMP Cagri -0.04643 0.283 120 -0.164 1.0000

## WT Cagri - WT sCT -0.47143 0.283 120 -1.664 0.5581

## WT Cagri - RAMP sCT -0.59643 0.283 120 -2.105 0.2917

## RAMP Cagri - WT sCT -0.42500 0.274 120 -1.553 0.6310

## RAMP Cagri - RAMP sCT -0.55000 0.274 120 -2.009 0.3431

## WT sCT - RAMP sCT -0.12500 0.274 120 -0.457 0.9975

##

## time = D17:

## contrast estimate SE df t.ratio p.value

## WT Veh - RAMP Veh 0.24524 0.305 120 0.805 0.9661

## WT Veh - WT Cagri -0.02857 0.293 120 -0.098 1.0000

## WT Veh - RAMP Cagri 0.01607 0.283 120 0.057 1.0000

## WT Veh - WT sCT -0.52143 0.283 120 -1.840 0.4440

## WT Veh - RAMP sCT -0.74643 0.283 120 -2.635 0.0969

## RAMP Veh - WT Cagri -0.27381 0.305 120 -0.899 0.9460

## RAMP Veh - RAMP Cagri -0.22917 0.296 120 -0.775 0.9712

## RAMP Veh - WT sCT -0.76667 0.296 120 -2.593 0.1069

## RAMP Veh - RAMP sCT -0.99167 0.296 120 -3.354 0.0133

## WT Cagri - RAMP Cagri 0.04464 0.283 120 0.158 1.0000

## WT Cagri - WT sCT -0.49286 0.283 120 -1.740 0.5086

## WT Cagri - RAMP sCT -0.71786 0.283 120 -2.534 0.1226

## RAMP Cagri - WT sCT -0.53750 0.274 120 -1.964 0.3692

## RAMP Cagri - RAMP sCT -0.76250 0.274 120 -2.786 0.0667

## WT sCT - RAMP sCT -0.22500 0.274 120 -0.822 0.9629

##

## time = D18:

## contrast estimate SE df t.ratio p.value

## WT Veh - RAMP Veh -0.20952 0.305 120 -0.688 0.9830

## WT Veh - WT Cagri -0.25714 0.293 120 -0.879 0.9509

## WT Veh - RAMP Cagri -0.36786 0.283 120 -1.298 0.7854

## WT Veh - WT sCT -0.38036 0.283 120 -1.343 0.7607

## WT Veh - RAMP sCT -0.75536 0.283 120 -2.666 0.0898

## RAMP Veh - WT Cagri -0.04762 0.305 120 -0.156 1.0000

## RAMP Veh - RAMP Cagri -0.15833 0.296 120 -0.536 0.9946

## RAMP Veh - WT sCT -0.17083 0.296 120 -0.578 0.9923

## RAMP Veh - RAMP sCT -0.54583 0.296 120 -1.846 0.4404

## WT Cagri - RAMP Cagri -0.11071 0.283 120 -0.391 0.9988

## WT Cagri - WT sCT -0.12321 0.283 120 -0.435 0.9980

## WT Cagri - RAMP sCT -0.49821 0.283 120 -1.759 0.4963

## RAMP Cagri - WT sCT -0.01250 0.274 120 -0.046 1.0000

## RAMP Cagri - RAMP sCT -0.38750 0.274 120 -1.416 0.7174

## WT sCT - RAMP sCT -0.37500 0.274 120 -1.370 0.7447

##

## time = D19:

## contrast estimate SE df t.ratio p.value

## WT Veh - RAMP Veh -0.36429 0.305 120 -1.196 0.8379

## WT Veh - WT Cagri -0.40000 0.293 120 -1.367 0.7465

## WT Veh - RAMP Cagri -0.22679 0.283 120 -0.800 0.9669

## WT Veh - WT sCT -0.33929 0.283 120 -1.198 0.8372

## WT Veh - RAMP sCT -0.76429 0.283 120 -2.698 0.0832

## RAMP Veh - WT Cagri -0.03571 0.305 120 -0.117 1.0000

## RAMP Veh - RAMP Cagri 0.13750 0.296 120 0.465 0.9972

## RAMP Veh - WT sCT 0.02500 0.296 120 0.085 1.0000

## RAMP Veh - RAMP sCT -0.40000 0.296 120 -1.353 0.7547

## WT Cagri - RAMP Cagri 0.17321 0.283 120 0.611 0.9900

## WT Cagri - WT sCT 0.06071 0.283 120 0.214 0.9999

## WT Cagri - RAMP sCT -0.36429 0.283 120 -1.286 0.7922

## RAMP Cagri - WT sCT -0.11250 0.274 120 -0.411 0.9985

## RAMP Cagri - RAMP sCT -0.53750 0.274 120 -1.964 0.3692

## WT sCT - RAMP sCT -0.42500 0.274 120 -1.553 0.6310

##

## time = D20:

## contrast estimate SE df t.ratio p.value

## WT Veh - RAMP Veh -0.23095 0.305 120 -0.758 0.9738

## WT Veh - WT Cagri 0.15714 0.293 120 0.537 0.9945

## WT Veh - RAMP Cagri 0.04821 0.283 120 0.170 1.0000

## WT Veh - WT sCT -0.22679 0.283 120 -0.800 0.9669

## WT Veh - RAMP sCT -0.61429 0.283 120 -2.168 0.2604

## RAMP Veh - WT Cagri 0.38810 0.305 120 1.274 0.7984

## RAMP Veh - RAMP Cagri 0.27917 0.296 120 0.944 0.9341

## RAMP Veh - WT sCT 0.00417 0.296 120 0.014 1.0000

## RAMP Veh - RAMP sCT -0.38333 0.296 120 -1.297 0.7864

## WT Cagri - RAMP Cagri -0.10893 0.283 120 -0.384 0.9989

## WT Cagri - WT sCT -0.38393 0.283 120 -1.355 0.7534

## WT Cagri - RAMP sCT -0.77143 0.283 120 -2.723 0.0781

## RAMP Cagri - WT sCT -0.27500 0.274 120 -1.005 0.9156

## RAMP Cagri - RAMP sCT -0.66250 0.274 120 -2.420 0.1575

## WT sCT - RAMP sCT -0.38750 0.274 120 -1.416 0.7174

##

## time = D21:

## contrast estimate SE df t.ratio p.value

## WT Veh - RAMP Veh -0.21667 0.305 120 -0.711 0.9802

## WT Veh - WT Cagri 0.01429 0.293 120 0.049 1.0000

## WT Veh - RAMP Cagri -0.18750 0.283 120 -0.662 0.9857

## WT Veh - WT sCT -0.30000 0.283 120 -1.059 0.8965

## WT Veh - RAMP sCT -0.70000 0.283 120 -2.471 0.1412

## RAMP Veh - WT Cagri 0.23095 0.305 120 0.758 0.9738

## RAMP Veh - RAMP Cagri 0.02917 0.296 120 0.099 1.0000

## RAMP Veh - WT sCT -0.08333 0.296 120 -0.282 0.9998

## RAMP Veh - RAMP sCT -0.48333 0.296 120 -1.635 0.5773

## WT Cagri - RAMP Cagri -0.20179 0.283 120 -0.712 0.9801

## WT Cagri - WT sCT -0.31429 0.283 120 -1.109 0.8767

## WT Cagri - RAMP sCT -0.71429 0.283 120 -2.521 0.1262

## RAMP Cagri - WT sCT -0.11250 0.274 120 -0.411 0.9985

## RAMP Cagri - RAMP sCT -0.51250 0.274 120 -1.872 0.4241

## WT sCT - RAMP sCT -0.40000 0.274 120 -1.461 0.6893

##

## Degrees-of-freedom method: kenward-roger

## P value adjustment: tukey method for comparing a family of 6 estimates

**Figure 2b: weekly food intake**

Statistical test: Three-way Anova. Factors: genotype, treatment and week

*# running the classical 3-way ANOVA with interaction between all terms.*

## Type III Analysis of Variance Table with Satterthwaite's method

## Sum Sq Mean Sq NumDF DenDF F value Pr(>F)

## Genotype 3.398 3.3979 1 38 1.8483 0.18199

## Treatement 14.872 7.4361 2 38 4.0450 0.02555 *

## time 40.925 20.4625 2 76 11.1308 5.759e-05 ***

## Genotype:Treatement 1.852 0.9258 2 38 0.5036 0.60832

## Genotype:time 0.033 0.0165 2 76 0.0090 0.99108

## Treatement:time 52.331 13.0829 4 76 7.1166 6.382e-05 ***

## Genotype:Treatement:time 57.350 14.3376 4 76 7.7991 2.543e-05 ***

## ---

## Signif. codes: 0 '***' 0.001 '**' 0.01 '*' 0.05 '.' 0.1 ' ' 1

*# runnning pairwise comparisons on interaction model, full 15 comparisons per timepoint*

*# model: m1 <- lmer(bw ~ Genotype * Treatement * Week + (1|Mouse_ID), data = weekly_fi_long)*

emm_results <- emmeans(out[[2]], pairwise ~ Genotype * Treatement | time, adjust = "tukey")

summary(emm_results)

## $contrasts

## time = W1:

## contrast estimate SE df t.ratio p.value

## WT Veh - RAMP Veh -1.9952 1.73 49.3 -1.151 0.8572

## WT Veh - WT Cagri 2.4000 1.67 49.3 1.441 0.7020

## WT Veh - RAMP Cagri -0.7911 1.61 49.3 -0.491 0.9963

## WT Veh - WT sCT -2.0536 1.61 49.3 -1.274 0.7976

## WT Veh - RAMP sCT -0.3536 1.61 49.3 -0.219 0.9999

## RAMP Veh - WT Cagri 4.3952 1.73 49.3 2.536 0.1335

## RAMP Veh - RAMP Cagri 1.2042 1.68 49.3 0.716 0.9791

## RAMP Veh - WT sCT -0.0583 1.68 49.3 -0.035 1.0000

## RAMP Veh - RAMP sCT 1.6417 1.68 49.3 0.976 0.9234

## WT Cagri - RAMP Cagri -3.1911 1.61 49.3 -1.979 0.3686

## WT Cagri - WT sCT -4.4536 1.61 49.3 -2.762 0.0811

## WT Cagri - RAMP sCT -2.7536 1.61 49.3 -1.708 0.5333

## RAMP Cagri - WT sCT -1.2625 1.56 49.3 -0.811 0.9642

## RAMP Cagri - RAMP sCT 0.4375 1.56 49.3 0.281 0.9997

## WT sCT - RAMP sCT 1.7000 1.56 49.3 1.091 0.8825

##

## time = W2:

## contrast estimate SE df t.ratio p.value

## WT Veh - RAMP Veh -3.5214 1.73 49.3 -2.032 0.3397

## WT Veh - WT Cagri -1.8857 1.67 49.3 -1.132 0.8654

## WT Veh - RAMP Cagri -1.8589 1.61 49.3 -1.153 0.8564

**## WT Veh - WT sCT -5.1464 1.61 49.3 -3.192 0.0280**

## WT Veh - RAMP sCT -5.3714 1.61 49.3 -3.332 0.0193

## RAMP Veh - WT Cagri 1.6357 1.73 49.3 0.944 0.9329

## RAMP Veh - RAMP Cagri 1.6625 1.68 49.3 0.988 0.9196

## RAMP Veh - WT sCT -1.6250 1.68 49.3 -0.966 0.9265

## RAMP Veh - RAMP sCT -1.8500 1.68 49.3 -1.100 0.8792

## WT Cagri - RAMP Cagri 0.0268 1.61 49.3 0.017 1.0000

## WT Cagri - WT sCT -3.2607 1.61 49.3 -2.022 0.3448

## WT Cagri - RAMP sCT -3.4857 1.61 49.3 -2.162 0.2739

## RAMP Cagri - WT sCT -3.2875 1.56 49.3 -2.111 0.2989

## RAMP Cagri - RAMP sCT -3.5125 1.56 49.3 -2.255 0.2321

## WT sCT - RAMP sCT -0.2250 1.56 49.3 -0.144 1.0000

##

## time = W3:

## contrast estimate SE df t.ratio p.value

## WT Veh - RAMP Veh -1.4786 1.73 49.3 -0.853 0.9556

## WT Veh - WT Cagri -1.1857 1.67 49.3 -0.712 0.9796

## WT Veh - RAMP Cagri -1.3911 1.61 49.3 -0.863 0.9535

## WT Veh - WT sCT -3.2036 1.61 49.3 -1.987 0.3643

## WT Veh - RAMP sCT -5.1161 1.61 49.3 -3.173 0.0294

## RAMP Veh - WT Cagri 0.2929 1.73 49.3 0.169 1.0000

## RAMP Veh - RAMP Cagri 0.0875 1.68 49.3 0.052 1.0000

## RAMP Veh - WT sCT -1.7250 1.68 49.3 -1.025 0.9072

## RAMP Veh - RAMP sCT -3.6375 1.68 49.3 -2.162 0.2738

## WT Cagri - RAMP Cagri -0.2054 1.61 49.3 -0.127 1.0000

## WT Cagri - WT sCT -2.0179 1.61 49.3 -1.252 0.8091

## WT Cagri - RAMP sCT -3.9304 1.61 49.3 -2.438 0.1635

## RAMP Cagri - WT sCT -1.8125 1.56 49.3 -1.164 0.8517

## RAMP Cagri - RAMP sCT -3.7250 1.56 49.3 -2.392 0.1792

## WT sCT - RAMP sCT -1.9125 1.56 49.3 -1.228 0.8211

##

## Degrees-of-freedom method: kenward-roger

## P value adjustment: tukey method for comparing a family of 6 estimates

**Figure 2c: daily body weight**

Statistical test: Three-way Anova with repeated measure. Factors: genotype, treatment and days

*# running the classical 3-way ANOVA with interaction between all terms. (Do not use aov() here as it would ignore the (1|Mouse_ID random effect))*

anova(out[[2]]) *# choosing second model (full interaction model) from the lmer_time_result function.*

## Type III Analysis of Variance Table with Satterthwaite's method

## Sum Sq Mean Sq NumDF DenDF F value Pr(>F)

## Genotype 0.212 0.2123 1 39 0.6428 0.4276

## Treatement 0.200 0.1001 2 39 0.3032 0.7402

## time 77.841 3.7067 21 819 11.2255 <2e-16 ***

## Genotype:Treatement 0.270 0.1348 2 39 0.4083 0.6676

## Genotype:time 12.571 0.5986 21 819 1.8129 0.0142 *

## Treatement:time 62.978 1.4995 42 819 4.5411 <2e-16 ***

## Genotype:Treatement:time 142.792 3.3998 42 819 10.2961 <2e-16 ***

## ---

## Signif. codes: 0 '***' 0.001 '**' 0.01 '*' 0.05 '.' 0.1 ' ' 1

*# runnning pairwise comparisons on interaction model, full 15 comparisons per timepoint*

*# model: m1 <- lmer(bw ~ Genotype * Treatement * Week + (1|Mouse_ID), data = weekly_fi_long)*

emm_results <- emmeans(out[[2]], pairwise ~ Genotype * Treatement | time, adjust = "tukey")

summary(emm_results)

$contrasts

## time = D0:

## contrast estimate SE df t.ratio p.value

## WT Veh - RAMP Veh 0.9143 2.87 39.9 0.319 0.9995

## WT Veh - WT Cagri -0.7607 2.67 39.9 -0.285 0.9997

## WT Veh - RAMP Cagri -1.1107 2.67 39.9 -0.417 0.9983

## WT Veh - WT sCT 0.0268 2.67 39.9 0.010 1.0000

## WT Veh - RAMP sCT -2.1732 2.67 39.9 -0.815 0.9630

## RAMP Veh - WT Cagri -1.6750 2.78 39.9 -0.602 0.9903

## RAMP Veh - RAMP Cagri -2.0250 2.78 39.9 -0.728 0.9773

## RAMP Veh - WT sCT -0.8875 2.78 39.9 -0.319 0.9995

## RAMP Veh - RAMP sCT -3.0875 2.78 39.9 -1.110 0.8743

## WT Cagri - RAMP Cagri -0.3500 2.57 39.9 -0.136 1.0000

## WT Cagri - WT sCT 0.7875 2.57 39.9 0.306 0.9996

## WT Cagri - RAMP sCT -1.4125 2.57 39.9 -0.549 0.9937

## RAMP Cagri - WT sCT 1.1375 2.57 39.9 0.442 0.9977

## RAMP Cagri - RAMP sCT -1.0625 2.57 39.9 -0.413 0.9983

## WT sCT - RAMP sCT -2.2000 2.57 39.9 -0.854 0.9550

##

## time = D1:

## contrast estimate SE df t.ratio p.value

## WT Veh - RAMP Veh 1.1643 2.87 39.9 0.406 0.9985

## WT Veh - WT Cagri 0.0268 2.67 39.9 0.010 1.0000

## WT Veh - RAMP Cagri -1.0982 2.67 39.9 -0.412 0.9984

## WT Veh - WT sCT 0.6143 2.67 39.9 0.230 0.9999

## WT Veh - RAMP sCT -2.8107 2.67 39.9 -1.055 0.8961

## RAMP Veh - WT Cagri -1.1375 2.78 39.9 -0.409 0.9984

## RAMP Veh - RAMP Cagri -2.2625 2.78 39.9 -0.813 0.9634

## RAMP Veh - WT sCT -0.5500 2.78 39.9 -0.198 1.0000

## RAMP Veh - RAMP sCT -3.9750 2.78 39.9 -1.429 0.7094

## WT Cagri - RAMP Cagri -1.1250 2.57 39.9 -0.437 0.9978

## WT Cagri - WT sCT 0.5875 2.57 39.9 0.228 0.9999

## WT Cagri - RAMP sCT -2.8375 2.57 39.9 -1.102 0.8777

## RAMP Cagri - WT sCT 1.7125 2.57 39.9 0.665 0.9848

## RAMP Cagri - RAMP sCT -1.7125 2.57 39.9 -0.665 0.9848

## WT sCT - RAMP sCT -3.4250 2.57 39.9 -1.330 0.7668

##

## time = D2:

## contrast estimate SE df t.ratio p.value

## WT Veh - RAMP Veh 1.5238 2.87 39.9 0.532 0.9945

## WT Veh - WT Cagri 1.0321 2.67 39.9 0.387 0.9988

## WT Veh - RAMP Cagri -0.5679 2.67 39.9 -0.213 0.9999

## WT Veh - WT sCT 1.1696 2.67 39.9 0.439 0.9978

## WT Veh - RAMP sCT -2.2554 2.67 39.9 -0.846 0.9568

## RAMP Veh - WT Cagri -0.4917 2.78 39.9 -0.177 1.0000

## RAMP Veh - RAMP Cagri -2.0917 2.78 39.9 -0.752 0.9738

## RAMP Veh - WT sCT -0.3542 2.78 39.9 -0.127 1.0000

## RAMP Veh - RAMP sCT -3.7792 2.78 39.9 -1.359 0.7506

## WT Cagri - RAMP Cagri -1.6000 2.57 39.9 -0.621 0.9888

## WT Cagri - WT sCT 0.1375 2.57 39.9 0.053 1.0000

## WT Cagri - RAMP sCT -3.2875 2.57 39.9 -1.277 0.7957

## RAMP Cagri - WT sCT 1.7375 2.57 39.9 0.675 0.9838

## RAMP Cagri - RAMP sCT -1.6875 2.57 39.9 -0.655 0.9857

## WT sCT - RAMP sCT -3.4250 2.57 39.9 -1.330 0.7668

##

## time = D3:

## contrast estimate SE df t.ratio p.value

## WT Veh - RAMP Veh 1.2690 2.87 39.9 0.443 0.9977

## WT Veh - WT Cagri 1.0982 2.67 39.9 0.412 0.9984

## WT Veh - RAMP Cagri -1.0018 2.67 39.9 -0.376 0.9989

## WT Veh - WT sCT 0.9732 2.67 39.9 0.365 0.9991

## WT Veh - RAMP sCT -1.9018 2.67 39.9 -0.714 0.9792

## RAMP Veh - WT Cagri -0.1708 2.78 39.9 -0.061 1.0000

## RAMP Veh - RAMP Cagri -2.2708 2.78 39.9 -0.816 0.9628

## RAMP Veh - WT sCT -0.2958 2.78 39.9 -0.106 1.0000

## RAMP Veh - RAMP sCT -3.1708 2.78 39.9 -1.140 0.8616

## WT Cagri - RAMP Cagri -2.1000 2.57 39.9 -0.816 0.9630

## WT Cagri - WT sCT -0.1250 2.57 39.9 -0.049 1.0000

## WT Cagri - RAMP sCT -3.0000 2.57 39.9 -1.165 0.8505

## RAMP Cagri - WT sCT 1.9750 2.57 39.9 0.767 0.9715

## RAMP Cagri - RAMP sCT -0.9000 2.57 39.9 -0.350 0.9993

## WT sCT - RAMP sCT -2.8750 2.57 39.9 -1.117 0.8717

##

## time = D4:

## contrast estimate SE df t.ratio p.value

## WT Veh - RAMP Veh 1.3810 2.87 39.9 0.482 0.9965

## WT Veh - WT Cagri 1.5143 2.67 39.9 0.568 0.9926

## WT Veh - RAMP Cagri -1.0607 2.67 39.9 -0.398 0.9986

## WT Veh - WT sCT 0.7643 2.67 39.9 0.287 0.9997

## WT Veh - RAMP sCT -1.9982 2.67 39.9 -0.750 0.9742

## RAMP Veh - WT Cagri 0.1333 2.78 39.9 0.048 1.0000

## RAMP Veh - RAMP Cagri -2.4417 2.78 39.9 -0.878 0.9496

## RAMP Veh - WT sCT -0.6167 2.78 39.9 -0.222 0.9999

## RAMP Veh - RAMP sCT -3.3792 2.78 39.9 -1.215 0.8270

## WT Cagri - RAMP Cagri -2.5750 2.57 39.9 -1.000 0.9152

## WT Cagri - WT sCT -0.7500 2.57 39.9 -0.291 0.9997

## WT Cagri - RAMP sCT -3.5125 2.57 39.9 -1.364 0.7476

## RAMP Cagri - WT sCT 1.8250 2.57 39.9 0.709 0.9798

## RAMP Cagri - RAMP sCT -0.9375 2.57 39.9 -0.364 0.9991

## WT sCT - RAMP sCT -2.7625 2.57 39.9 -1.073 0.8892

##

## time = D5:

## contrast estimate SE df t.ratio p.value

## WT Veh - RAMP Veh 1.2548 2.87 39.9 0.438 0.9978

## WT Veh - WT Cagri 1.4964 2.67 39.9 0.561 0.9930

## WT Veh - RAMP Cagri -1.2411 2.67 39.9 -0.466 0.9971

## WT Veh - WT sCT 0.5339 2.67 39.9 0.200 1.0000

## WT Veh - RAMP sCT -1.2411 2.67 39.9 -0.466 0.9971

## RAMP Veh - WT Cagri 0.2417 2.78 39.9 0.087 1.0000

## RAMP Veh - RAMP Cagri -2.4958 2.78 39.9 -0.897 0.9449

## RAMP Veh - WT sCT -0.7208 2.78 39.9 -0.259 0.9998

## RAMP Veh - RAMP sCT -2.4958 2.78 39.9 -0.897 0.9449

## WT Cagri - RAMP Cagri -2.7375 2.57 39.9 -1.063 0.8929

## WT Cagri - WT sCT -0.9625 2.57 39.9 -0.374 0.9990

## WT Cagri - RAMP sCT -2.7375 2.57 39.9 -1.063 0.8929

## RAMP Cagri - WT sCT 1.7750 2.57 39.9 0.689 0.9821

## RAMP Cagri - RAMP sCT 0.0000 2.57 39.9 0.000 1.0000

## WT sCT - RAMP sCT -1.7750 2.57 39.9 -0.689 0.9821

##

## time = D6:

## contrast estimate SE df t.ratio p.value

## WT Veh - RAMP Veh 1.3762 2.87 39.9 0.480 0.9966

## WT Veh - WT Cagri 1.8054 2.67 39.9 0.677 0.9835

## WT Veh - RAMP Cagri -1.1946 2.67 39.9 -0.448 0.9976

## WT Veh - WT sCT 0.3554 2.67 39.9 0.133 1.0000

## WT Veh - RAMP sCT -1.1446 2.67 39.9 -0.429 0.9980

## RAMP Veh - WT Cagri 0.4292 2.78 39.9 0.154 1.0000

## RAMP Veh - RAMP Cagri -2.5708 2.78 39.9 -0.924 0.9379

## RAMP Veh - WT sCT -1.0208 2.78 39.9 -0.367 0.9991

## RAMP Veh - RAMP sCT -2.5208 2.78 39.9 -0.906 0.9426

## WT Cagri - RAMP Cagri -3.0000 2.57 39.9 -1.165 0.8505

## WT Cagri - WT sCT -1.4500 2.57 39.9 -0.563 0.9929

## WT Cagri - RAMP sCT -2.9500 2.57 39.9 -1.146 0.8592

## RAMP Cagri - WT sCT 1.5500 2.57 39.9 0.602 0.9903

## RAMP Cagri - RAMP sCT 0.0500 2.57 39.9 0.019 1.0000

## WT sCT - RAMP sCT -1.5000 2.57 39.9 -0.583 0.9916

##

## time = D7:

## contrast estimate SE df t.ratio p.value

## WT Veh - RAMP Veh 0.8548 2.87 39.9 0.298 0.9997

## WT Veh - WT Cagri 1.8464 2.67 39.9 0.693 0.9817

## WT Veh - RAMP Cagri -1.3411 2.67 39.9 -0.503 0.9958

## WT Veh - WT sCT 0.0214 2.67 39.9 0.008 1.0000

## WT Veh - RAMP sCT -0.7911 2.67 39.9 -0.297 0.9997

## RAMP Veh - WT Cagri 0.9917 2.78 39.9 0.357 0.9992

## RAMP Veh - RAMP Cagri -2.1958 2.78 39.9 -0.789 0.9677

## RAMP Veh - WT sCT -0.8333 2.78 39.9 -0.300 0.9996

## RAMP Veh - RAMP sCT -1.6458 2.78 39.9 -0.592 0.9910

## WT Cagri - RAMP Cagri -3.1875 2.57 39.9 -1.238 0.8157

## WT Cagri - WT sCT -1.8250 2.57 39.9 -0.709 0.9798

## WT Cagri - RAMP sCT -2.6375 2.57 39.9 -1.024 0.9070

## RAMP Cagri - WT sCT 1.3625 2.57 39.9 0.529 0.9946

## RAMP Cagri - RAMP sCT 0.5500 2.57 39.9 0.214 0.9999

## WT sCT - RAMP sCT -0.8125 2.57 39.9 -0.316 0.9995

##

## time = D8:

## contrast estimate SE df t.ratio p.value

## WT Veh - RAMP Veh 0.8095 2.87 39.9 0.283 0.9997

## WT Veh - WT Cagri 1.7679 2.67 39.9 0.663 0.9850

## WT Veh - RAMP Cagri -1.1696 2.67 39.9 -0.439 0.9978

## WT Veh - WT sCT -0.1446 2.67 39.9 -0.054 1.0000

## WT Veh - RAMP sCT -0.9571 2.67 39.9 -0.359 0.9992

## RAMP Veh - WT Cagri 0.9583 2.78 39.9 0.345 0.9993

## RAMP Veh - RAMP Cagri -1.9792 2.78 39.9 -0.712 0.9794

## RAMP Veh - WT sCT -0.9542 2.78 39.9 -0.343 0.9993

## RAMP Veh - RAMP sCT -1.7667 2.78 39.9 -0.635 0.9876

## WT Cagri - RAMP Cagri -2.9375 2.57 39.9 -1.141 0.8613

## WT Cagri - WT sCT -1.9125 2.57 39.9 -0.743 0.9752

## WT Cagri - RAMP sCT -2.7250 2.57 39.9 -1.058 0.8947

## RAMP Cagri - WT sCT 1.0250 2.57 39.9 0.398 0.9986

## RAMP Cagri - RAMP sCT 0.2125 2.57 39.9 0.083 1.0000

## WT sCT - RAMP sCT -0.8125 2.57 39.9 -0.316 0.9995

##

## time = D9:

## contrast estimate SE df t.ratio p.value

## WT Veh - RAMP Veh 0.5381 2.87 39.9 0.188 1.0000

## WT Veh - WT Cagri 1.7589 2.67 39.9 0.660 0.9853

## WT Veh - RAMP Cagri -1.4536 2.67 39.9 -0.545 0.9938

## WT Veh - WT sCT -0.3536 2.67 39.9 -0.133 1.0000

## WT Veh - RAMP sCT -1.0411 2.67 39.9 -0.391 0.9987

## RAMP Veh - WT Cagri 1.2208 2.78 39.9 0.439 0.9978

## RAMP Veh - RAMP Cagri -1.9917 2.78 39.9 -0.716 0.9789

## RAMP Veh - WT sCT -0.8917 2.78 39.9 -0.321 0.9995

## RAMP Veh - RAMP sCT -1.5792 2.78 39.9 -0.568 0.9926

## WT Cagri - RAMP Cagri -3.2125 2.57 39.9 -1.248 0.8107

## WT Cagri - WT sCT -2.1125 2.57 39.9 -0.820 0.9620

## WT Cagri - RAMP sCT -2.8000 2.57 39.9 -1.087 0.8835

## RAMP Cagri - WT sCT 1.1000 2.57 39.9 0.427 0.9981

## RAMP Cagri - RAMP sCT 0.4125 2.57 39.9 0.160 1.0000

## WT sCT - RAMP sCT -0.6875 2.57 39.9 -0.267 0.9998

##

## time = D10:

## contrast estimate SE df t.ratio p.value

## WT Veh - RAMP Veh 0.5357 2.87 39.9 0.187 1.0000

## WT Veh - WT Cagri 1.8857 2.67 39.9 0.707 0.9800

## WT Veh - RAMP Cagri -1.5768 2.67 39.9 -0.592 0.9910

## WT Veh - WT sCT -0.3643 2.67 39.9 -0.137 1.0000

## WT Veh - RAMP sCT -0.9893 2.67 39.9 -0.371 0.9990

## RAMP Veh - WT Cagri 1.3500 2.78 39.9 0.485 0.9964

## RAMP Veh - RAMP Cagri -2.1125 2.78 39.9 -0.760 0.9727

## RAMP Veh - WT sCT -0.9000 2.78 39.9 -0.324 0.9995

## RAMP Veh - RAMP sCT -1.5250 2.78 39.9 -0.548 0.9937

## WT Cagri - RAMP Cagri -3.4625 2.57 39.9 -1.345 0.7586

## WT Cagri - WT sCT -2.2500 2.57 39.9 -0.874 0.9506

## WT Cagri - RAMP sCT -2.8750 2.57 39.9 -1.117 0.8717

## RAMP Cagri - WT sCT 1.2125 2.57 39.9 0.471 0.9969

## RAMP Cagri - RAMP sCT 0.5875 2.57 39.9 0.228 0.9999

## WT sCT - RAMP sCT -0.6250 2.57 39.9 -0.243 0.9999

##

## time = D11:

## contrast estimate SE df t.ratio p.value

## WT Veh - RAMP Veh 0.1762 2.87 39.9 0.061 1.0000

## WT Veh - WT Cagri 1.5929 2.67 39.9 0.598 0.9906

## WT Veh - RAMP Cagri -1.6946 2.67 39.9 -0.636 0.9876

## WT Veh - WT sCT -0.3946 2.67 39.9 -0.148 1.0000

## WT Veh - RAMP sCT -1.5321 2.67 39.9 -0.575 0.9921

## RAMP Veh - WT Cagri 1.4167 2.78 39.9 0.509 0.9955

## RAMP Veh - RAMP Cagri -1.8708 2.78 39.9 -0.673 0.9840

## RAMP Veh - WT sCT -0.5708 2.78 39.9 -0.205 0.9999

## RAMP Veh - RAMP sCT -1.7083 2.78 39.9 -0.614 0.9894

## WT Cagri - RAMP Cagri -3.2875 2.57 39.9 -1.277 0.7957

## WT Cagri - WT sCT -1.9875 2.57 39.9 -0.772 0.9707

## WT Cagri - RAMP sCT -3.1250 2.57 39.9 -1.214 0.8277

## RAMP Cagri - WT sCT 1.3000 2.57 39.9 0.505 0.9957

## RAMP Cagri - RAMP sCT 0.1625 2.57 39.9 0.063 1.0000

## WT sCT - RAMP sCT -1.1375 2.57 39.9 -0.442 0.9977

##

## time = D12:

## contrast estimate SE df t.ratio p.value

## WT Veh - RAMP Veh 0.4690 2.87 39.9 0.164 1.0000

## WT Veh - WT Cagri 1.5607 2.67 39.9 0.586 0.9914

## WT Veh - RAMP Cagri -1.7518 2.67 39.9 -0.657 0.9856

## WT Veh - WT sCT -0.6393 2.67 39.9 -0.240 0.9999

## WT Veh - RAMP sCT -1.5018 2.67 39.9 -0.563 0.9928

## RAMP Veh - WT Cagri 1.0917 2.78 39.9 0.393 0.9987

## RAMP Veh - RAMP Cagri -2.2208 2.78 39.9 -0.798 0.9661

## RAMP Veh - WT sCT -1.1083 2.78 39.9 -0.398 0.9986

## RAMP Veh - RAMP sCT -1.9708 2.78 39.9 -0.709 0.9798

## WT Cagri - RAMP Cagri -3.3125 2.57 39.9 -1.286 0.7905

## WT Cagri - WT sCT -2.2000 2.57 39.9 -0.854 0.9550

## WT Cagri - RAMP sCT -3.0625 2.57 39.9 -1.189 0.8393

## RAMP Cagri - WT sCT 1.1125 2.57 39.9 0.432 0.9979

## RAMP Cagri - RAMP sCT 0.2500 2.57 39.9 0.097 1.0000

## WT sCT - RAMP sCT -0.8625 2.57 39.9 -0.335 0.9994

##

## time = D13:

## contrast estimate SE df t.ratio p.value

## WT Veh - RAMP Veh 0.2667 2.87 39.9 0.093 1.0000

## WT Veh - WT Cagri 1.6875 2.67 39.9 0.633 0.9878

## WT Veh - RAMP Cagri -1.6625 2.67 39.9 -0.624 0.9886

## WT Veh - WT sCT -0.7750 2.67 39.9 -0.291 0.9997

## WT Veh - RAMP sCT -1.5125 2.67 39.9 -0.567 0.9926

## RAMP Veh - WT Cagri 1.4208 2.78 39.9 0.511 0.9955

## RAMP Veh - RAMP Cagri -1.9292 2.78 39.9 -0.694 0.9816

## RAMP Veh - WT sCT -1.0417 2.78 39.9 -0.375 0.9990

## RAMP Veh - RAMP sCT -1.7792 2.78 39.9 -0.640 0.9872

## WT Cagri - RAMP Cagri -3.3500 2.57 39.9 -1.301 0.7827

## WT Cagri - WT sCT -2.4625 2.57 39.9 -0.956 0.9288

## WT Cagri - RAMP sCT -3.2000 2.57 39.9 -1.243 0.8132

## RAMP Cagri - WT sCT 0.8875 2.57 39.9 0.345 0.9993

## RAMP Cagri - RAMP sCT 0.1500 2.57 39.9 0.058 1.0000

## WT sCT - RAMP sCT -0.7375 2.57 39.9 -0.286 0.9997

##

## time = D14:

## contrast estimate SE df t.ratio p.value

## WT Veh - RAMP Veh 0.2762 2.87 39.9 0.096 1.0000

## WT Veh - WT Cagri 1.8679 2.67 39.9 0.701 0.9808

## WT Veh - RAMP Cagri -1.5696 2.67 39.9 -0.589 0.9912

## WT Veh - WT sCT -0.8321 2.67 39.9 -0.312 0.9996

## WT Veh - RAMP sCT -1.4571 2.67 39.9 -0.547 0.9938

## RAMP Veh - WT Cagri 1.5917 2.78 39.9 0.572 0.9923

## RAMP Veh - RAMP Cagri -1.8458 2.78 39.9 -0.664 0.9849

## RAMP Veh - WT sCT -1.1083 2.78 39.9 -0.398 0.9986

## RAMP Veh - RAMP sCT -1.7333 2.78 39.9 -0.623 0.9886

## WT Cagri - RAMP Cagri -3.4375 2.57 39.9 -1.335 0.7641

## WT Cagri - WT sCT -2.7000 2.57 39.9 -1.049 0.8983

## WT Cagri - RAMP sCT -3.3250 2.57 39.9 -1.291 0.7879

## RAMP Cagri - WT sCT 0.7375 2.57 39.9 0.286 0.9997

## RAMP Cagri - RAMP sCT 0.1125 2.57 39.9 0.044 1.0000

## WT sCT - RAMP sCT -0.6250 2.57 39.9 -0.243 0.9999

##

## time = D15:

## contrast estimate SE df t.ratio p.value

## WT Veh - RAMP Veh -0.0905 2.87 39.9 -0.032 1.0000

## WT Veh - WT Cagri 1.5554 2.67 39.9 0.584 0.9916

## WT Veh - RAMP Cagri -1.7321 2.67 39.9 -0.650 0.9863

## WT Veh - WT sCT -0.9696 2.67 39.9 -0.364 0.9991

## WT Veh - RAMP sCT -1.6821 2.67 39.9 -0.631 0.9880

## RAMP Veh - WT Cagri 1.6458 2.78 39.9 0.592 0.9910

## RAMP Veh - RAMP Cagri -1.6417 2.78 39.9 -0.590 0.9911

## RAMP Veh - WT sCT -0.8792 2.78 39.9 -0.316 0.9995

## RAMP Veh - RAMP sCT -1.5917 2.78 39.9 -0.572 0.9923

## WT Cagri - RAMP Cagri -3.2875 2.57 39.9 -1.277 0.7957

## WT Cagri - WT sCT -2.5250 2.57 39.9 -0.981 0.9214

## WT Cagri - RAMP sCT -3.2375 2.57 39.9 -1.257 0.8058

## RAMP Cagri - WT sCT 0.7625 2.57 39.9 0.296 0.9997

## RAMP Cagri - RAMP sCT 0.0500 2.57 39.9 0.019 1.0000

## WT sCT - RAMP sCT -0.7125 2.57 39.9 -0.277 0.9998

##

## time = D16:

## contrast estimate SE df t.ratio p.value

## WT Veh - RAMP Veh -0.1905 2.87 39.9 -0.066 1.0000

## WT Veh - WT Cagri 1.7179 2.67 39.9 0.645 0.9868

## WT Veh - RAMP Cagri -2.0071 2.67 39.9 -0.753 0.9737

## WT Veh - WT sCT -1.3446 2.67 39.9 -0.504 0.9957

## WT Veh - RAMP sCT -2.2696 2.67 39.9 -0.852 0.9556

## RAMP Veh - WT Cagri 1.9083 2.78 39.9 0.686 0.9825

## RAMP Veh - RAMP Cagri -1.8167 2.78 39.9 -0.653 0.9860

## RAMP Veh - WT sCT -1.1542 2.78 39.9 -0.415 0.9983

## RAMP Veh - RAMP sCT -2.0792 2.78 39.9 -0.748 0.9745

## WT Cagri - RAMP Cagri -3.7250 2.57 39.9 -1.447 0.6989

## WT Cagri - WT sCT -3.0625 2.57 39.9 -1.189 0.8393

## WT Cagri - RAMP sCT -3.9875 2.57 39.9 -1.549 0.6359

## RAMP Cagri - WT sCT 0.6625 2.57 39.9 0.257 0.9998

## RAMP Cagri - RAMP sCT -0.2625 2.57 39.9 -0.102 1.0000

## WT sCT - RAMP sCT -0.9250 2.57 39.9 -0.359 0.9992

##

## time = D17:

## contrast estimate SE df t.ratio p.value

## WT Veh - RAMP Veh 0.1905 2.87 39.9 0.066 1.0000

## WT Veh - WT Cagri 1.7196 2.67 39.9 0.645 0.9867

## WT Veh - RAMP Cagri -1.5929 2.67 39.9 -0.598 0.9906

## WT Veh - WT sCT -1.5054 2.67 39.9 -0.565 0.9928

## WT Veh - RAMP sCT -2.2054 2.67 39.9 -0.827 0.9606

## RAMP Veh - WT Cagri 1.5292 2.78 39.9 0.550 0.9936

## RAMP Veh - RAMP Cagri -1.7833 2.78 39.9 -0.641 0.9871

## RAMP Veh - WT sCT -1.6958 2.78 39.9 -0.610 0.9897

## RAMP Veh - RAMP sCT -2.3958 2.78 39.9 -0.861 0.9534

## WT Cagri - RAMP Cagri -3.3125 2.57 39.9 -1.286 0.7905

## WT Cagri - WT sCT -3.2250 2.57 39.9 -1.252 0.8083

## WT Cagri - RAMP sCT -3.9250 2.57 39.9 -1.524 0.6511

## RAMP Cagri - WT sCT 0.0875 2.57 39.9 0.034 1.0000

## RAMP Cagri - RAMP sCT -0.6125 2.57 39.9 -0.238 0.9999

## WT sCT - RAMP sCT -0.7000 2.57 39.9 -0.272 0.9998

##

## time = D18:

## contrast estimate SE df t.ratio p.value

## WT Veh - RAMP Veh 0.1381 2.87 39.9 0.048 1.0000

## WT Veh - WT Cagri 1.7589 2.67 39.9 0.660 0.9853

## WT Veh - RAMP Cagri -1.9161 2.67 39.9 -0.719 0.9785

## WT Veh - WT sCT -1.3536 2.67 39.9 -0.508 0.9956

## WT Veh - RAMP sCT -2.2911 2.67 39.9 -0.860 0.9538

## RAMP Veh - WT Cagri 1.6208 2.78 39.9 0.583 0.9916

## RAMP Veh - RAMP Cagri -2.0542 2.78 39.9 -0.739 0.9758

## RAMP Veh - WT sCT -1.4917 2.78 39.9 -0.536 0.9943

## RAMP Veh - RAMP sCT -2.4292 2.78 39.9 -0.873 0.9507

## WT Cagri - RAMP Cagri -3.6750 2.57 39.9 -1.427 0.7106

## WT Cagri - WT sCT -3.1125 2.57 39.9 -1.209 0.8300

## WT Cagri - RAMP sCT -4.0500 2.57 39.9 -1.573 0.6206

## RAMP Cagri - WT sCT 0.5625 2.57 39.9 0.218 0.9999

## RAMP Cagri - RAMP sCT -0.3750 2.57 39.9 -0.146 1.0000

## WT sCT - RAMP sCT -0.9375 2.57 39.9 -0.364 0.9991

##

## time = D19:

## contrast estimate SE df t.ratio p.value

## WT Veh - RAMP Veh -0.1548 2.87 39.9 -0.054 1.0000

## WT Veh - WT Cagri 1.2286 2.67 39.9 0.461 0.9972

## WT Veh - RAMP Cagri -2.0214 2.67 39.9 -0.758 0.9728

## WT Veh - WT sCT -1.4339 2.67 39.9 -0.538 0.9942

## WT Veh - RAMP sCT -2.6339 2.67 39.9 -0.988 0.9190

## RAMP Veh - WT Cagri 1.3833 2.78 39.9 0.497 0.9960

## RAMP Veh - RAMP Cagri -1.8667 2.78 39.9 -0.671 0.9841

## RAMP Veh - WT sCT -1.2792 2.78 39.9 -0.460 0.9972

## RAMP Veh - RAMP sCT -2.4792 2.78 39.9 -0.891 0.9464

## WT Cagri - RAMP Cagri -3.2500 2.57 39.9 -1.262 0.8033

## WT Cagri - WT sCT -2.6625 2.57 39.9 -1.034 0.9036

## WT Cagri - RAMP sCT -3.8625 2.57 39.9 -1.500 0.6662

## RAMP Cagri - WT sCT 0.5875 2.57 39.9 0.228 0.9999

## RAMP Cagri - RAMP sCT -0.6125 2.57 39.9 -0.238 0.9999

## WT sCT - RAMP sCT -1.2000 2.57 39.9 -0.466 0.9971

##

## time = D20:

## contrast estimate SE df t.ratio p.value

## WT Veh - RAMP Veh 0.1643 2.87 39.9 0.057 1.0000

## WT Veh - WT Cagri 1.5018 2.67 39.9 0.563 0.9928

## WT Veh - RAMP Cagri -1.4982 2.67 39.9 -0.562 0.9929

## WT Veh - WT sCT -1.3732 2.67 39.9 -0.515 0.9953

## WT Veh - RAMP sCT -2.1482 2.67 39.9 -0.806 0.9648

## RAMP Veh - WT Cagri 1.3375 2.78 39.9 0.481 0.9966

## RAMP Veh - RAMP Cagri -1.6625 2.78 39.9 -0.598 0.9906

## RAMP Veh - WT sCT -1.5375 2.78 39.9 -0.553 0.9934

## RAMP Veh - RAMP sCT -2.3125 2.78 39.9 -0.831 0.9598

## WT Cagri - RAMP Cagri -3.0000 2.57 39.9 -1.165 0.8505

## WT Cagri - WT sCT -2.8750 2.57 39.9 -1.117 0.8717

## WT Cagri - RAMP sCT -3.6500 2.57 39.9 -1.417 0.7164

## RAMP Cagri - WT sCT 0.1250 2.57 39.9 0.049 1.0000

## RAMP Cagri - RAMP sCT -0.6500 2.57 39.9 -0.252 0.9998

## WT sCT - RAMP sCT -0.7750 2.57 39.9 -0.301 0.9996

##

## time = D21:

## contrast estimate SE df t.ratio p.value

## WT Veh - RAMP Veh -0.0476 2.87 39.9 -0.017 1.0000

## WT Veh - WT Cagri 1.5107 2.67 39.9 0.567 0.9926

## WT Veh - RAMP Cagri -1.8518 2.67 39.9 -0.695 0.9815

## WT Veh - WT sCT -1.7018 2.67 39.9 -0.638 0.9873

## WT Veh - RAMP sCT -2.9643 2.67 39.9 -1.112 0.8735

## RAMP Veh - WT Cagri 1.5583 2.78 39.9 0.560 0.9930

## RAMP Veh - RAMP Cagri -1.8042 2.78 39.9 -0.649 0.9864

## RAMP Veh - WT sCT -1.6542 2.78 39.9 -0.595 0.9908

## RAMP Veh - RAMP sCT -2.9167 2.78 39.9 -1.049 0.8983

## WT Cagri - RAMP Cagri -3.3625 2.57 39.9 -1.306 0.7801

## WT Cagri - WT sCT -3.2125 2.57 39.9 -1.248 0.8107

## WT Cagri - RAMP sCT -4.4750 2.57 39.9 -1.738 0.5159

## RAMP Cagri - WT sCT 0.1500 2.57 39.9 0.058 1.0000

## RAMP Cagri - RAMP sCT -1.1125 2.57 39.9 -0.432 0.9979

## WT sCT - RAMP sCT -1.2625 2.57 39.9 -0.490 0.9963

##

## Degrees-of-freedom method: kenward-roger

## P value adjustment: tukey method for comparing a family of 6 estimates

**Figure 2d: cumulative daily body weight gain**

Statistical test: Three-way Anova with repeated measure. Factors: genotype, treatment and days.

*# running the classical 3-way ANOVA with interaction between all terms. (Do not use aov() here as it would ignore the (1|Mouse_ID random effect))*

anova(out[[2]]) *# choosing second model (full interaction model) from the lmer_time_result function.*

## Type III Analysis of Variance Table with Satterthwaite's method

## Sum Sq Mean Sq NumDF DenDF F value Pr(>F)

## Genotype 2.463 2.4629 1 39 7.4586 0.009431 **

## Treatement 4.932 2.4662 2 39 7.4686 0.001795 **

## time 77.841 3.7067 21 819 11.2255 < 2.2e-16 ***

## Genotype:Treatement 10.741 5.3704 2 39 16.2638 7.304e-06 ***

## Genotype:time 12.571 0.5986 21 819 1.8129 0.014196 *

## Treatement:time 62.978 1.4995 42 819 4.5411 < 2.2e-16 ***

## Genotype:Treatement:time 142.792 3.3998 42 819 10.2961 < 2.2e-16 ***

## ---

## Signif. codes: 0 '***' 0.001 '**' 0.01 '*' 0.05 '.' 0.1 ' ' 1

*# runnning pairwise comparisons on interaction model, full 15 comparisons per timepoint*

*# model: m1 <- lmer(bw ~ Genotype * Treatement * Week + (1|Mouse_ID), data = weekly_fi_long)*

emm_results <- emmeans(out[[2]], pairwise ~ Genotype * Treatement | time, adjust = "tukey")

summary(emm_results)

## $contrasts

## time = D0:

## contrast estimate SE df t.ratio p.value

## WT Veh - RAMP Veh 0.00000 0.561 81.2 0.000 1.0000

## WT Veh - WT Cagri 0.00000 0.522 81.2 0.000 1.0000

## WT Veh - RAMP Cagri 0.00000 0.522 81.2 0.000 1.0000

## WT Veh - WT sCT 0.00000 0.522 81.2 0.000 1.0000

## WT Veh - RAMP sCT 0.00000 0.522 81.2 0.000 1.0000

## RAMP Veh - WT Cagri 0.00000 0.544 81.2 0.000 1.0000

## RAMP Veh - RAMP Cagri 0.00000 0.544 81.2 0.000 1.0000

## RAMP Veh - WT sCT 0.00000 0.544 81.2 0.000 1.0000

## RAMP Veh - RAMP sCT 0.00000 0.544 81.2 0.000 1.0000

## WT Cagri - RAMP Cagri 0.00000 0.504 81.2 0.000 1.0000

## WT Cagri - WT sCT 0.00000 0.504 81.2 0.000 1.0000

## WT Cagri - RAMP sCT 0.00000 0.504 81.2 0.000 1.0000

## RAMP Cagri - WT sCT 0.00000 0.504 81.2 0.000 1.0000

## RAMP Cagri - RAMP sCT 0.00000 0.504 81.2 0.000 1.0000

## WT sCT - RAMP sCT 0.00000 0.504 81.2 0.000 1.0000

##

## time = D1:

## contrast estimate SE df t.ratio p.value

## WT Veh - RAMP Veh 0.25000 0.561 81.2 0.446 0.9977

## WT Veh - WT Cagri 0.78750 0.522 81.2 1.510 0.6592

## WT Veh - RAMP Cagri 0.01250 0.522 81.2 0.024 1.0000

## WT Veh - WT sCT 0.58750 0.522 81.2 1.126 0.8691

## WT Veh - RAMP sCT -0.63750 0.522 81.2 -1.222 0.8249

## RAMP Veh - WT Cagri 0.53750 0.544 81.2 0.987 0.9207

## RAMP Veh - RAMP Cagri -0.23750 0.544 81.2 -0.436 0.9979

## RAMP Veh - WT sCT 0.33750 0.544 81.2 0.620 0.9893

## RAMP Veh - RAMP sCT -0.88750 0.544 81.2 -1.630 0.5812

## WT Cagri - RAMP Cagri -0.77500 0.504 81.2 -1.538 0.6412

## WT Cagri - WT sCT -0.20000 0.504 81.2 -0.397 0.9987

## WT Cagri - RAMP sCT -1.42500 0.504 81.2 -2.827 0.0632

## RAMP Cagri - WT sCT 0.57500 0.504 81.2 1.141 0.8628

## RAMP Cagri - RAMP sCT -0.65000 0.504 81.2 -1.290 0.7898

## WT sCT - RAMP sCT -1.22500 0.504 81.2 -2.431 0.1581

##

## time = D2:

## contrast estimate SE df t.ratio p.value

## WT Veh - RAMP Veh 0.60952 0.561 81.2 1.087 0.8853

**## WT Veh - WT Cagri 1.79286 0.522 81.2 3.437 0.0116**

## WT Veh - RAMP Cagri 0.54286 0.522 81.2 1.041 0.9027

## WT Veh - WT sCT 1.14286 0.522 81.2 2.191 0.2535

## WT Veh - RAMP sCT -0.08214 0.522 81.2 -0.157 1.0000

## RAMP Veh - WT Cagri 1.18333 0.544 81.2 2.174 0.2614

## RAMP Veh - RAMP Cagri -0.06667 0.544 81.2 -0.122 1.0000

## RAMP Veh - WT sCT 0.53333 0.544 81.2 0.980 0.9231

## RAMP Veh - RAMP sCT -0.69167 0.544 81.2 -1.271 0.8000

## WT Cagri - RAMP Cagri -1.25000 0.504 81.2 -2.480 0.1423

## WT Cagri - WT sCT -0.65000 0.504 81.2 -1.290 0.7898

## WT Cagri - RAMP sCT -1.87500 0.504 81.2 -3.720 0.0048

## RAMP Cagri - WT sCT 0.60000 0.504 81.2 1.190 0.8402

## RAMP Cagri - RAMP sCT -0.62500 0.504 81.2 -1.240 0.8158

## WT sCT - RAMP sCT -1.22500 0.504 81.2 -2.431 0.1581

##

## time = D3:

## contrast estimate SE df t.ratio p.value

## WT Veh - RAMP Veh 0.35476 0.561 81.2 0.633 0.9882

**## WT Veh - WT Cagri 1.85893 0.522 81.2 3.563 0.0078**

## WT Veh - RAMP Cagri 0.10893 0.522 81.2 0.209 0.9999

## WT Veh - WT sCT 0.94643 0.522 81.2 1.814 0.4626

## WT Veh - RAMP sCT 0.27143 0.522 81.2 0.520 0.9952

## RAMP Veh - WT Cagri 1.50417 0.544 81.2 2.763 0.0741

## RAMP Veh - RAMP Cagri -0.24583 0.544 81.2 -0.452 0.9976

## RAMP Veh - WT sCT 0.59167 0.544 81.2 1.087 0.8853

## RAMP Veh - RAMP sCT -0.08333 0.544 81.2 -0.153 1.0000

**## WT Cagri - RAMP Cagri -1.75000 0.504 81.2 -3.472 0.0104**

## WT Cagri - WT sCT -0.91250 0.504 81.2 -1.810 0.4649

## WT Cagri - RAMP sCT -1.58750 0.504 81.2 -3.150 0.0268

## RAMP Cagri - WT sCT 0.83750 0.504 81.2 1.662 0.5608

## RAMP Cagri - RAMP sCT 0.16250 0.504 81.2 0.322 0.9995

## WT sCT - RAMP sCT -0.67500 0.504 81.2 -1.339 0.7623

##

## time = D4:

## contrast estimate SE df t.ratio p.value

## WT Veh - RAMP Veh 0.46667 0.561 81.2 0.832 0.9607

## WT Veh - WT Cagri 2.27500 0.522 81.2 4.361 0.0005

## WT Veh - RAMP Cagri 0.05000 0.522 81.2 0.096 1.0000

## WT Veh - WT sCT 0.73750 0.522 81.2 1.414 0.7187

## WT Veh - RAMP sCT 0.17500 0.522 81.2 0.335 0.9994

**## RAMP Veh - WT Cagri 1.80833 0.544 81.2 3.322 0.0163**

## RAMP Veh - RAMP Cagri -0.41667 0.544 81.2 -0.765 0.9725

## RAMP Veh - WT sCT 0.27083 0.544 81.2 0.498 0.9961

## RAMP Veh - RAMP sCT -0.29167 0.544 81.2 -0.536 0.9945

**## WT Cagri - RAMP Cagri -2.22500 0.504 81.2 -4.415 0.0004**

**## WT Cagri - WT sCT -1.53750 0.504 81.2 -3.051 0.0352**

## WT Cagri - RAMP sCT -2.10000 0.504 81.2 -4.167 0.0011

## RAMP Cagri - WT sCT 0.68750 0.504 81.2 1.364 0.7481

## RAMP Cagri - RAMP sCT 0.12500 0.504 81.2 0.248 0.9999

## WT sCT - RAMP sCT -0.56250 0.504 81.2 -1.116 0.8734

##

## time = D5:

## contrast estimate SE df t.ratio p.value

## WT Veh - RAMP Veh 0.34048 0.561 81.2 0.607 0.9902

**## WT Veh - WT Cagri 2.25714 0.522 81.2 4.327 0.0006**

## WT Veh - RAMP Cagri -0.13036 0.522 81.2 -0.250 0.9999

## WT Veh - WT sCT 0.50714 0.522 81.2 0.972 0.9255

## WT Veh - RAMP sCT 0.93214 0.522 81.2 1.787 0.4799

## RAMP Veh - WT Cagri 1.91667 0.544 81.2 3.521 0.0090

## RAMP Veh - RAMP Cagri -0.47083 0.544 81.2 -0.865 0.9537

## RAMP Veh - WT sCT 0.16667 0.544 81.2 0.306 0.9996

## RAMP Veh - RAMP sCT 0.59167 0.544 81.2 1.087 0.8853

**## WT Cagri - RAMP Cagri -2.38750 0.504 81.2 -4.737 0.0001**

**## WT Cagri - WT sCT -1.75000 0.504 81.2 -3.472 0.0104**

## WT Cagri - RAMP sCT -1.32500 0.504 81.2 -2.629 0.1020

## RAMP Cagri - WT sCT 0.63750 0.504 81.2 1.265 0.8030

## RAMP Cagri - RAMP sCT 1.06250 0.504 81.2 2.108 0.2936

## WT sCT - RAMP sCT 0.42500 0.504 81.2 0.843 0.9584

##

## time = D6:

## contrast estimate SE df t.ratio p.value

## WT Veh - RAMP Veh 0.46191 0.561 81.2 0.824 0.9623

**## WT Veh - WT Cagri 2.56607 0.522 81.2 4.919 0.0001**

## WT Veh - RAMP Cagri -0.08393 0.522 81.2 -0.161 1.0000

## WT Veh - WT sCT 0.32857 0.522 81.2 0.630 0.9885

## WT Veh - RAMP sCT 1.02857 0.522 81.2 1.972 0.3675

## RAMP Veh - WT Cagri 2.10417 0.544 81.2 3.865 0.0030

## RAMP Veh - RAMP Cagri -0.54583 0.544 81.2 -1.003 0.9158

## RAMP Veh - WT sCT -0.13333 0.544 81.2 -0.245 0.9999

## RAMP Veh - RAMP sCT 0.56667 0.544 81.2 1.041 0.9026

**## WT Cagri - RAMP Cagri -2.65000 0.504 81.2 -5.258 <.0001**

**## WT Cagri - WT sCT -2.23750 0.504 81.2 -4.439 0.0004**

## WT Cagri - RAMP sCT -1.53750 0.504 81.2 -3.051 0.0352

## RAMP Cagri - WT sCT 0.41250 0.504 81.2 0.818 0.9633

## RAMP Cagri - RAMP sCT 1.11250 0.504 81.2 2.207 0.2458

## WT sCT - RAMP sCT 0.70000 0.504 81.2 1.389 0.7335

##

## time = D7:

## contrast estimate SE df t.ratio p.value

## WT Veh - RAMP Veh -0.05952 0.561 81.2 -0.106 1.0000

**## WT Veh - WT Cagri 2.60714 0.522 81.2 4.997 <.0001**

## WT Veh - RAMP Cagri -0.23036 0.522 81.2 -0.442 0.9978

## WT Veh - WT sCT -0.00536 0.522 81.2 -0.010 1.0000

## WT Veh - RAMP sCT 1.38214 0.522 81.2 2.649 0.0973

## RAMP Veh - WT Cagri 2.66667 0.544 81.2 4.898 0.0001

## RAMP Veh - RAMP Cagri -0.17083 0.544 81.2 -0.314 0.9996

## RAMP Veh - WT sCT 0.05417 0.544 81.2 0.100 1.0000

## RAMP Veh - RAMP sCT 1.44167 0.544 81.2 2.648 0.0975

**## WT Cagri - RAMP Cagri -2.83750 0.504 81.2 -5.630 <.0001**

**## WT Cagri - WT sCT -2.61250 0.504 81.2 -5.183 <.0001**

## WT Cagri - RAMP sCT -1.22500 0.504 81.2 -2.431 0.1581

## RAMP Cagri - WT sCT 0.22500 0.504 81.2 0.446 0.9977

## RAMP Cagri - RAMP sCT 1.61250 0.504 81.2 3.199 0.0233

## WT sCT - RAMP sCT 1.38750 0.504 81.2 2.753 0.0760

##

## time = D8:

## contrast estimate SE df t.ratio p.value

## WT Veh - RAMP Veh -0.10476 0.561 81.2 -0.187 1.0000

**## WT Veh - WT Cagri 2.52857 0.522 81.2 4.847 0.0001**

## WT Veh - RAMP Cagri -0.05893 0.522 81.2 -0.113 1.0000

## WT Veh - WT sCT -0.17143 0.522 81.2 -0.329 0.9995

## WT Veh - RAMP sCT 1.21607 0.522 81.2 2.331 0.1939

## RAMP Veh - WT Cagri 2.63333 0.544 81.2 4.837 0.0001

## RAMP Veh - RAMP Cagri 0.04583 0.544 81.2 0.084 1.0000

## RAMP Veh - WT sCT -0.06667 0.544 81.2 -0.122 1.0000

## RAMP Veh - RAMP sCT 1.32083 0.544 81.2 2.426 0.1595

**## WT Cagri - RAMP Cagri -2.58750 0.504 81.2 -5.134 <.0001**

**## WT Cagri - WT sCT -2.70000 0.504 81.2 -5.357 <.0001**

## WT Cagri - RAMP sCT -1.31250 0.504 81.2 -2.604 0.1080

## RAMP Cagri - WT sCT -0.11250 0.504 81.2 -0.223 0.9999

## RAMP Cagri - RAMP sCT 1.27500 0.504 81.2 2.530 0.1277

## WT sCT - RAMP sCT 1.38750 0.504 81.2 2.753 0.0760

##

## time = D9:

## contrast estimate SE df t.ratio p.value

## WT Veh - RAMP Veh -0.37619 0.561 81.2 -0.671 0.9847

**## WT Veh - WT Cagri 2.51964 0.522 81.2 4.830 0.0001**

## WT Veh - RAMP Cagri -0.34286 0.522 81.2 -0.657 0.9860

## WT Veh - WT sCT -0.38036 0.522 81.2 -0.729 0.9778

## WT Veh - RAMP sCT 1.13214 0.522 81.2 2.170 0.2631

## RAMP Veh - WT Cagri 2.89583 0.544 81.2 5.319 <.0001

## RAMP Veh - RAMP Cagri 0.03333 0.544 81.2 0.061 1.0000

## RAMP Veh - WT sCT -0.00417 0.544 81.2 -0.008 1.0000

## RAMP Veh - RAMP sCT 1.50833 0.544 81.2 2.771 0.0727

**## WT Cagri - RAMP Cagri -2.86250 0.504 81.2 -5.679 <.0001**

**## WT Cagri - WT sCT -2.90000 0.504 81.2 -5.754 <.0001**

## WT Cagri - RAMP sCT -1.38750 0.504 81.2 -2.753 0.0760

## RAMP Cagri - WT sCT -0.03750 0.504 81.2 -0.074 1.0000

**## RAMP Cagri - RAMP sCT 1.47500 0.504 81.2 2.927 0.0490**

## WT sCT - RAMP sCT 1.51250 0.504 81.2 3.001 0.0403

##

## time = D10:

## contrast estimate SE df t.ratio p.value

## WT Veh - RAMP Veh -0.37857 0.561 81.2 -0.675 0.9842

**## WT Veh - WT Cagri 2.64643 0.522 81.2 5.073 <.0001**

## WT Veh - RAMP Cagri -0.46607 0.522 81.2 -0.893 0.9471

## WT Veh - WT sCT -0.39107 0.522 81.2 -0.750 0.9749

## WT Veh - RAMP sCT 1.18393 0.522 81.2 2.269 0.2187

## RAMP Veh - WT Cagri 3.02500 0.544 81.2 5.557 <.0001

## RAMP Veh - RAMP Cagri -0.08750 0.544 81.2 -0.161 1.0000

## RAMP Veh - WT sCT -0.01250 0.544 81.2 -0.023 1.0000

## RAMP Veh - RAMP sCT 1.56250 0.544 81.2 2.870 0.0567

**## WT Cagri - RAMP Cagri -3.11250 0.504 81.2 -6.176 <.0001**

**## WT Cagri - WT sCT -3.03750 0.504 81.2 -6.027 <.0001**

## WT Cagri - RAMP sCT -1.46250 0.504 81.2 -2.902 0.0523

## RAMP Cagri - WT sCT 0.07500 0.504 81.2 0.149 1.0000

**## RAMP Cagri - RAMP sCT 1.65000 0.504 81.2 3.274 0.0188**

## WT sCT - RAMP sCT 1.57500 0.504 81.2 3.125 0.0287

##

## time = D11:

## contrast estimate SE df t.ratio p.value

## WT Veh - RAMP Veh -0.73809 0.561 81.2 -1.316 0.7753

**## WT Veh - WT Cagri 2.35357 0.522 81.2 4.511 0.0003**

## WT Veh - RAMP Cagri -0.58393 0.522 81.2 -1.119 0.8720

## WT Veh - WT sCT -0.42143 0.522 81.2 -0.808 0.9653

## WT Veh - RAMP sCT 0.64107 0.522 81.2 1.229 0.8215

## RAMP Veh - WT Cagri 3.09167 0.544 81.2 5.679 <.0001

## RAMP Veh - RAMP Cagri 0.15417 0.544 81.2 0.283 0.9997

## RAMP Veh - WT sCT 0.31667 0.544 81.2 0.582 0.9920

## RAMP Veh - RAMP sCT 1.37917 0.544 81.2 2.533 0.1266

**## WT Cagri - RAMP Cagri -2.93750 0.504 81.2 -5.828 <.0001**

**## WT Cagri - WT sCT -2.77500 0.504 81.2 -5.506 <.0001**

## WT Cagri - RAMP sCT -1.71250 0.504 81.2 -3.398 0.0130

## RAMP Cagri - WT sCT 0.16250 0.504 81.2 0.322 0.9995

## RAMP Cagri - RAMP sCT 1.22500 0.504 81.2 2.431 0.1581

## WT sCT - RAMP sCT 1.06250 0.504 81.2 2.108 0.2936

##

## time = D12:

## contrast estimate SE df t.ratio p.value

## WT Veh - RAMP Veh -0.44524 0.561 81.2 -0.794 0.9678

**## WT Veh - WT Cagri 2.32143 0.522 81.2 4.450 0.0004**

## WT Veh - RAMP Cagri -0.64107 0.522 81.2 -1.229 0.8215

## WT Veh - WT sCT -0.66607 0.522 81.2 -1.277 0.7967

## WT Veh - RAMP sCT 0.67143 0.522 81.2 1.287 0.7912

## RAMP Veh - WT Cagri 2.76667 0.544 81.2 5.082 <.0001

## RAMP Veh - RAMP Cagri -0.19583 0.544 81.2 -0.360 0.9992

## RAMP Veh - WT sCT -0.22083 0.544 81.2 -0.406 0.9985

## RAMP Veh - RAMP sCT 1.11667 0.544 81.2 2.051 0.3233

**## WT Cagri - RAMP Cagri -2.96250 0.504 81.2 -5.878 <.0001**

**## WT Cagri - WT sCT -2.98750 0.504 81.2 -5.928 <.0001**

## WT Cagri - RAMP sCT -1.65000 0.504 81.2 -3.274 0.0188

## RAMP Cagri - WT sCT -0.02500 0.504 81.2 -0.050 1.0000

## RAMP Cagri - RAMP sCT 1.31250 0.504 81.2 2.604 0.1080

## WT sCT - RAMP sCT 1.33750 0.504 81.2 2.654 0.0963

##

## time = D13:

## contrast estimate SE df t.ratio p.value

## WT Veh - RAMP Veh -0.64762 0.561 81.2 -1.155 0.8566

**## WT Veh - WT Cagri 2.44821 0.522 81.2 4.693 0.0002**

## WT Veh - RAMP Cagri -0.55179 0.522 81.2 -1.058 0.8965

## WT Veh - WT sCT -0.80179 0.522 81.2 -1.537 0.6417

## WT Veh - RAMP sCT 0.66071 0.522 81.2 1.266 0.8022

## RAMP Veh - WT Cagri 3.09583 0.544 81.2 5.687 <.0001

## RAMP Veh - RAMP Cagri 0.09583 0.544 81.2 0.176 1.0000

## RAMP Veh - WT sCT -0.15417 0.544 81.2 -0.283 0.9997

## RAMP Veh - RAMP sCT 1.30833 0.544 81.2 2.403 0.1674

**## WT Cagri - RAMP Cagri -3.00000 0.504 81.2 -5.952 <.0001**

**## WT Cagri - WT sCT -3.25000 0.504 81.2 -6.448 <.0001**

## WT Cagri - RAMP sCT -1.78750 0.504 81.2 -3.547 0.0083

## RAMP Cagri - WT sCT -0.25000 0.504 81.2 -0.496 0.9962

## RAMP Cagri - RAMP sCT 1.21250 0.504 81.2 2.406 0.1665

## WT sCT - RAMP sCT 1.46250 0.504 81.2 2.902 0.0523

##

## time = D14:

## contrast estimate SE df t.ratio p.value

## WT Veh - RAMP Veh -0.63809 0.561 81.2 -1.138 0.8641

**## WT Veh - WT Cagri 2.62857 0.522 81.2 5.039 <.0001**

## WT Veh - RAMP Cagri -0.45893 0.522 81.2 -0.880 0.9503

## WT Veh - WT sCT -0.85893 0.522 81.2 -1.646 0.5707

## WT Veh - RAMP sCT 0.71607 0.522 81.2 1.373 0.7431

## RAMP Veh - WT Cagri 3.26667 0.544 81.2 6.001 <.0001

## RAMP Veh - RAMP Cagri 0.17917 0.544 81.2 0.329 0.9995

## RAMP Veh - WT sCT -0.22083 0.544 81.2 -0.406 0.9985

## RAMP Veh - RAMP sCT 1.35417 0.544 81.2 2.487 0.1400

**## WT Cagri - RAMP Cagri -3.08750 0.504 81.2 -6.126 <.0001**

**## WT Cagri - WT sCT -3.48750 0.504 81.2 -6.920 <.0001**

## WT Cagri - RAMP sCT -1.91250 0.504 81.2 -3.795 0.0037

## RAMP Cagri - WT sCT -0.40000 0.504 81.2 -0.794 0.9678

## RAMP Cagri - RAMP sCT 1.17500 0.504 81.2 2.331 0.1937

## WT sCT - RAMP sCT 1.57500 0.504 81.2 3.125 0.0287

##

## time = D15:

## contrast estimate SE df t.ratio p.value

## WT Veh - RAMP Veh -1.00476 0.561 81.2 -1.792 0.4768

**## WT Veh - WT Cagri 2.31607 0.522 81.2 4.440 0.0004**

## WT Veh - RAMP Cagri -0.62143 0.522 81.2 -1.191 0.8398

## WT Veh - WT sCT -0.99643 0.522 81.2 -1.910 0.4037

## WT Veh - RAMP sCT 0.49107 0.522 81.2 0.941 0.9345

## RAMP Veh - WT Cagri 3.32083 0.544 81.2 6.100 <.0001

## RAMP Veh - RAMP Cagri 0.38333 0.544 81.2 0.704 0.9809

## RAMP Veh - WT sCT 0.00833 0.544 81.2 0.015 1.0000

## RAMP Veh - RAMP sCT 1.49583 0.544 81.2 2.748 0.0769

**## WT Cagri - RAMP Cagri -2.93750 0.504 81.2 -5.828 <.0001**

**## WT Cagri - WT sCT -3.31250 0.504 81.2 -6.572 <.0001**

## WT Cagri - RAMP sCT -1.82500 0.504 81.2 -3.621 0.0065

## RAMP Cagri - WT sCT -0.37500 0.504 81.2 -0.744 0.9757

## RAMP Cagri - RAMP sCT 1.11250 0.504 81.2 2.207 0.2458

**## WT sCT - RAMP sCT 1.48750 0.504 81.2 2.951 0.0459**

##

## time = D16:

## contrast estimate SE df t.ratio p.value

## WT Veh - RAMP Veh -1.10476 0.561 81.2 -1.970 0.3685

**## WT Veh - WT Cagri 2.47857 0.522 81.2 4.751 0.0001**

## WT Veh - RAMP Cagri -0.89643 0.522 81.2 -1.718 0.5239

## WT Veh - WT sCT -1.37143 0.522 81.2 -2.629 0.1020

## WT Veh - RAMP sCT -0.09643 0.522 81.2 -0.185 1.0000

## RAMP Veh - WT Cagri 3.58333 0.544 81.2 6.582 <.0001

## RAMP Veh - RAMP Cagri 0.20833 0.544 81.2 0.383 0.9989

## RAMP Veh - WT sCT -0.26667 0.544 81.2 -0.490 0.9964

## RAMP Veh - RAMP sCT 1.00833 0.544 81.2 1.852 0.4388

**## WT Cagri - RAMP Cagri -3.37500 0.504 81.2 -6.696 <.0001**

**## WT Cagri - WT sCT -3.85000 0.504 81.2 -7.639 <.0001**

## WT Cagri - RAMP sCT -2.57500 0.504 81.2 -5.109 <.0001

## RAMP Cagri - WT sCT -0.47500 0.504 81.2 -0.942 0.9342

## RAMP Cagri - RAMP sCT 0.80000 0.504 81.2 1.587 0.6092

## WT sCT - RAMP sCT 1.27500 0.504 81.2 2.530 0.1277

##

## time = D17:

## contrast estimate SE df t.ratio p.value

## WT Veh - RAMP Veh -0.72381 0.561 81.2 -1.291 0.7893

**## WT Veh - WT Cagri 2.48036 0.522 81.2 4.754 0.0001**

## WT Veh - RAMP Cagri -0.48214 0.522 81.2 -0.924 0.9392

**## WT Veh - WT sCT -1.53214 0.522 81.2 -2.937 0.0477**

## WT Veh - RAMP sCT -0.03214 0.522 81.2 -0.062 1.0000

## RAMP Veh - WT Cagri 3.20417 0.544 81.2 5.886 <.0001

## RAMP Veh - RAMP Cagri 0.24167 0.544 81.2 0.444 0.9977

## RAMP Veh - WT sCT -0.80833 0.544 81.2 -1.485 0.6748

## RAMP Veh - RAMP sCT 0.69167 0.544 81.2 1.271 0.8000

**## WT Cagri - RAMP Cagri -2.96250 0.504 81.2 -5.878 <.0001**

**## WT Cagri - WT sCT -4.01250 0.504 81.2 -7.961 <.0001**

## WT Cagri - RAMP sCT -2.51250 0.504 81.2 -4.985 <.0001

## RAMP Cagri - WT sCT -1.05000 0.504 81.2 -2.083 0.3063

## RAMP Cagri - RAMP sCT 0.45000 0.504 81.2 0.893 0.9472

**## WT sCT - RAMP sCT 1.50000 0.504 81.2 2.976 0.0430**

##

## time = D18:

## contrast estimate SE df t.ratio p.value

**## WT Veh - RAMP Veh -0.77619 0.561 81.2 -1.384 0.7364**

**## WT Veh - WT Cagri 2.51964 0.522 81.2 4.830 0.0001**

## WT Veh - RAMP Cagri -0.80536 0.522 81.2 -1.544 0.6373

## WT Veh - WT sCT -1.38036 0.522 81.2 -2.646 0.0980

## WT Veh - RAMP sCT -0.11786 0.522 81.2 -0.226 0.9999

## RAMP Veh - WT Cagri 3.29583 0.544 81.2 6.054 <.0001

## RAMP Veh - RAMP Cagri -0.02917 0.544 81.2 -0.054 1.0000

## RAMP Veh - WT sCT -0.60417 0.544 81.2 -1.110 0.8760

## RAMP Veh - RAMP sCT 0.65833 0.544 81.2 1.209 0.8311

**## WT Cagri - RAMP Cagri -3.32500 0.504 81.2 -6.597 <.0001**

**## WT Cagri - WT sCT -3.90000 0.504 81.2 -7.738 <.0001**

## WT Cagri - RAMP sCT -2.63750 0.504 81.2 -5.233 <.0001

## RAMP Cagri - WT sCT -0.57500 0.504 81.2 -1.141 0.8628

## RAMP Cagri - RAMP sCT 0.68750 0.504 81.2 1.364 0.7481

## WT sCT - RAMP sCT 1.26250 0.504 81.2 2.505 0.1348

##

## time = D19:

## contrast estimate SE df t.ratio p.value

## WT Veh - RAMP Veh -1.06905 0.561 81.2 -1.906 0.4059

**## WT Veh - WT Cagri 1.98929 0.522 81.2 3.813 0.0035**

## WT Veh - RAMP Cagri -0.91071 0.522 81.2 -1.746 0.5062

## WT Veh - WT sCT -1.46071 0.522 81.2 -2.800 0.0677

## WT Veh - RAMP sCT -0.46071 0.522 81.2 -0.883 0.9495

## RAMP Veh - WT Cagri 3.05833 0.544 81.2 5.618 <.0001

## RAMP Veh - RAMP Cagri 0.15833 0.544 81.2 0.291 0.9997

## RAMP Veh - WT sCT -0.39167 0.544 81.2 -0.719 0.9790

## RAMP Veh - RAMP sCT 0.60833 0.544 81.2 1.117 0.8728

**## WT Cagri - RAMP Cagri -2.90000 0.504 81.2 -5.754 <.0001**

**## WT Cagri - WT sCT -3.45000 0.504 81.2 -6.845 <.0001**

## WT Cagri - RAMP sCT -2.45000 0.504 81.2 -4.861 0.0001

## RAMP Cagri - WT sCT -0.55000 0.504 81.2 -1.091 0.8835

## RAMP Cagri - RAMP sCT 0.45000 0.504 81.2 0.893 0.9472

## WT sCT - RAMP sCT 1.00000 0.504 81.2 1.984 0.3604

##

## time = D20:

## contrast estimate SE df t.ratio p.value

## WT Veh - RAMP Veh -0.75000 0.561 81.2 -1.337 0.7634

**## WT Veh - WT Cagri 2.26250 0.522 81.2 4.337 0.0006**

## WT Veh - RAMP Cagri -0.38750 0.522 81.2 -0.743 0.9759

## WT Veh - WT sCT -1.40000 0.522 81.2 -2.684 0.0897

## WT Veh - RAMP sCT 0.02500 0.522 81.2 0.048 1.0000

## RAMP Veh - WT Cagri 3.01250 0.544 81.2 5.534 <.0001

## RAMP Veh - RAMP Cagri 0.36250 0.544 81.2 0.666 0.9852

## RAMP Veh - WT sCT -0.65000 0.544 81.2 -1.194 0.8385

## RAMP Veh - RAMP sCT 0.77500 0.544 81.2 1.424 0.7127

**## WT Cagri - RAMP Cagri -2.65000 0.504 81.2 -5.258 <.0001**

**## WT Cagri - WT sCT -3.66250 0.504 81.2 -7.267 <.0001**

## WT Cagri - RAMP sCT -2.23750 0.504 81.2 -4.439 0.0004

## RAMP Cagri - WT sCT -1.01250 0.504 81.2 -2.009 0.3464

## RAMP Cagri - RAMP sCT 0.41250 0.504 81.2 0.818 0.9633

## WT sCT - RAMP sCT 1.42500 0.504 81.2 2.827 0.0632

##

## time = D21:

## contrast estimate SE df t.ratio p.value

## WT Veh - RAMP Veh -0.96191 0.561 81.2 -1.715 0.5259

**## WT Veh - WT Cagri 2.27143 0.522 81.2 4.354 0.0005**

## WT Veh - RAMP Cagri -0.74107 0.522 81.2 -1.421 0.7146

**## WT Veh - WT sCT -1.72857 0.522 81.2 -3.313 0.0168**

## WT Veh - RAMP sCT -0.79107 0.522 81.2 -1.516 0.6549

## RAMP Veh - WT Cagri 3.23333 0.544 81.2 5.939 <.0001

## RAMP Veh - RAMP Cagri 0.22083 0.544 81.2 0.406 0.9985

## RAMP Veh - WT sCT -0.76667 0.544 81.2 -1.408 0.7219

## RAMP Veh - RAMP sCT 0.17083 0.544 81.2 0.314 0.9996

**## WT Cagri - RAMP Cagri -3.01250 0.504 81.2 -5.977 <.0001**

**## WT Cagri - WT sCT -4.00000 0.504 81.2 -7.936 <.0001**

## WT Cagri - RAMP sCT -3.06250 0.504 81.2 -6.076 <.0001

## RAMP Cagri - WT sCT -0.98750 0.504 81.2 -1.959 0.3746

## RAMP Cagri - RAMP sCT -0.05000 0.504 81.2 -0.099 1.0000

## WT sCT - RAMP sCT 0.93750 0.504 81.2 1.860 0.4340

##

## Degrees-of-freedom method: kenward-roger

## P value adjustment: tukey method for comparing a family of 6 estimates

**Figure 3a: fat mass**

Statistical test: Two-way Anova. Factors: genotype and treatment (Rx)

| Two-way ANOVA | Ordinary |  |  |  |  |
| --- | --- | --- | --- | --- | --- |
| Alpha | 0.05 |  |  |  |  |
|  |  |  |  |  |  |
| Source of Variation | % of total variation | P value | P value summary | Significant? |  |
| Interaction | 6.872 | 0.1960 | ns | No |  |
| Gen | 7.154 | 0.0674 | ns | No |  |
| Rx | 4.218 | 0.3621 | ns | No |  |
|  |  |  |  |  |  |
| ANOVA table | SS (Type III) | DF | MS | F (DFn, DFd) | P value |
| Interaction | 62.65 | 2 | 31.32 | F (2, 40) = 1.698 | P=0.1960 |
| Gen | 65.23 | 1 | 65.23 | F (1, 40) = 3.535 | P=0.0674 |
| Rx | 38.45 | 2 | 19.23 | F (2, 40) = 1.042 | P=0.3621 |
| Residual | 738.0 | 40 | 18.45 |  |  |

**Lean Mass**

Statistical test: Two-way Anova. Factors: genotype and treatment (Rx)

| Two-way ANOVA | Ordinary |  |  |  |  |
| --- | --- | --- | --- | --- | --- |
| Alpha | 0.05 |  |  |  |  |
|  |  |  |  |  |  |
| Source of Variation | % of total variation | P value | P value summary | Significant? |  |
| Interaction | 7.940 | 0.1036 | ns | No |  |
| Gen | 23.76 | 0.0005 | *** | Yes |  |
| Rx | 0.3143 | 0.9095 | ns | No |  |
|  |  |  |  |  |  |
| ANOVA table | SS (Type III) | DF | MS | F (DFn, DFd) | P value |
| Interaction | 8.449 | 2 | 4.224 | F (2, 40) = 2.401 | P=0.1036 |
| Gen | 25.28 | 1 | 25.28 | F (1, 40) = 14.37 | P=0.0005 |
| Rx | 0.3345 | 2 | 0.1672 | F (2, 40) = 0.09504 | P=0.9095 |
| Residual | 70.39 | 40 | 1.760 |  |  |

| Tukey's multiple comparisons test | Predicted (LS) mean diff. | 95.00% CI of diff. | Below threshold? | Summary | Adjusted P Value |
| --- | --- | --- | --- | --- | --- |
|  |  |  |  |  |  |
| WT |  |  |  |  |  |
| Vehicle vs. Cagrilintide | -1.168 | -2.839 to 0.5031 | No | ns | 0.2173 |
| Vehicle vs. sCT | -1.043 | -2.714 to 0.6281 | No | ns | 0.2930 |
| Cagrilintide vs. sCT | 0.1250 | -1.489 to 1.739 | No | ns | 0.9806 |
|  |  |  |  |  |  |
| RAMP 1/3 KO |  |  |  |  |  |
| Vehicle vs. Cagrilintide | 0.8250 | -0.8460 to 2.496 | No | ns | 0.4592 |
| Vehicle vs. sCT | 0.6500 | -1.021 to 2.321 | No | ns | 0.6143 |
| Cagrilintide vs. sCT | -0.1750 | -1.789 to 1.439 | No | ns | 0.9624 |
|  |  |  |  |  |  |
| Vehicle |  |  |  |  |  |
| WT vs. RAMP 1/3 KO | 0.2571 | -1.176 to 1.690 | No | ns | 0.7188 |
|  |  |  |  |  |  |
| Cagrilintide |  |  |  |  |  |
| WT vs. RAMP 1/3 KO | 2.250 | 0.9095 to 3.591 | Yes | ** | 0.0016 |
|  |  |  |  |  |  |
| sCT |  |  |  |  |  |
| WT vs. RAMP 1/3 KO | 1.950 | 0.6095 to 3.291 | Yes | ** | 0.0054 |

**Figure 3b: fat mass % body weight**

Statistical test: Two-way Anova. Factors: genotype and treatment (Rx)

| Two-way ANOVA | Ordinary |  |  |  |  |
| --- | --- | --- | --- | --- | --- |
| Alpha | 0.05 |  |  |  |  |
|  |  |  |  |  |  |
| Source of Variation | % of total variation | P value | P value summary | Significant? |  |
| Interaction | 12.75 | 0.0337 | * | Yes |  |
| Gen | 10.79 | 0.0166 | * | Yes |  |
| RX | 6.007 | 0.1886 | ns | No |  |
|  |  |  |  |  |  |
| ANOVA table | SS (Type III) | DF | MS | F (DFn, DFd) | P value |
| Interaction | 308.3 | 2 | 154.2 | F (2, 40) = 3.694 | P=0.0337 |
| Gen | 260.8 | 1 | 260.8 | F (1, 40) = 6.248 | P=0.0166 |
| RX | 145.2 | 2 | 72.61 | F (2, 40) = 1.740 | P=0.1886 |
| Residual | 1670 | 40 | 41.74 |  |  |

| Tukey's multiple comparisons test | Predicted (LS) mean diff. | 95.00% CI of diff. | Below threshold? | Summary | Adjusted P Value |
| --- | --- | --- | --- | --- | --- |
|  |  |  |  |  |  |
| WT |  |  |  |  |  |
| Vehicle vs. Cagrilintide | 5.734 | -2.404 to 13.87 | No | ns | 0.2122 |
| Vehicle vs. sCT | -0.4911 | -8.629 to 7.647 | No | ns | 0.9882 |
| Cagrilintide vs. sCT | -6.225 | -14.09 to 1.637 | No | ns | 0.1443 |
|  |  |  |  |  |  |
| RAMP 1/3 KO |  |  |  |  |  |
| Vehicle vs. Cagrilintide | -7.114 | -15.25 to 1.024 | No | ns | 0.0970 |
| Vehicle vs. sCT | -7.614 | -15.75 to 0.5238 | No | ns | 0.0707 |
| Cagrilintide vs. sCT | -0.5000 | -8.362 to 7.362 | No | ns | 0.9869 |
|  |  |  |  |  |  |
| Vehicle |  |  |  |  |  |
| WT vs. RAMP 1/3 KO | 1.886 | -5.094 to 8.865 | No | ns | 0.5881 |
|  |  |  |  |  |  |
| Cagrilintide |  |  |  |  |  |
| WT vs. RAMP 1/3 KO | -10.96 | -17.49 to -4.434 | Yes | ** | 0.0016 |
|  |  |  |  |  |  |
| sCT |  |  |  |  |  |
| WT vs. RAMP 1/3 KO | -5.238 | -11.77 to 1.291 | No | ns | 0.1128 |

**Lean Mass % body weight**

Statistical test: Two-way Anova. Factors: genotype and treatment (Rx)

| Two-way ANOVA | Ordinary |  |  |  |  |
| --- | --- | --- | --- | --- | --- |
| Alpha | 0.05 |  |  |  |  |
|  |  |  |  |  |  |
| Source of Variation | % of total variation | P value | P value summary | Significant? |  |
| Interaction | 8.990 | 0.0403 | * | No |  |
| Gen | 25.11 | 0.0002 | *** | Yes |  |
| Rx | 4.438 | 0.2380 | ns | No |  |
|  |  |  |  |  |  |
| ANOVA table | SS (Type III) | DF | MS | F (DFn, DFd) | P value |
| Interaction | 160.4 | 2 | 80.19 | F (2, 40) = 3.2305 | P=0.0403 |
| Gen | 447.9 | 1 | 447.9 | F (1, 40) = 16.84 | P=0.0002 |
| Rx | 79.17 | 2 | 39.58 | F (2, 40) = 1.488 | P=0.2380 |
| Residual | 1064 | 40 | 26.59 |  |  |

| Tukey's multiple comparisons test | Predicted (LS) mean diff. | 95.00% CI of diff. | Below threshold? | Summary | Adjusted P Value |
| --- | --- | --- | --- | --- | --- |
|  |  |  |  |  |  |
| WT |  |  |  |  |  |
| Vehicle vs. Cagrilintide | -5.130 | -11.63 to 1.366 | No | ns | 0.1456 |
| Vehicle vs. sCT | -0.2429 | -6.739 to 6.253 | No | ns | 0.9954 |
| Cagrilintide vs. sCT | 4.888 | -1.388 to 11.16 | No | ns | 0.1531 |
|  |  |  |  |  |  |
| RAMP 1/3 KO |  |  |  |  |  |
| Vehicle vs. Cagrilintide | 4.123 | -2.373 to 10.62 | No | ns | 0.2813 |
| Vehicle vs. sCT | 5.148 | -1.348 to 11.64 | No | ns | 0.1438 |
| Cagrilintide vs. sCT | 1.025 | -5.251 to 7.301 | No | ns | 0.9168 |
|  |  |  |  |  |  |
| Vehicle |  |  |  |  |  |
| WT vs. RAMP 1/3 KO | 1.371 | -4.200 to 6.942 | No | ns | 0.6215 |
|  |  |  |  |  |  |
| Cagrilintide |  |  |  |  |  |
| WT vs. RAMP 1/3 KO | 10.63 | 5.414 to 15.84 | Yes | *** | 0.0002 |
|  |  |  |  |  |  |
| sCT |  |  |  |  |  |
| WT vs. RAMP 1/3 KO | 6.763 | 1.551 to 11.97 | Yes | * | 0.0123 |

**Figure 3c: glucose**

Statistical test: Two-way Anova. Factors: genotype and treatment (Rx)

| Two-way ANOVA | Ordinary |  |  |  |  |
| --- | --- | --- | --- | --- | --- |
| Alpha | 0.05 |  |  |  |  |
|  |  |  |  |  |  |
| Source of Variation | % of total variation | P value | P value summary | Significant? |  |
| Interaction | 1.675 | 0.6329 | ns | No |  |
| Gen | 21.99 | 0.0012 | ** | Yes |  |
| Rx | 3.397 | 0.3997 | ns | No |  |
|  |  |  |  |  |  |
| ANOVA table | SS (Type III) | DF | MS | F (DFn, DFd) | P value |
| Interaction | 0.7544 | 2 | 0.3772 | F (2, 40) = 0.4627 | P=0.6329 |
| Gen | 9.902 | 1 | 9.902 | F (1, 40) = 12.15 | P=0.0012 |
| Rx | 1.530 | 2 | 0.7649 | F (2, 40) = 0.9384 | P=0.3997 |
| Residual | 32.61 | 40 | 0.8151 |  |  |

| Tukey's multiple comparisons test | Predicted (LS) mean diff. | 95.00% CI of diff. | Below threshold? | Summary | Adjusted P Value |
| --- | --- | --- | --- | --- | --- |
|  |  |  |  |  |  |
| WT |  |  |  |  |  |
| Vehicle vs. Cagrilintide | -0.06607 | -1.203 to 1.071 | No | ns | 0.9890 |
| Vehicle vs. sCT | -0.1411 | -1.278 to 0.9962 | No | ns | 0.9511 |
| Cagrilintide vs. sCT | -0.07500 | -1.174 to 1.024 | No | ns | 0.9849 |
|  |  |  |  |  |  |
| RAMP 1/3 KO |  |  |  |  |  |
| Vehicle vs. Cagrilintide | -0.1393 | -1.277 to 0.9980 | No | ns | 0.9523 |
| Vehicle vs. sCT | -0.7143 | -1.852 to 0.4230 | No | ns | 0.2886 |
| Cagrilintide 3 vs. sCT | -0.5750 | -1.674 to 0.5237 | No | ns | 0.4180 |
|  |  |  |  |  |  |
| Vehicle |  |  |  |  |  |
| WT vs. RAMP 1/3 KO | -0.7143 | -1.690 to 0.2611 | No | ns | 0.1467 |
|  |  |  |  |  |  |
| Cagrilintide |  |  |  |  |  |
| WT vs. RAMP 1/3 KO | -0.7875 | -1.700 to 0.1249 | No | ns | 0.0888 |
|  |  |  |  |  |  |
| sCT |  |  |  |  |  |
| WT vs. RAMP 1/3 KO | -1.288 | -2.200 to -0.3751 | Yes | ** | 0.0068 |

**Figure 3d: insulin**

Statistical test: Two-way Anova. Factors: genotype and treatment (Rx)

| Two-way ANOVA | Ordinary |  |  |  |  |
| --- | --- | --- | --- | --- | --- |
| Alpha | 0.05 |  |  |  |  |
|  |  |  |  |  |  |
| Source of Variation | % of total variation | P value | P value summary | Significant? |  |
| Interaction | 24.52 | 0.0040 | ** | Yes |  |
| Gen | 4.305 | 0.1381 | ns | No |  |
| Rx | 8.299 | 0.1241 | ns | No |  |
|  |  |  |  |  |  |
| ANOVA table | SS (Type III) | DF | MS | F (DFn, DFd) | P value |
| Interaction | 18314 | 2 | 9157 | F (2, 32) = 6.585 | P=0.0040 |
| Gen | 3216 | 1 | 3216 | F (1, 32) = 2.313 | P=0.1381 |
| Rx | 6200 | 2 | 3100 | F (2, 32) = 2.229 | P=0.1241 |
| Residual | 44500 | 32 | 1391 |  |  |

| Tukey's multiple comparisons test | Predicted (LS) mean diff. | 95.00% CI of diff. | Below threshold? | Summary | Adjusted P Value |
| --- | --- | --- | --- | --- | --- |
|  |  |  |  |  |  |
| WT |  |  |  |  |  |
| Vehicle vs. Cagrilintide | 45.24 | -5.745 to 96.22 | No | ns | 0.0900 |
| Vehicle vs. sCT | 33.67 | -19.24 to 86.57 | No | ns | 0.2758 |
| Cagrilintide vs. sCT | -11.57 | -62.55 to 39.41 | No | ns | 0.8433 |
|  |  |  |  |  |  |
| RAMP 1/3 KO |  |  |  |  |  |
| Vehicle vs. Cagrilintide | -28.43 | -82.09 to 25.23 | No | ns | 0.4045 |
| Vehicle vs. sCT | -76.29 | -129.9 to -22.63 | Yes | ** | 0.0039 |
| Cagrilintide vs. sCT | -47.86 | -96.84 to 1.126 | No | ns | 0.0566 |
|  |  |  |  |  |  |
| Vehicle |  |  |  |  |  |
| WT vs. RAMP 1/3 KO | 42.67 | -3.329 to 88.66 | No | ns | 0.0679 |
|  |  |  |  |  |  |
| Cagrilintide |  |  |  |  |  |
| WT vs. RAMP 1/3 KO | -31.00 | -71.60 to 9.602 | No | ns | 0.1297 |
|  |  |  |  |  |  |
| sCT |  |  |  |  |  |
| WT vs. RAMP 1/3 KO | -67.29 | -109.5 to -25.03 | Yes | ** | 0.0028 |

**Figure 3e: leptin**

Statistical test: Two-way Anova. Factors: genotype and treatment (Rx)

| Two-way ANOVA | Ordinary |  |  |  |  |
| --- | --- | --- | --- | --- | --- |
| Alpha | 0.05 |  |  |  |  |
|  |  |  |  |  |  |
| Source of Variation | % of total variation | P value | P value summary | Significant? |  |
| Interaction | 31.27 | 0.0019 | ** | Yes |  |
| Gen | 0.06431 | 0.8598 | ns | No |  |
| Rx | 3.430 | 0.4390 | ns | No |  |
|  |  |  |  |  |  |
| ANOVA table | SS (Type III) | DF | MS | F (DFn, DFd) | P value |
| Interaction | 1233 | 2 | 616.5 | F (2, 32) = 7.702 | P=0.0019 |
| Gen | 2.536 | 1 | 2.536 | F (1, 32) = 0.03168 | P=0.8598 |
| Rx | 135.3 | 2 | 67.63 | F (2, 32) = 0.8449 | P=0.4390 |
| Residual | 2562 | 32 | 80.05 |  |  |

| Tukey's multiple comparisons test | Predicted (LS) mean diff. | 95.00% CI of diff. | Below threshold? | Summary | Adjusted P Value |
| --- | --- | --- | --- | --- | --- |
|  |  |  |  |  |  |
| WT |  |  |  |  |  |
| Vehicle vs. Cagrilintide | 14.90 | 2.673 to 27.14 | Yes | * | 0.0142 |
| Vehicle vs. sCT | 5.476 | -6.756 to 17.71 | No | ns | 0.5209 |
| Cagrilintide vs. sCT | -9.429 | -21.18 to 2.323 | No | ns | 0.1357 |
|  |  |  |  |  |  |
| RAMP 1/3 KO |  |  |  |  |  |
| Vehicle vs. Cagrilintide | -13.80 | -27.11 to -0.4868 | Yes | * | 0.0409 |
| Vehicle vs. sCT | -12.66 | -25.53 to 0.2166 | No | ns | 0.0547 |
| Cagrilintide vs. sCT | 1.143 | -11.09 to 13.37 | No | ns | 0.9714 |
|  |  |  |  |  |  |
| Vehicle |  |  |  |  |  |
| WT vs. RAMP 1/3 KO | 16.13 | 5.098 to 27.17 | Yes | ** | 0.0055 |
|  |  |  |  |  |  |
| Cagrilintide |  |  |  |  |  |
| WT vs. RAMP 1/3 KO | -12.57 | -22.71 to -2.432 | Yes | * | 0.0167 |
|  |  |  |  |  |  |
| sCT |  |  |  |  |  |
| WT vs. RAMP 1/3 KO | -2.000 | -11.74 to 7.741 | No | ns | 0.6786 |

**Figure 4b: AP cFos**

Statistical test: Two-way Anova. Factors: genotype and treatment (Rx)

| Two-way ANOVA | Ordinary |  |  |  |  |
| --- | --- | --- | --- | --- | --- |
| Alpha | 0.05 |  |  |  |  |
|  |  |  |  |  |  |
| Source of Variation | % of total variation | P value | P value summary | Significant? |  |
| Interaction | 5.829 | 0.0005 | *** | Yes |  |
| Gen | 0.01067 | 0.8386 | ns | No |  |
| RX | 81.00 | <0.0001 | **** | Yes |  |
|  |  |  |  |  |  |
| ANOVA table | SS (Type III) | DF | MS | F (DFn, DFd) | P value |
| Interaction | 3683 | 3 | 1228 | F (3, 35) = 7.664 | P=0.0005 |
| Gen | 6.745 | 1 | 6.745 | F (1, 35) = 0.04210 | P=0.8386 |
| RX | 51186 | 3 | 17062 | F (3, 35) = 106.5 | P<0.0001 |
| Residual | 5607 | 35 | 160.2 |  |  |

| Tukey's multiple comparisons test | Predicted (LS) mean diff. | 95.00% CI of diff. | Below threshold? | Summary | Adjusted P Value |
| --- | --- | --- | --- | --- | --- |
|  |  |  |  |  |  |
| WT |  |  |  |  |  |
| Vehicle vs. sCT (3 nmol/kg) | -80.99 | -101.7 to -60.32 | Yes | **** | <0.0001 |
| Vehicle vs. sCT (150 nmol/kg) | -72.55 | -95.44 to -49.65 | Yes | **** | <0.0001 |
| Vehicle vs. Cagrilintide (3 nmol/kg) | -48.94 | -70.53 to -27.35 | Yes | **** | <0.0001 |
| sCT (3 nmol/kg) vs. sCT (150 nmol/kg) | 8.442 | -13.59 to 30.48 | No | ns | 0.7313 |
| sCT (3 nmol/kg) vs. Cagrilintide (3 nmol/kg) | 32.05 | 11.38 to 52.72 | Yes | ** | 0.0010 |
| sCT (150 nmol/kg) vs. Cagrilintide (3 nmol/kg) | 23.61 | 0.7071 to 46.50 | Yes | * | 0.0413 |
|  |  |  |  |  |  |
| RAMP 1/3 KO |  |  |  |  |  |
| Vehicle vs. sCT (3 nmol/kg) | -82.16 | -102.8 to -61.49 | Yes | **** | <0.0001 |
| Vehicle vs. sCT (150 nmol/kg) | -97.27 | -117.9 to -76.60 | Yes | **** | <0.0001 |
| Vehicle vs. Cagrilintide (3 nmol/kg) | -20.32 | -40.99 to 0.3459 | No | ns | 0.0554 |
| sCT (3 nmol/kg) vs. sCT (150 nmol/kg) | -15.12 | -34.82 to 4.591 | No | ns | 0.1833 |
| sCT (3 nmol/kg) vs. Cagrilintide (3 nmol/kg) | 61.83 | 42.13 to 81.54 | Yes | **** | <0.0001 |
| sCT (150 nmol/kg) vs. Cagrilintide (3 nmol/kg) | 76.95 | 57.24 to 96.66 | Yes | **** | <0.0001 |
|  |  |  |  |  |  |
| Vehicle |  |  |  |  |  |
| WT vs. RAMP 1/3 KO | 0.1200 | -16.13 to 16.37 | No | ns | 0.9881 |
|  |  |  |  |  |  |
| sCT (3 nmol/kg) |  |  |  |  |  |
| WT vs. RAMP 1/3 KO | -1.050 | -15.88 to 13.78 | No | ns | 0.8866 |
|  |  |  |  |  |  |
| sCT (150 nmol/kg) |  |  |  |  |  |
| WT vs. RAMP 1/3 KO | -24.61 | -41.19 to -8.022 | Yes | ** | 0.0048 |
|  |  |  |  |  |  |
| Cagrilintide (3 nmol/kg) |  |  |  |  |  |
| WT vs. RAMP 1/3 KO | 28.74 | 13.18 to 44.30 | Yes | *** | 0.0006 |

Normality test

| Normality of Residuals |  |  |  |  |
| --- | --- | --- | --- | --- |
| Test name | Statistics | P value | Passed normality test (alpha=0.05)? | P value summary |
| D'Agostino-Pearson omnibus (K2) | 15.23 | 0.0005 | No | *** |
| Anderson-Darling (A2*) | 1.792 | 0.0001 | No | *** |
| Shapiro-Wilk (W) | 0.8822 | 0.0004 | No | *** |
| Kolmogorov-Smirnov (distance) | 0.1755 | 0.0019 | No | ** |

Second Statistical test: Two-way Anova. Factors: genotype and treatment (Rx) after square root transformation and positive normality test

| Two-way ANOVA | Ordinary |  |  |  |  |
| --- | --- | --- | --- | --- | --- |
| Alpha | 0.05 |  |  |  |  |
|  |  |  |  |  |  |
| Source of Variation | % of total variation | P value | P value summary | Significant? |  |
| Interaction | 3.954 | <0.0001 | **** | Yes |  |
| Gen | 0.1615 | 0.2836 | ns | No |  |
| Rx | 88.61 | <0.0001 | **** | Yes |  |
|  |  |  |  |  |  |
| ANOVA table | SS (Type III) | DF | MS | F (DFn, DFd) | P value |
| Interaction | 21.49 | 3 | 7.164 | F (3, 35) = 9.678 | P<0.0001 |
| Gen | 0.8780 | 1 | 0.8780 | F (1, 35) = 1.186 | P=0.2836 |
| Rx | 481.6 | 3 | 160.5 | F (3, 35) = 216.9 | P<0.0001 |
| Residual | 25.91 | 35 | 0.7402 |  |  |
|  |  |  |  |  |  |
| Difference between row means |  |  |  |  |  |
| Predicted (LS) mean of WT | 6.325 |  |  |  |  |
| Predicted (LS) mean of RAMP 1/3 KO | 6.036 |  |  |  |  |
| Difference between predicted means | 0.2885 |  |  |  |  |
| SE of difference | 0.2649 |  |  |  |  |
| 95% CI of difference | -0.2493 to 0.8263 |  |  |  |  |
|  |  |  |  |  |  |
| Normality of Residuals |  |  |  |  |  |
| Test name | Statistics | P value | Passed normality test (alpha=0.05)? | P value summary |  |
| D'Agostino-Pearson omnibus (K2) | 10.02 | 0.0067 | No | ** |  |
| Anderson-Darling (A2*) | 0.6913 | 0.0661 | Yes | ns |  |
| Shapiro-Wilk (W) | 0.9401 | 0.0262 | No | * |  |
| Kolmogorov-Smirnov (distance) | 0.1144 | 0.1000 | Yes | ns |  |

| Tukey's multiple comparisons test | Predicted (LS) mean diff. | 95.00% CI of diff. | Below threshold? | Summary | Adjusted P Value |
| --- | --- | --- | --- | --- | --- |
|  |  |  |  |  |  |
| WT |  |  |  |  |  |
| Vehicle vs. sCT (3 nmol/kg) | -8.131 | -9.536 to -6.726 | Yes | **** | <0.0001 |
| Vehicle vs. sCT (150 nmol/kg) | -7.632 | -9.189 to -6.076 | Yes | **** | <0.0001 |
| Vehicle vs. Cagrilintide (3 nmol/kg) | -6.224 | -7.692 to -4.757 | Yes | **** | <0.0001 |
| sCT (3 nmol/kg) vs. sCT (150 nmol/kg) | 0.4986 | -0.9992 to 1.996 | No | ns | 0.8061 |
| sCT (3 nmol/kg) vs. Cagrilintide (3 nmol/kg) | 1.906 | 0.5012 to 3.311 | Yes | ** | 0.0044 |
| sCT (150 nmol/kg) vs. Cagrilintide (3 nmol/kg) | 1.408 | -0.1489 to 2.964 | No | ns | 0.0882 |
|  |  |  |  |  |  |
| RAMP 1/3 KO |  |  |  |  |  |
| Vehicle vs. sCT (3 nmol/kg) | -8.457 | -9.862 to -7.052 | Yes | **** | <0.0001 |
| Vehicle vs. sCT (150 nmol/kg) | -9.262 | -10.67 to -7.856 | Yes | **** | <0.0001 |
| Vehicle vs. Cagrilintide (3 nmol/kg) | -3.878 | -5.283 to -2.473 | Yes | **** | <0.0001 |
| sCT (3 nmol/kg) vs. sCT (150 nmol/kg) | -0.8046 | -2.144 to 0.5350 | No | ns | 0.3810 |
| sCT (3 nmol/kg) vs. Cagrilintide (3 nmol/kg) | 4.579 | 3.239 to 5.919 | Yes | **** | <0.0001 |
| sCT (150 nmol/kg) vs. Cagrilintide (3 nmol/kg) | 5.384 | 4.044 to 6.723 | Yes | **** | <0.0001 |
|  |  |  |  |  |  |
| Vehicle |  |  |  |  |  |
| WT vs. RAMP 1/3 KO | 0.1908 | -0.9139 to 1.295 | No | ns | 0.7280 |
|  |  |  |  |  |  |
| sCT (3 nmol/kg) |  |  |  |  |  |
| WT vs. RAMP 1/3 KO | -0.1355 | -1.144 to 0.8730 | No | ns | 0.7867 |
|  |  |  |  |  |  |
| sCT (150 nmol/kg) |  |  |  |  |  |
| WT vs. RAMP 1/3 KO | -1.439 | -2.566 to -0.3112 | Yes | * | 0.0139 |
|  |  |  |  |  |  |
| Cagrilintide (3 nmol/kg) |  |  |  |  |  |
| WT vs. RAMP 1/3 KO | 2.537 | 1.480 to 3.595 | Yes | **** | <0.0001 |

**Figure 4c: NTS cFos**

Statistical test: Two-way Anova. Factors: genotype and treatment (Rx)

| Two-way ANOVA | Ordinary |  |  |  |  |
| --- | --- | --- | --- | --- | --- |
| Alpha | 0.05 |  |  |  |  |
|  |  |  |  |  |  |
| Source of Variation | % of total variation | P value | P value summary | Significant? |  |
| Interaction | 1.493 | 0.6687 | ns | No |  |
| Gen | 1.954 | 0.1608 | ns | No |  |
| RX | 63.56 | <0.0001 | **** | Yes |  |
|  |  |  |  |  |  |
| ANOVA table | SS (Type III) | DF | MS | F (DFn, DFd) | P value |
| Interaction | 1301 | 3 | 433.7 | F (3, 33) = 0.5241 | P=0.6687 |
| Gen | 1703 | 1 | 1703 | F (1, 33) = 2.058 | P=0.1608 |
| RX | 55394 | 3 | 18465 | F (3, 33) = 22.32 | P<0.0001 |
| Residual | 27304 | 33 | 827.4 |  |  |

| Tukey's multiple comparisons test | Predicted (LS) mean diff. | 95.00% CI of diff. | Below threshold? | Summary | Adjusted P Value |
| --- | --- | --- | --- | --- | --- |
|  |  |  |  |  |  |
| WT |  |  |  |  |  |
| Vehicle vs. sCT (3 nmol/kg) | -90.46 | -137.6 to -43.35 | Yes | **** | <0.0001 |
| Vehicle vs. sCT (150 nmol/kg) | -106.0 | -158.2 to -53.79 | Yes | **** | <0.0001 |
| Vehicle vs. Cagrilintide (3 nmol/kg) | -54.93 | -102.0 to -7.812 | Yes | * | 0.0172 |
| sCT (3 nmol/kg) vs. sCT (150 nmol/kg) | -15.53 | -65.75 to 34.70 | No | ns | 0.8369 |
| sCT (3 nmol/kg) vs. Cagrilintide (3 nmol/kg) | 35.53 | -9.389 to 80.46 | No | ns | 0.1618 |
| sCT (150 nmol/kg) vs. Cagrilintide (3 nmol/kg) | 51.06 | 0.8342 to 101.3 | Yes | * | 0.0451 |
|  |  |  |  |  |  |
| RAMP 1/3 KO |  |  |  |  |  |
| Vehicle vs. sCT (3 nmol/kg) | -65.39 | -117.6 to -13.20 | Yes | ** | 0.0094 |
| Vehicle vs. sCT (150 nmol/kg) | -101.0 | -153.2 to -48.84 | Yes | **** | <0.0001 |
| Vehicle vs. Cagrilintide (3 nmol/kg) | -29.92 | -80.14 to 20.31 | No | ns | 0.3864 |
| sCT (3 nmol/kg) vs. sCT (150 nmol/kg) | -35.64 | -84.85 to 13.57 | No | ns | 0.2240 |
| sCT (3 nmol/kg) vs. Cagrilintide (3 nmol/kg) | 35.47 | -11.64 to 82.59 | No | ns | 0.1954 |
| sCT (150 nmol/kg) vs. Cagrilintide (3 nmol/kg) | 71.11 | 24.00 to 118.2 | Yes | ** | 0.0014 |
|  |  |  |  |  |  |
| Vehicle |  |  |  |  |  |
| WT vs. RAMP 1/3 KO | -0.7100 | -39.97 to 38.55 | No | ns | 0.9709 |
|  |  |  |  |  |  |
| sCT (3 nmol/kg) |  |  |  |  |  |
| WT vs. RAMP 1/3 KO | 24.36 | -11.08 to 59.80 | No | ns | 0.1713 |
|  |  |  |  |  |  |
| sCT (150 nmol/kg) |  |  |  |  |  |
| WT vs. RAMP 1/3 KO | 4.245 | -35.01 to 43.50 | No | ns | 0.8272 |
|  |  |  |  |  |  |
| Cagrilintide (3 nmol/kg) |  |  |  |  |  |
| WT vs. RAMP 1/3 KO | 24.30 | -9.488 to 58.09 | No | ns | 0.1529 |

**Figure 4d: LPBN cFos**

Statistical test: Two-way Anova. Factors: genotype and treatment (Rx)

|  |  |  |  |  |  |
| --- | --- | --- | --- | --- | --- |
| Two-way ANOVA | Ordinary |  |  |  |  |
| Alpha | 0.05 |  |  |  |  |
|  |  |  |  |  |  |
| Source of Variation | % of total variation | P value | P value summary | Significant? |  |
| Interaction | 3.305 | 0.5524 | ns | No |  |
| Gen | 0.03942 | 0.8742 | ns | No |  |
| RX | 54.00 | <0.0001 | **** | Yes |  |
|  |  |  |  |  |  |
| ANOVA table | SS (Type III) | DF | MS | F (DFn, DFd) | P value |
| Interaction | 1935 | 3 | 645.0 | F (3, 28) = 0.7130 | P=0.5524 |
| Gen | 23.08 | 1 | 23.08 | F (1, 28) = 0.02551 | P=0.8742 |
| RX | 31619 | 3 | 10540 | F (3, 28) = 11.65 | P<0.0001 |
| Residual | 25330 | 28 | 904.6 |  |  |

| Tukey's multiple comparisons test | Predicted (LS) mean diff. | 95.00% CI of diff. | Below threshold? | Summary | Adjusted P Value |
| --- | --- | --- | --- | --- | --- |
|  |  |  |  |  |  |
| WT |  |  |  |  |  |
| Vehicle vs. sCT (3 nmol/kg) | -97.43 | -160.1 to -34.70 | Yes | ** | 0.0012 |
| Vehicle vs. sCT (150 nmol/kg) | -76.50 | -139.2 to -13.78 | Yes | * | 0.0123 |
| Vehicle vs. Cagrilintide (3 nmol/kg) | -59.88 | -119.6 to -0.1156 | Yes | * | 0.0494 |
| sCT (3 nmol/kg) vs. sCT (150 nmol/kg) | 20.93 | -37.14 to 78.99 | No | ns | 0.7597 |
| sCT (3 nmol/kg) vs. Cagrilintide (3 nmol/kg) | 38.35 | -16.74 to 93.43 | No | ns | 0.2508 |
| sCT (150 nmol/kg) vs. Cagrilintide (3 nmol/kg) | 17.42 | -37.67 to 72.51 | No | ns | 0.8235 |
|  |  |  |  |  |  |
| RAMP 1/3 KO |  |  |  |  |  |
| Vehicle vs. sCT (3 nmol/kg) | -60.94 | -112.9 to -9.003 | Yes | * | 0.0168 |
| Vehicle vs. sCT (150 nmol/kg) | -72.14 | -124.1 to -20.20 | Yes | ** | 0.0039 |
| Vehicle vs. Cagrilintide (3 nmol/kg) | -33.18 | -85.12 to 18.76 | No | ns | 0.3208 |
| sCT (3 nmol/kg) vs. sCT (150 nmol/kg) | -11.20 | -63.14 to 40.74 | No | ns | 0.9346 |
| sCT (3 nmol/kg) vs. Cagrilintide (3 nmol/kg) | 27.76 | -24.18 to 79.70 | No | ns | 0.4746 |
| sCT (150 nmol/kg) vs. Cagrilintide (3 nmol/kg) | 38.96 | -12.98 to 90.90 | No | ns | 0.1950 |
|  |  |  |  |  |  |
| Vehicle |  |  |  |  |  |
| WT vs. RAMP 1/3 KO | -15.06 | -60.05 to 29.93 | No | ns | 0.4986 |
|  |  |  |  |  |  |
| sCT (3 nmol/kg) |  |  |  |  |  |
| WT vs. RAMP 1/3 KO | 21.43 | -19.90 to 62.75 | No | ns | 0.2974 |
|  |  |  |  |  |  |
| sCT (150 nmol/kg) |  |  |  |  |  |
| WT vs. RAMP 1/3 KO | -10.70 | -52.03 to 30.63 | No | ns | 0.6001 |
|  |  |  |  |  |  |
| Cagrilintide (3 nmol/kg) |  |  |  |  |  |
| WT vs. RAMP 1/3 KO | 10.84 | -28.13 to 49.81 | No | ns | 0.5733 |

**Figure 5b: AP cFos**

Statistical test: Two-way Anova. Factors: genotype and treatment (Rx)

| Two-way ANOVA | Ordinary |  |  |  |  |
| --- | --- | --- | --- | --- | --- |
| Alpha | 0.05 |  |  |  |  |
|  |  |  |  |  |  |
| Source of Variation | % of total variation | P value | P value summary | Significant? |  |
| Interaction | 5.476 | 0.0353 | * | Yes |  |
| Gen | 4.148 | 0.0824 | ns | No |  |
| RX | 71.71 | <0.0001 | **** | Yes |  |
|  |  |  |  |  |  |
| ANOVA table | SS (Type III) | DF | MS | F (DFn, DFd) | P value |
| Interaction | 7082 | 3 | 2361 | F (3, 34) = 3.205 | P=0.0353 |
| Gen | 5364 | 3 | 1788 | F (3, 34) = 2.428 | P=0.0824 |
| RX | 92737 | 1 | 92737 | F (1, 34) = 125.9 | P<0.0001 |
| Residual | 25044 | 34 | 736.6 |  |  |

| Tukey's multiple comparisons test | Predicted (LS) mean diff. | 95.00% CI of diff. | Below threshold? | Summary | Adjusted P Value |
| --- | --- | --- | --- | --- | --- |
|  |  |  |  |  |  |
| WT |  |  |  |  |  |
| Vehicle vs. Cagrilintide (3 nmol/kg) | -131.1 | -166.7 to -95.48 | Yes | **** | <0.0001 |
|  |  |  |  |  |  |
| RAMP 1/3 KO |  |  |  |  |  |
| Vehicle vs. Cagrilintide (3 nmol/kg) | -57.42 | -90.82 to -24.03 | Yes | ** | 0.0013 |
|  |  |  |  |  |  |
| RAMP 1 KO |  |  |  |  |  |
| Vehicle vs. Cagrilintide (3 nmol/kg) | -100.1 | -133.5 to -66.75 | Yes | **** | <0.0001 |
|  |  |  |  |  |  |
| RAMP 3 KO |  |  |  |  |  |
| Vehicle vs. Cagrilintide (3 nmol/kg) | -90.48 | -125.4 to -55.60 | Yes | **** | <0.0001 |
|  |  |  |  |  |  |
| Vehicle |  |  |  |  |  |
| WT vs. RAMP 1/3 KO | -14.56 | -63.73 to 34.61 | No | ns | 0.8540 |
| WT vs. RAMP 1 KO | -19.43 | -66.75 to 27.88 | No | ns | 0.6864 |
| WT vs. RAMP 3 KO | -25.72 | -74.89 to 23.45 | No | ns | 0.5003 |
| RAMP 1/3 KO vs. RAMP 1 KO | -4.873 | -49.26 to 39.51 | No | ns | 0.9908 |
| RAMP 1/3 KO vs. RAMP 3 KO | -11.16 | -57.52 to 35.20 | No | ns | 0.9148 |
| RAMP 1 KO vs. RAMP 3 KO | -6.287 | -50.67 to 38.10 | No | ns | 0.9806 |
|  |  |  |  |  |  |
| Cagrilintide (3 nmol/kg) |  |  |  |  |  |
| WT vs. RAMP 1/3 KO | 59.10 | 16.78 to 101.4 | Yes | ** | 0.0033 |
| WT vs. RAMP 1 KO | 11.50 | -32.88 to 55.89 | No | ns | 0.8964 |
| WT vs. RAMP 3 KO | 14.88 | -29.50 to 59.27 | No | ns | 0.8019 |
| RAMP 1/3 KO vs. RAMP 1 KO | -47.60 | -91.98 to -3.211 | Yes | * | 0.0317 |
| RAMP 1/3 KO vs. RAMP 3 KO | -44.22 | -88.60 to 0.1687 | No | ns | 0.0512 |
| RAMP 1 KO vs. RAMP 3 KO | 3.380 | -42.98 to 49.74 | No | ns | 0.9972 |

**Figure 5c: NTS cFos**

Statistical test: Two-way Anova. Factors: genotype and treatment (Rx)

| Two-way ANOVA | Ordinary |  |  |  |  |
| --- | --- | --- | --- | --- | --- |
| Alpha | 0.05 |  |  |  |  |
|  |  |  |  |  |  |
| Source of Variation | % of total variation | P value | P value summary | Significant? |  |
| Interaction | 5.029 | 0.1914 | ns | No |  |
| Gen | 9.737 | 0.0346 | * | Yes |  |
| RX | 54.07 | <0.0001 | **** | Yes |  |
|  |  |  |  |  |  |
| ANOVA table | SS (Type III) | DF | MS | F (DFn, DFd) | P value |
| Interaction | 23190 | 3 | 7730 | F (3, 32) = 1.678 | P=0.1914 |
| Gen | 44900 | 3 | 14967 | F (3, 32) = 3.248 | P=0.0346 |
| RX | 249322 | 1 | 249322 | F (1, 32) = 54.11 | P<0.0001 |
| Residual | 147436 | 32 | 4607 |  |  |

| Tukey's multiple comparisons test | Predicted (LS) mean diff. | 95.00% CI of diff. | Below threshold? | Summary | Adjusted P Value |
| --- | --- | --- | --- | --- | --- |
|  |  |  |  |  |  |
| WT |  |  |  |  |  |
| Vehicle vs. Cagri (3 nmol/kg) | -238.8 | -331.6 to -146.1 | Yes | **** | <0.0001 |
|  |  |  |  |  |  |
| RAMP 1/3 KO |  |  |  |  |  |
| Vehicle vs. Cagri (3 nmol/kg) | -142.0 | -225.7 to -58.25 | Yes | ** | 0.0016 |
|  |  |  |  |  |  |
| RAMP 1 KO |  |  |  |  |  |
| Vehicle vs. Cagri (3 nmol/kg) | -101.7 | -189.1 to -14.24 | Yes | * | 0.0241 |
|  |  |  |  |  |  |
| RAMP 3 KO |  |  |  |  |  |
| Vehicle vs. Cagri (3 nmol/kg) | -152.4 | -239.8 to -64.96 | Yes | ** | 0.0012 |
|  |  |  |  |  |  |
| Vehicle |  |  |  |  |  |
| WT vs. RAMP 1/3 KO | 5.515 | -117.9 to 128.9 | No | ns | 0.9994 |
| WT vs. RAMP 1 KO | -9.585 | -133.0 to 113.8 | No | ns | 0.9966 |
| WT vs. RAMP 3 KO | -61.41 | -184.8 to 61.96 | No | ns | 0.5398 |
| RAMP 1/3 KO vs. RAMP 1 KO | -15.10 | -131.4 to 101.2 | No | ns | 0.9848 |
| RAMP 1/3 KO vs. RAMP 3 KO | -66.92 | -183.2 to 49.39 | No | ns | 0.4157 |
| RAMP 1 KO vs. RAMP 3 KO | -51.82 | -168.1 to 64.49 | No | ns | 0.6269 |
|  |  |  |  |  |  |
| Cagri (3 nmol/kg) |  |  |  |  |  |
| WT vs. RAMP 1/3 KO | 102.4 | -8.993 to 213.7 | No | ns | 0.0807 |
| WT vs. RAMP 1 KO | 127.6 | 11.25 to 243.9 | Yes | * | 0.0272 |
| WT vs. RAMP 3 KO | 25.02 | -91.29 to 141.3 | No | ns | 0.9365 |
| RAMP 1/3 KO vs. RAMP 1 KO | 25.19 | -86.17 to 136.6 | No | ns | 0.9272 |
| RAMP 1/3 KO vs. RAMP 3 KO | -77.35 | -188.7 to 34.01 | No | ns | 0.2558 |
| RAMP 1 KO vs. RAMP 3 KO | -102.5 | -218.9 to 13.77 | No | ns | 0.0998 |
